# Creating molecular complexity in the chemoenzymatic synthesis of chlorothricin analogues using tandem Diels–Alderases

**DOI:** 10.1039/d6ob00728g

**Published:** 2026-06-17

**Authors:** Andrew J. Devine, Monserrat Manzo-Ruiz, Catherine R. Back, Katja Zorn, Martin A. Hayes, Paul R. Race, Christine L. Willis

**Affiliations:** a School of Chemistry, University of Bristol Bristol BS8 1TS UK chris.willis@bristol.ac.uk; b School of Natural and Environmental Sciences, Newcastle University Newcastle Upon Tyne NE1 7RU UK paul.race1@newcastle.ac.uk; c School of Biochemistry, University of Bristol Bristol BS8 1TD UK; d Compound Synthesis and Management, Discovery Sciences, Biopharmaceuticals R&D, AstraZeneca Pepparedsleden 1 SE-431 83 Mölndal Sweden; e School of Chemistry and Molecular Biosciences, The University of Queensland St Lucia QLD 4067 Australia

## Abstract

Chlorothricin is a polyketide-derived natural product isolated from *Streptomyces antibioticus*. It possesses an elaborate pentacyclic aglycone core which incorporates a spirotetronic acid moiety, linked to a *trans*-decalin system, embedded within a macrocycle. Using synthetic substrate analogues and purified recombinant proteins, here we demonstrate that assembly of this scaffold proceeds *via* sequential biocatalytic Diels–Alder reactions, promoted by the enzymes ChlE3 and ChlL. Both Diels–Alderases exhibit sufficiently relaxed substrate selectivity to facilitate access to non-natural chlorothricin analogues *via* biotransformations. The X-ray crystal structure of ChlE3 reveals the molecular basis of decalin formation by this enzyme. Harnessing this enzymatic cascade in biocatalysis could provide a valuable biomimetic route to both natural and non-natural spirotetronates, and the work described herein lays the foundation for application of these enzymes in chemoenzymatic syntheses of complex products.

## Abstract 2

Chemoenzymatic assembly of the pentacyclic core of the antibiotic chlorothricin uses synthetic substrates and the enzymes ChlE3 and ChlL to create 2 rings and 4 new stereocentres. ChlL alone catalyses formation of novel spirocyclic products.

## Graphical abstract

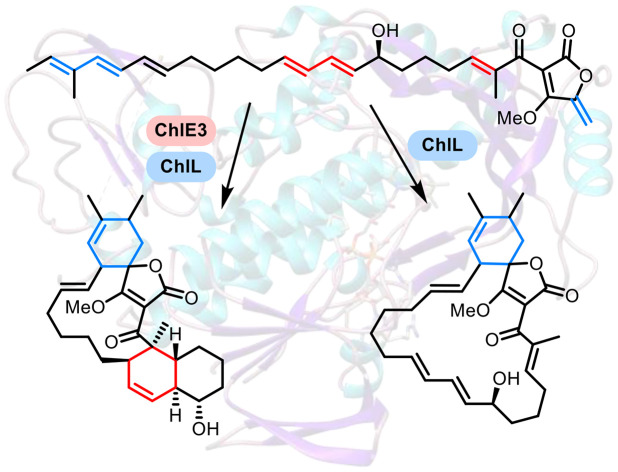

## Introduction

**Scheme 1:**
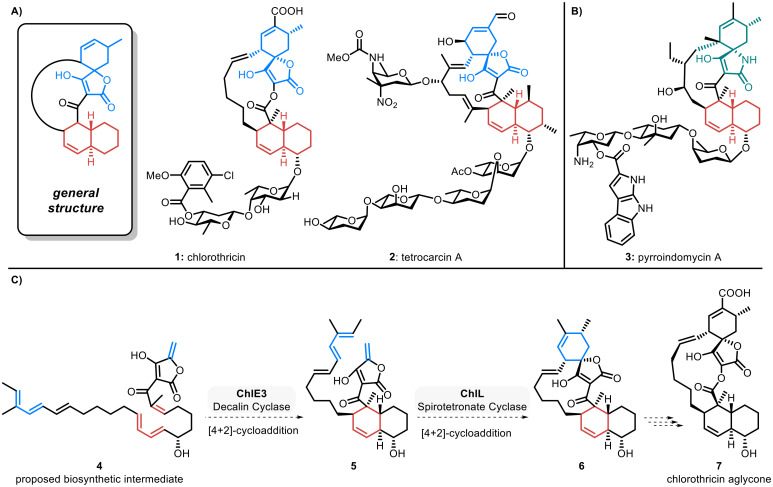
(A) General structure of class II spirotetronates and two representative examples. (B) Structure of pyrroindomycin A, highlighting similarities with class II spirotetronates. (C) Proposed tandem [4 + 2]-cycloadditions in chlorothricin biosynthesis.

Class II spirotetronates are a sub-family of spirotetronate polyketides that possess a characteristic pentacyclic structure, comprising a spirotetronic acid moiety and a *trans*-decalin ring system, united by a macrocycle of varying size. Examples of this class of molecules include both chlorothricin 1 and tetrocarcin A 2 (Scheme 1A).^1^ These natural products have been isolated from marine and terrestrial actinomycetes, and have been found to exhibit a wealth of important biological activities, including acting as antimicrobial, anticancer, antiparasitic and antiviral agents.^2–5^ Despite the clear pharmacological potential of these compounds and analogues thereof, there remains a paucity of studies exploring the biological significance of their conserved pentacyclic core. Such investigations have been hindered by the considerable challenge of accessing and selectively modifying this complex scaffold. However, these issues could be circumvented by the adoption of a chemoenzymatic approach, enabling the efficient construction of the class II spirotetronate pentacyclic core.

In the related pyrroindomycin spirotetramates (Scheme 1B), it has been shown that the characteristic *trans*-decalin and spirocycle have their biosynthetic origins in two consecutive enzymatic [4 + 2]-cycloadditions. First the FAD-dependent enzyme PyrE3 catalyses formation of the decalin and then the β-barrel cyclase PyrI4 creates the macrocycle.^6–11^ Using isolated biosynthetic intermediates along with wild-type and engineered proteins it has been shown that these two enzymes each possess restrictive substrate scope. Interestingly, homologues of PyrI4 and PyrE3 have been identified in class II spirotetronate gene clusters (Table S1), however, to date these enzymes have not been subjected to detailed characterisation, and much remains to be learned about their potential value as biocatalysts.^12–15^

For the studies described herein we selected enzymes from the chlorothricin pathway of *Streptomyces antibioticus*.^16^ The chlorothricin biosynthetic gene cluster (*chl*) was characterised in 2006 and houses the candidate cyclase genes *chlL* and *chlE3*, which belong to the spirotetronate cyclase and decalin cyclase families respectively (Scheme 1C).^15^ Using a combination of chemical synthesis to prepare linear substrates, *in vitro* enzyme assays and X-ray crystallography, we herein identify and characterise the explicit roles of the two [4 + 2]-cyclases that are responsible for catalysing construction of the pentacyclic core of chlorothricin, and in doing so establish the foundations for a chemoenzymatic route towards class II spirotetronates. Furthermore, we extend these studies to the generation of non-natural spirotetronates, demonstrating the broader synthetic utility of chlorothricin pathway Diels–Alderases.

## Results and discussion

Bioinformatic analysis of the *chl* gene cluster identified the genes *chlE3* and *chlL* as encoding proteins with significant amino acid sequence identity to known [4 + 2]-cyclases. ChlE3 possess 47–54% sequence conservation to known FAD dependent decalin forming enzymes, with ChlL possessing 24–47% sequence identity to known spirotetronate cyclases. Based on these findings, codon optimised (for *E. coli*) *chlE3* and *chlL* genes were commercially sourced and cloned into the protein expression vector pET29b, with the resulting plasmids used to facilitate the recombinant overexpression of ChlE3 and ChlL in *E. coli* BL21 (DE3) cells. The resulting proteins were purified to homogeneity using nickel affinity chromatography followed by size-exclusion chromatography (SEC). Both proteins were found to be of >95% purity as assessed by SDS-PAGE analysis (Fig. S1). SEC profiles were consistent with both ChlE3 and ChlL adopting dimeric states in solution (Fig. S1). ChlE3 exhibited a vivid yellow colour, consistent with the presence of a bound flavin cofactor.

**Scheme 2:**
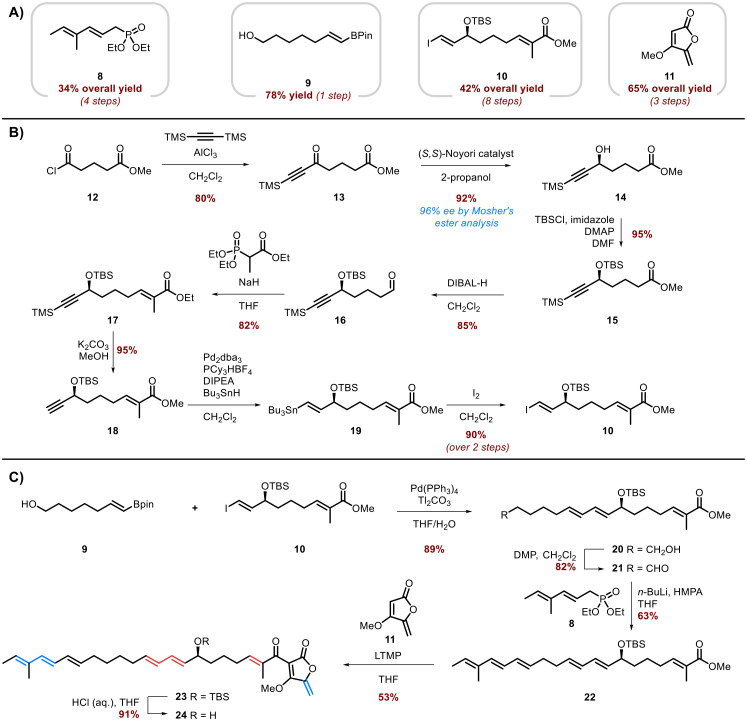
Synthesis of linear cycloaddition substrate 24. (A) Fragments used in the preparation of **24**. (B) Synthetic route to ester **10**. (C) Completing the total synthesis of **24**.

With the proteins in hand, our next goal was to develop a flexible strategy for the synthesis of linear enzyme substrates. The initial target 24 (Scheme 2) was the *O*-methyl analogue of the proposed authentic natural cycloaddition substrate 4, as such derivatives tend to be more stable than the corresponding tetronic acids. Furthermore, previous work within our group has shown that *O*-methyl tetronate derivatives are accepted as substrates by related cyclase enzymes.^17^ The linear polyene tetronate 24 contains the proposed diene and dienophile pairs for both decalin and spirotetronate formation. The target was assembled from the four fragments 8, 9, 10 and 11, which could be readily modified for the synthesis of analogues (Scheme 2A). Fragments 8, 9 and 11 were all prepared in good yields using established methods (Fig. S2). Envisaging forging the central diene *via* Suzuki–Miyaura coupling, vinyl iodide 10 was prepared in 8 steps from commercially available 4-(chloroformyl)butyrate 12 (Scheme 2B). To begin, ester 12 was converted to the acetylenic ketone 13*via* a Friedel–Crafts type reaction with bis(trimethylsilyl)acetylene. Asymmetric transfer hydrogenation with Noyori's ruthenium diamine catalyst gave the (*S*)-configured propargylic alcohol 14 in 92% yield and 96% ee.^18,19^ Following TBS (*tert*-butyldimethylsilyl) protection, reduction of ester 15 to aldehyde 16 with diisobutylaluminium hydride (DIBAL-H) and subsequent Horner–Wadswoth–Emmons (HWE) reaction with triethyl 2-phosphonopropionate gave methyl branched ester 17 in 70% yield. TMS deprotection with K_2_CO_3_/MeOH proceeded cleanly with concomitant transesterification to give terminal alkyne 18, which was then converted to vinyl iodide 10 in 90% yield *via* hydrostannation with Chong's Pd(0)/PCy_3_ system.^20^ To generate the central diene *via* Suzuki–Miyaura coupling of boronic ester 9 and vinyl iodide 10, we turned to conditions first reported by Markó with Tl_2_CO_3_ as the basic additive, which had previously been exploited successfully within our group.^21,22^ Gratifyingly, this method was found to be highly effective at uniting 9 and 10 to furnish (*E*,*E*)-diene 20 in 89% yield (Scheme 2C). Oxidation of the coupling product with Dess Martin periodinane (DMP) and reaction of the resulting aldehyde 21 with lithiated phosphonate 8 afforded the methyl branched triene 22. The tetronate ring was then installed *via* reaction of tetronate 11 with base (lithium 2,2,6,6-tetramethylpiperidine, LTMP) followed by addition to methyl ester 22.^23^ A variety of conditions were investigated for the final deprotection step, with an HCl/THF system proving the most effective. Careful monitoring of the reaction by TLC was required to suppress side-product formation, and the optimised procedure delivered alcohol 24 in 91% yield. Alcohol 24 was prone to decomposition, with significant degradation observed when stored neat for short periods (<1 h). Stability was improved when stored as a solution, and it was found that a dilute CH_2_Cl_2_ solution of 24 remained unchanged after one-month when stored at −20 °C.

**Fig. 1:**
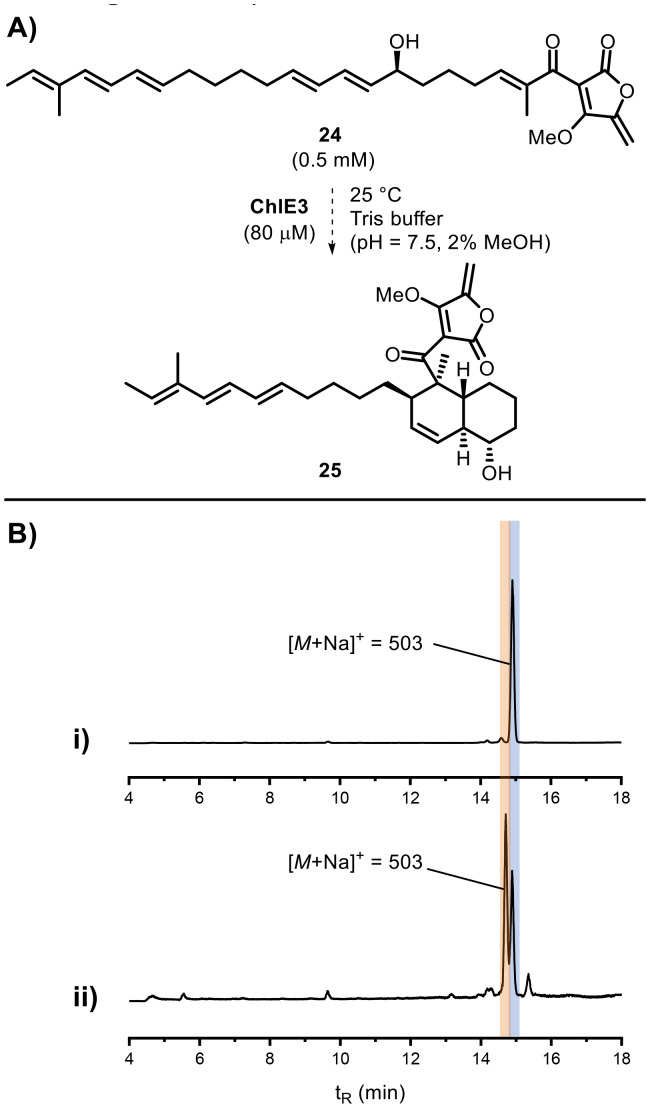
(A) *In vitro* assay with 24 and recombinant ChlE3. (B) (i) LC-MS ELSD trace of the EtOAc extract of control reaction, 24 (0.5 mM) incubated in Tris buffer, 2 h. (ii) LC-MS ELSD trace of EtOAc extract of ChlE3 (80 µM) enzymatic reaction with 24 (0.5 mM), 2 h.

With both proposed cyclases and synthetic substrate 24 in hand the function of these enzymes was probed *via in vitro* assays. We began our functional studies of the [4 + 2]-cyclase pair by examining the proposed chlorothricin decalin cyclase, ChlE3. Tetronate 24 (0.5 mM) was incubated with recombinant ChlE3 in Tris buffer at 25 °C for 2 hours. The reaction was terminated by the addition of ice-cold acetonitrile and the organic extracts were analysed by reverse-phase LC-MS. A new peak (*t*_R_ = 14.7) was apparent by LC-MS analysis of the assay extracts at a retention time close to the substrate 24 (Fig. 1). No trace of this peak was observed in the control assay which lacked enzyme, in accord with a ChlE3-catalysed reaction leading to a new product.

**Fig. 2:**
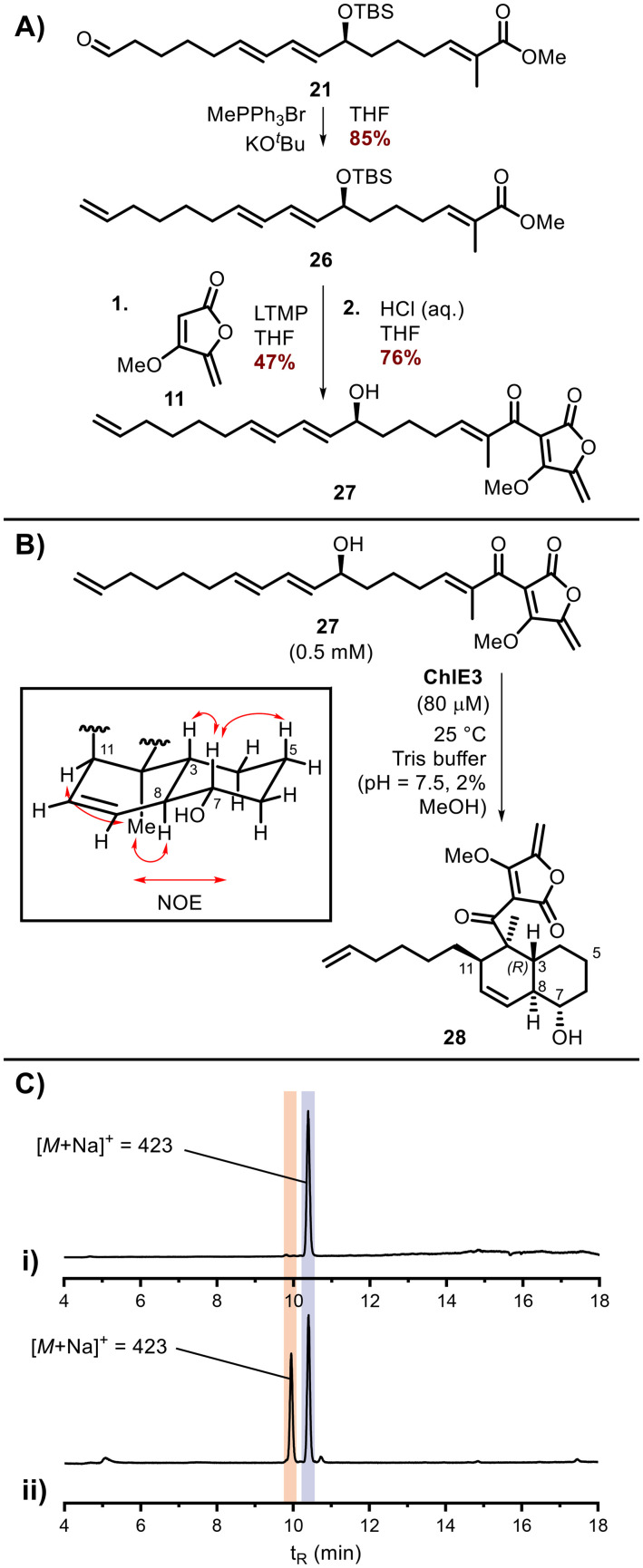
(A) Synthesis of truncated substrate analogue 27. (B) *In vitro* assay with 27 and recombinant ChlE3. (C) (i) LC-MS ELSD trace of the EtOAc extract of control reaction, 27 (0.5 mM) incubated in Tris buffer, 2 h. (ii) LC-MS ELSD trace of EtOAc extract of ChlE3 (80 µM) enzymatic reaction with 27 (0.5 mM).

The MS profile of the product ([*M* + Na]^+^ = 503, Fig. S3) resembled that of the substrate and indicated a species with the same molecular weight (*M* = 480), consistent with the substrate having undergone [4 + 2]-cycloaddition. To confirm the identity of the new product, purification of the mixture was undertaken, however, all efforts to isolate the new species on both normal- and reverse-phase HPLC systems were thwarted by the instability of the assay product leading to degradation. In the course of our synthetic studies, we had observed that compounds containing the branched triene chain terminus were prone to degradation during attempted purification by either chromatography on silica or HPLC. Speculating a similar effect was hindering our efforts to isolate the assay product, to characterise ChlE3 activity, we instead turned to the synthesis of the truncated methylene tail substrate 27 which lacked this problematic triene (Fig. 2A). Pleasingly, 27 was readily accepted as a substrate by ChlE3, with LC-MS analysis of the assay extracts showing a new species with the same mass as 27 (Fig. 2), again consistent with an intramolecular cycloaddition.

The enzymatic reaction was scaled up, and in this case, purification of the new species by reverse-phase HPLC proceeded smoothly, giving access to the pure assay product. Full structural characterisation by ^1^H, ^13^C and 2D NMR revealed the connectivity of the product to be consistent with decalin 28 (Fig. 2B). Initial insights into the stereochemistry were provided by inspection of the 7-H signal *δ*_H_ (CDCl_3_) 3.36 (1H, td, *J* 10.0, 4.0), which indicated an axial orientation and thus an *anti* relationship with the adjacent 8-H. This signal was in accord with the corresponding 7-H resonance previously reported for 24-*O*-methyl chlorothricolide (*δ*_H_ (CDCl_3_) 3.34 (1H, td, *J* 10.0, 4.0)).^24^ 2D NOESY NMR was used to gain further insights into the stereochemistry of the product. An NOE was observed between 7-H and 3-H, indicating their position on the same face of the decalin and hence the (*R*)-configuration of C-3. No NOE was observed between 3-H and 8-H. NOEs between the 2-methyl group and both 8-H and 11-H, indicated the location of these protons on the opposite face of the decalin to 7-H and 3-H, confirming the presence of *trans*-decalin 28 and thus the activity of ChlE3 as a selective *trans*-decalin cyclase. Notably, attempted thermal cycloaddition of 27 by refluxing in toluene gave a complex mixture, of which 28 was only a minor component, illustrating the potential value of enzymatic catalysis for selective decalin synthesis.

**Fig. 3:**
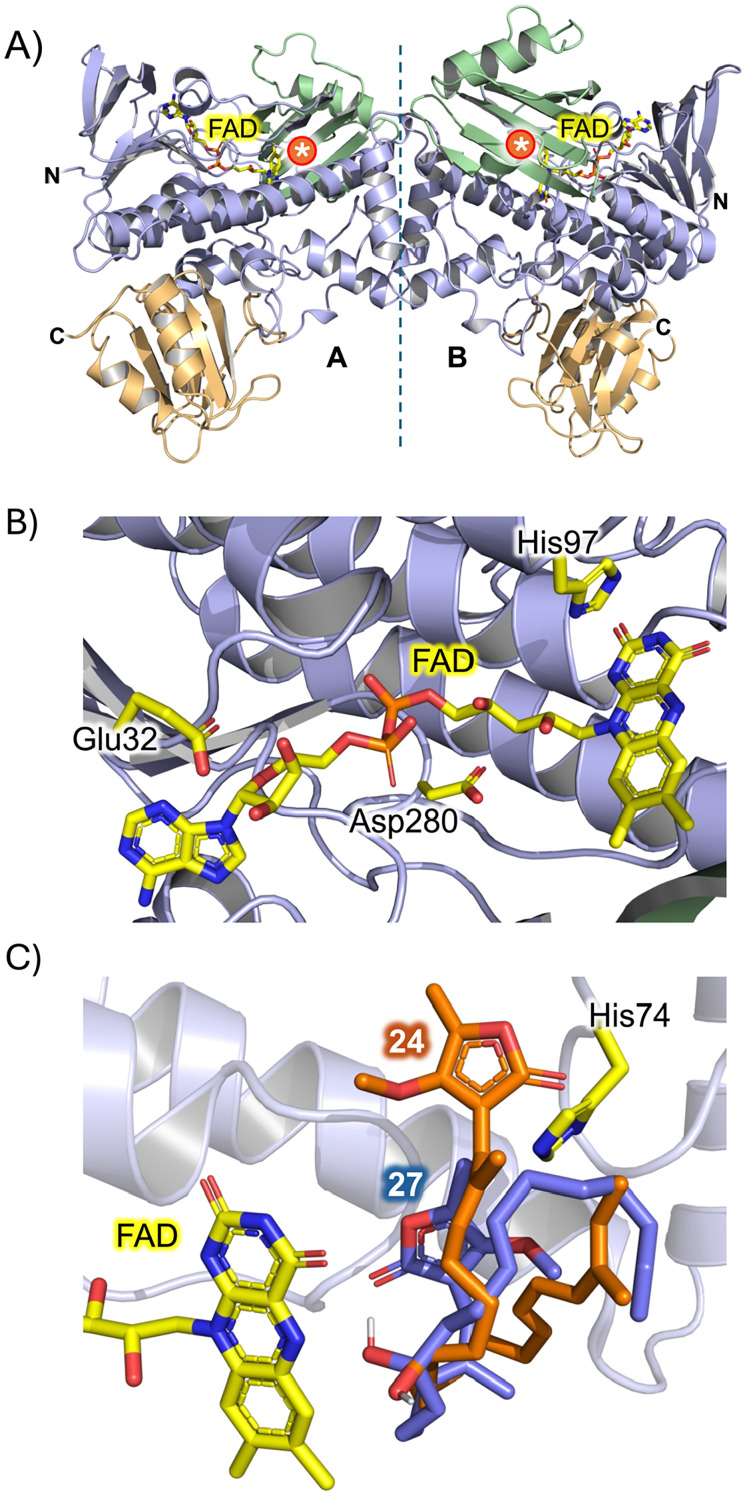
(A) Crystal structure of the ChlE3 dimer showing the overall fold of the enzyme with domains highlighted as follows; ββα sandwich domain (blue), αβα sandwich domain (green), and thioredoxin-like domain (orange). The location of the FAD cofactor and enzyme active site (white asterisk) are indicated. (B) ChlE3 active site showing the bound FAD molecule and conserved amino acids Glu32, His97, and Asp280. The loop region spanning residues Arg33 to Ser48 has been omitted for improved clarity. (C) View of the ChlE3 active site showing the predicted binding modes of 24 and 27.

To provide a structural framework for the ChlE3 catalysed reaction purified recombinant protein was subjected to crystallisation screening, with resulting crystals undergoing X-ray diffraction analysis at Diamond Light Source, UK. The structure of ChlE3 (PDB 9SRP)‡‡The atomic coordinates and structure factors (code PDB 9SRP) have been deposited in the Protein Data Bank. was determined to a resolution of 1.84 Å by molecular replacement, employing that of PyrE3 (51% identity; PDB 5XGV) as the search model (Table S2).^9^ ChlE3 is dimeric, with each monomer adopting a distinct three-domain architecture, comprising a Rossmann-like 3-layer (ββα) sandwich domain, which houses the enzyme active site, a 3-layer (αβα) sandwich domain, and a thioredoxin-like domain (Fig. 3A). The dimer interface is extensive and is contributed to by a combination of electrostatic and hydrophobic interactions. Each monomer within the dimer houses a single non-covalently bound molecule of FAD, which is stabilised *via* an extensive network of electrostatic interactions. These include polar contacts to the flavin ribityl chain, phosphate groups and the ribose moiety. The isoalloxazine ring of the flavin cofactor occupies a highly positively charged pocket, which is large enough to accommodate both the FAD tricyclic ring system and the enzyme substrate, and which constitutes the active site of ChlE3. The observed positive charge is a reported requirement for promotion of the Diels–Alder reaction catalysed by the ChlE3 homologue PyrE3.^8^ The explicit role of the bound flavin cofactor remains to be unambiguously established, however, in ChlE3 homologues it has been suggested to play a role in substrate binding *via* hydrogen-bonding interactions.^7^ Of the ChlE3 residues that are universally conserved in other decalin forming [4 + 2] cyclases (Pro12, Glu32, His97, Asp280, and Pro287), only Glu32, His97 and Asp280 reside within the enzyme active site (Fig. 3B). To confirm their explicit roles in catalysis both Glu32 and Asp280 were mutated to alanine residues, however, this exclusively yielded unfolded protein precluding further analysis.

To investigate the ChlE3 catalysed reaction in greater detail we next performed molecular docking studies using AutoDock Vina^25^ and the synthetic substrates 24 and 27 (Fig. 3C). In both instances the substrate reactive pose sits in close proximity to the flavin co-factor with the substrate diene positioned next to the side chain of residue Arg214. In the ChlE3-24 complex the substrate dienophile resides parallel to the residues Leu44-Ile46, whereas in the ChlE3-27 complex the dienophile is positioned next to Phe286-Leu288. Both docked poses are stabilised *via* a hydrogen bond to the sidechain of His74. In the ChlE3-24 complex this interaction forms between the substrate ketonic carbonyl neighbouring the tetronate ring and His74; a binding mode analogous to that reported previously in both PyrE3 and TedJ.^7,26^ In the ChlE3-27 complex the equivalent interaction forms between the OMe group of the tetronate moiety and His74. Both poses position the substrate C3 sidechain pointing outwards from the enzyme active site prohibiting interaction between enzyme and substrate in this region and indicating that synthetic modifications to this portion of the substrate are unlikely to negatively impact catalysis. Inspection of the ChlE3-24 and ChlE3-27 complexes indicates that steric constraints imposed by the side chains of residues comprising loop regions Gly43-Ser48, Pro71-Trp79 and Phe286-Gln291, appear critical in defining the stereochemical outcome of the enzyme catalysed reaction.

**Fig. 4:**
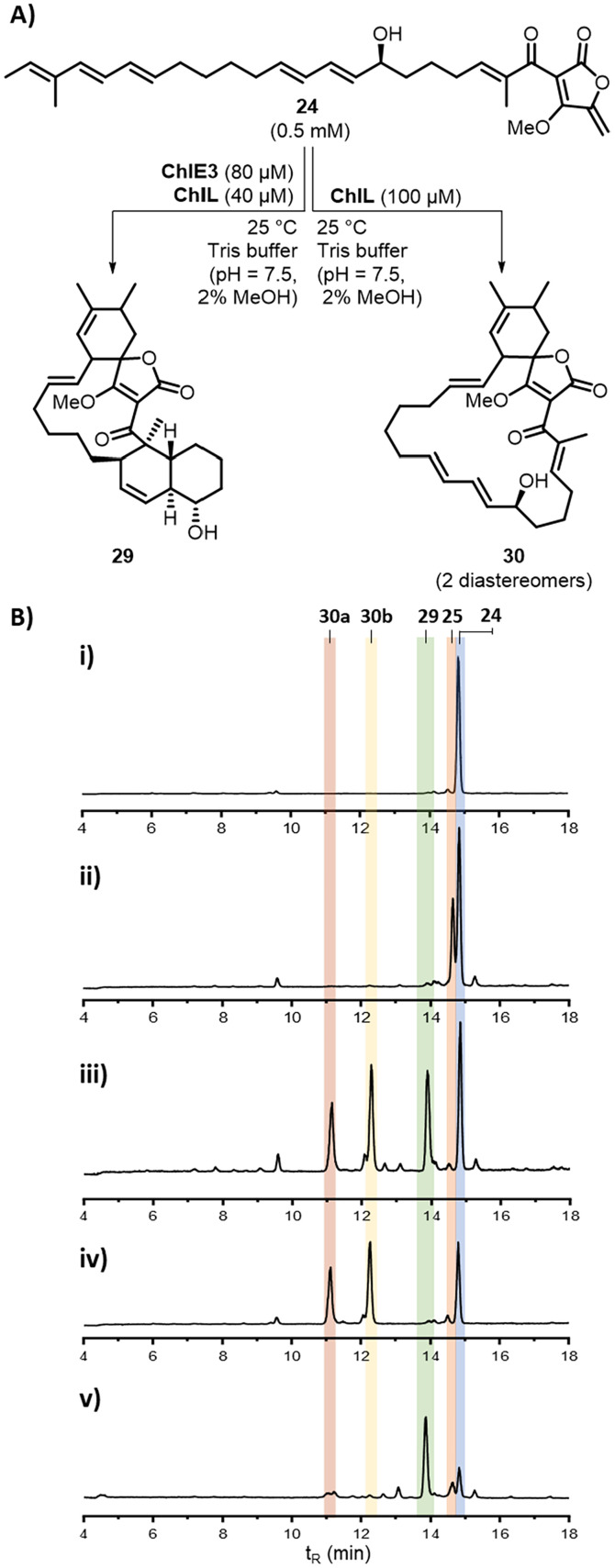
(A) *In vitro* assays with 24, ChlE3 and ChlL. (B) LC-MS ELSD traces of the EtOAc extracts of: (i) control reaction, 24 (0.5 mM) incubated in Tris buffer, 2 h (ii) control reaction with only ChlE3 (80 µM) and 24 (0.5 mM), 2 h. NB we noted ChlE3 activity declined rapidly on storage hence the disparity between the conversion in (B) and in Fig. 3B. (iii) ChlE3 (80 µM) and ChlL (100 µM) tandem enzymatic reaction with 24 (0.5 mM), 2 h (iv) ChlL (100 µM) enzymatic reaction with 24 (0.5 mM), 2 h (v) ChlE3 (80 µM) and ChlL (40 µM) tandem enzymatic reaction with 24 (0.5 mM), 2 h. NB data have been standardised to account for minor retention time drifts.

With good insights into the structure and function of ChlE3, we turned our attention to exploring the key [4 + 2]-cyclisation cascade that is proposed to lead to the pentacyclic core of the class II spirotetronates catalysed by both ChlE3 and the putative spirotetronate cyclase ChlL. To reconstitute the enzymatic sequence *in vitro*, synthetic substrate 24 was incubated with recombinant ChlE3 and ChlL in Tris buffer at 25 °C (Fig. 4A). Three new product peaks were apparent by LC-MS analysis of the assay extract (Fig. 4B-iii, *t*_R_ = 11.1, 12.3 and 13.9). None of the new peaks were present in the no-enzyme control (Fig. 4B-i) or in assays incorporating ChlE3 only (Fig. 4B-ii), indicating their formation by a ChlL-dependent enzymatic processes. Moreover, the peak corresponding to the previously observed ChlE3 product 28 was not present, which is consistent with the expectation that this would serve as a substrate for ChlL. All three products had the same molecular weight as the substrate and the ChlE3 assay product, again indicative of intramolecular cyclisations or rearrangements having occurred.

Next, we carried out an enzymatic reaction with ChlL and 24. We had not anticipated 24 to be a substrate for ChlL as Liu and co-workers had reported that the analogous linear precursor from the pyrroindomycin pathway was not accepted by the native spirotetramate cyclase PyrI4.^6^ However, interestingly on LC-MS analysis of the assay extracts of the enzymatic reaction, two of the three new products observed in the dual enzyme assay were clearly visible (Fig. 4B-iv, *t*_R_ = 11.1 and *t*_R_ = 12.3). Intrigued by this unexpected activity, samples of the two new reaction products were purified by RP-HPLC and characterised by NMR. Both new compounds were closely related isomers that were derived from 24*via* modification of the substrate's π-systems. The ^1^H, and 2D NMR data revealed the two new compounds were isomers of the 21-membered spirotetronate macrocycle 30 (Fig. 4A) arising from [4 + 2]-cycloaddition between the *exo*-methylene tetronate and the terminal diene of the linear precursor. The apparent lack of selectivity in the transformation suggests that this is not the natural function of ChlL in chlorothricin biosynthesis. However, this result nonetheless represents a remarkable enzymatic transformation given the remote nature of the diene and dienophile pair and suggests ChlL is catalysing [4 + 2]-cycloadditions of the tetronate and terminal diene, even in the case of what is presumed to be a non-natural substrate. These observations further add to the growing repertoire of unnatural cyclisation activity reported for spirotetronate cyclases.^27,28^

Having characterised the isomeric macrocycles 30a and 30b, this left a final unidentified product from the tandem ChlE3/ChlL assay (*t*_R_ = 13.9), which we expected to be the pentacyclic product 29 of the [4 + 2]-cascade. Prior to attempting to purify this species, we screened enzyme assay conditions to direct the reaction towards this product. We found that carrying out the reaction with a two-fold excess of ChlE3 over ChlL gave the proposed pentacyclic product as the major species (Fig. 4B-v). The enzymatic reaction was scaled-up and the product was isolated by RP-HPLC. ^1^H, ^13^C and 2D NMR confirmed the expected connectivity of the pentacyclic scaffold 29, thus confirming the successful reconstitution of the proposed enzymatic [4 + 2]-cascade and hence the function of ChlL as a spirotetronate cyclase.

The stereochemistry of the spirotetronate motif could not be determined explicitly with the available data, although no NOE was observed between the OCH_3_ group and 21-methyl substituent, which is consistent with the expected *exo*-product with the (*S*)-configured spirocentre as is present in the final natural product chlorothricin. It is notable that the cascade proceeds successfully with the non-natural *O*-methyl tetronate analogue, as this further points to the potential utility of this system in chemoenzymatic synthesis, as these compounds are significantly more synthetically tractable than the corresponding free tetronic acids.

## Conclusion

In summary, we have revealed insights into Diels–Alderases from the chlorothricin biosynthetic pathway. To achieve this a flexible strategy for the selective synthesis of tetronates bearing unsaturated linear chains was developed. Using synthetic substrate 24, assembly of the pentacyclic core of the antibiotic chlorothricin was reconstituted *in vitro* and shown to be formed *via* sequential Diels–Alder reactions catalysed by the enzymes ChlE3 and ChlL. This has established a chemoenzymatic route to the chlorothricin core, which affords excellent control of product stereochemistry. The X-ray crystal structure of ChlE3 has been determined, providing molecular insights into the [4 + 2] cycloaddition reaction of this biocatalyst. *In vitro* enzyme assays demonstrate that both Diels–Alderases can accept non-natural substrates and gave access to a novel *trans*-decalin 28 with the creation of 2 rings and 4 new stereocentres as well as to macrocycle 30. Our findings provide both fundamental insights into biocatalytic pentacyclic core formation in class II spirotetronates and further demonstrate the utility of using naturally evolved Diels–Alderases to access complex natural product scaffolds *via* chemoenzymatic processes.

## Author contributions

M. A. H., P. R. R. and C. L. W. supervised the experimental work and acquired funding. Protein studies including over-expression and purification of the enzymes, protein crystallisation and X-ray crystallography was conducted by C. R. B., M. M.-R. and K. Z.; synthetic work including spectroscopy, bioassays and characterisation of products was carried out by A. J. D. The manuscript was prepared with input from all the authors, led by P. R. R. and C. L.W.

## Conflicts of interest

The authors declare there are no conflicts of interest.

## Supplementary Material

OB-024-D6OB00728G-s001

## Data Availability

The data supporting this article have been reported as part of the supplementary information (SI). Supplementary information: experimental procedures, analytical and spectral data, NMR spectra, protein expression, purification, crystallisation and X-ray. See DOI: https://doi.org/10.1039/d6ob00728g.
