# Supplementary material for: Creating molecular complexity in the chemoenzymatic synthesis of chlorothricin analogues using tandem Diels–Alderases
Source: Org Biomol Chem. 2026 Jun 17;24(26):5457–64. doi: 10.1039/d6ob00728g (PMC13273814; doi:10.1039/d6ob00728g)
Supplement: OB-024-D6OB00728G-s001 [file OB-024-D6OB00728G-s001.pdf]

Supplementary Information (SI) for Organic & Biomolecular Chemistry.
This journal is © The Royal Society of Chemistry 2026

Creating Molecular Complexity in the Chemoenzymatic Synthesis of Chlorothricin Analogues using Tandem Diels-Alderases

Andrew J. Devine,[+a] Monserrat Manzo-Ruiz,[+b,c], Catherine R. Back,[c] Katja Zorn,[d] Martin A. Hayes,[d,e] Paul R. Race,[b] Christine L. Willis[a]

[a] Dr. A. J. Devine and Prof. C. L. Willis
School of Chemistry, University of Bristol, Bristol, BS8 1TS (UK)
E-mail: chris.willis@bristol.ac.uk

[b] Dr. M. Manzo-Ruiz. and Prof. P. R. Race
School of Natural and Environmental Sciences, Newcastle University, Newcastle upon Tyne, NE1 7RU (UK)
E-mail: paul.race@bristol.ac.uk

[c] Dr. C. R. Back and Dr. M. Manzo-Ruiz
School of Biochemistry, University of Bristol, University Walk, Bristol, BS8 1TD (UK)

[d] Dr. K. Zorn and Prof. M. A. Hayes
BioPharmaceuticals R&D, AstraZeneca, Pepparedsleden 1, 43183, Mölndal, (Sweden)

[e] Prof. M. A. Hayes, School of Chemistry and Molecular Biosciences, The University of Queensland, St Lucia QLD 4067, Australia

Contents

1. Supplementary Methods 3
   - 1.1 Gene Cloning 3
   - 1.2 Protein Expression and Purification 3
   - 1.3 Protein Crystallisation and Diffraction Data Collection 4
   - 1.4 Structure Solution and Refinement 4
   - 1.5 [4+2]-Cyclase Assays 5
   - 1.6 LC-MS Analysis 5
   - 1.7 HPLC Purification 6
   - 1.8 Chemical Synthesis 6
   - 1.8.3 Synthetic Compounds 7
   - 1.9 Enzymatic Reaction Products 22
2. Supplementary Tables 24
3. Supplementary Figures 25
4. Molecular Modelling 27
5. Supplementary References 27
6. ^{1}H and ^{13}C NMR Spectra 29
   - 6.1 Synthetic Compounds 29
   - 6.2 Enzymatic Reaction Products 49
7. Amino Acid Sequences 60

1. Supplementary Methods

1.1 Gene Cloning

Codon optimised sequences of *chlE3* and *chlL* from *Streptomyces antibioticus* DSM 40725, for *E. coli* expression, were synthesised and cloned into pET-29b(+) between NdeI and XhoI restriction sites. Construct plasmids were sourced from Twist Bioscience (South San Francisco, CA).

1.2 Protein Expression and Purification

Both ChlE3 and ChlL were recombinantly expressed in *E. coli* BL21 (DE3). A single colony harbouring the transformed plasmid was used for over-night culture in 20 mL of LB broth supplemented with 50 µg/mL kanamycin, cells were grown at 37 °C, 180 rpm. 1% v/v final concentration of over-night culture was used for growth in LB broth at 37°C until an OD_{600} of 0.6-0.8 was reached, then 1 mM IPTG was added for induction. Cells were then incubated at 20°C for additional 16 hrs. 1 L and 3 L of bacterial cell culture were used to generate 20 mg/mL stocks of ChlE3 and ChlL, respectively. Cells were harvested by centrifugation at 4 400 x *g* for 30 min; pellet was washed once in load buffer (50 mM Tris-HCl, 150 mM NaCl, 20 mM Imidazole, pH 7.5) and flash frozen for storage at -80 °C until purification.

Thawed cells were resuspended in 35 mL of load buffer adding a tablet of Pierce™ protease inhibitor (Thermo Scientific), then disrupted in a cell press using 25 kpsi. Disrupted cells were centrifuged at 38 759 x *g* for 40 min at 4°C, supernatant was filtered in a Minisart™ 0.45 µm syringe filter (Sartorius). Both proteins were purified by immobilized metal affinity chromatography (IMAC) and size exclusion chromatography (SEC) using the ÄKTA purifier system (GE Healthcare).

After column calibration with 5 column volume (CV) of load buffer, filtered supernatant was loaded into a HisTrap™ HP 5 mL column (Cytiva) for IMAC purification and washed with 10 CV of load buffer. ChlE3 was eluted with increasing linear gradient of 20 to 500 mM imidazole for 45 mL into 1.5 mL fractions; for ChlL elution, linear gradient of 20 to 300 mM imidazole was used.

Eluted fractions were screened using SDS-PAGE, 4-20% Tris-Glycine (NuSep). The desired fractions were pooled and concentrated with Vivaspin® 20 10 kDa (Sartorius) up to 5 mL, sample was centrifuged for 5 min at 18 200 x g, 4 °C, and loaded onto a HiLoad 16/600 Superdex 200 pg column (GE Healthcare) for SEC using a buffer containing 20 mM Tris-HCl, 150 mM NaCl, pH 7.5. Fractions were screening for pure protein using SDS-PAGE gel and

the desired fractions were pooled and concentrated up to reach a concentration higher than 16 mg/mL.

A single aliquot of 500 μL of each pure protein was store at 4 °C for protein crystallisation, remanent volume was aliquoted in 50 μL samples, flash frozen and stored at –80 °C until protein activity determination.

1.3 Protein Crystallisation and Diffraction Data Collection

Conditions for ChlE3 crystallisation were stablished by sitting drop vapor diffusion method at 20 °C testing commercial crystallisation screens from Molecular Dimensions Ltd. Crystals of ChlE3 for diffraction were grown by hanging drop method using a crystallisation solution composed by 0.2 M Lithium chloride, 20% w/v PEG 6000, 0.1 M Tris, pH 8 (MDSR-36-D9, Molecular Dimensions Ltd), 1 mL of the crystallisation condition was dispensed into the reservoirs of a 24-well XRL plate (Molecular Dimensions Ltd) and drops were set adding 1 µL of 16 mg/mL ChlE3 and 1 µL of the crystallisation solution. Crystals grew after 1 week of incubation.

Selected crystals of ChlE3 were mounted in litholoops (Molecular Dimensions Ltd), then flash frozen in liquid nitrogen and stored in the cryogenic liquid until analysis.

X-ray diffraction data was collected at Diamond Light Source on beamline I24 by single wavelength anomalous diffraction (SAD). Auto-processed diffraction data by xia2 3dii, included in the Diamond Light Source Software Pipeline, was used for structure elucidation.

1.4 Structure Solution and Refinement

All diffraction data processing was undertaken using tools available in CCP4i2 software suite (8.0.019). Data scaling was performed with AIMLESS.[1] ChlE3 structure was solved based on the structure of PyrE3 (PDB 5XGV) by molecular replacement using PHASER.[2] Iterative manual model building and refinement were made in COOT[3] and Refmac5,[4] including TLS parameters in the later stages of refinement. Data collection, phasing and refinement statistics are provided in Table S2 for ChlE3. The structure of ChlE3 was deposited in the protein data bank (PDB) with the deposition code 9SRP. Pymol was used for structure visualisation.

1.5 [4+2]-Cyclase Assays

1.5.1 ChlE3 Activity Assay

In a total reaction volume of 100 µL (Tris buffer (50 mM Tris, 150mM NaCl), at pH 7.5, with 2% MeOH), **24** or **27** (0.5 mM) was incubated with ChlE3 (80 µM). After 2 h the reaction was

terminated by the addition of ice-cold MeCN (100 µL) and the reaction mixture clarified by centrifugation (14000 rpm, 4 min). The reaction mixture was then extracted with EtOAc (2 × 200 µL), and the organic extract dried under a stream of nitrogen. A control reaction was performed according to the same conditions, but without ChlE3.

1.5.2 ChlE3/ChlL Tandem Assay

In a total reaction volume of 100 µL (Tris buffer (50 mM Tris, 150mM NaCl), at pH 7.5, with 2% MeOH), **24** (0.5 mM) was incubated with ChlE3 (80 µM) and ChlL (40 µM). After 2 h the reaction was terminated by the addition of ice-cold MeCN (100 µL) and the reaction mixture clarified by centrifugation (14000 rpm, 4 min). The reaction mixture was then extracted with EtOAc (2 × 200 µL), and the organic extract dried under a stream of nitrogen. Two control reactions were performed according to the same conditions, but excluding either ChlL or both ChlL and ChlE3.

1.5.3 ChlL Activity Assay

In a total reaction volume of 100 µL (Tris buffer (50 mM Tris, 150mM NaCl), at pH 7.5, with 2% MeOH), **24** (0.5 mM) was incubated with ChlL (100 µM). After 2 h the reaction was terminated by the addition of ice-cold MeCN (100 µL) and the reaction mixture clarified by centrifugation (14000 rpm, 4 min). The reaction mixture was then extracted with EtOAc (2 × 200 µL), and the organic extract dried under a stream of nitrogen. A control reaction was performed according to the same conditions, but without ChlL.

1.6 LC-MS Analysis

Dried assays extracts were dissolved in MeCN (80 µL) and this solution was subjected to LC-MS analysis using a Waters 2445SFO HPLC system with a Waters 2298 diode array detector for UV between 200 and 400 nm. The system was equipped with a Phenomenex LUNA column (5 µm, C18, 100 Å, 4.6 × 250 mm) and elution was carried out with a linear gradient of 60-95% MeCN in H_2O with 0.05 % formic acid; flow rate: 1 mL/min; detection by a Waters 2424 evaporative light scattering detector (ELSD) system. Mass spectrometry was performed using a Waters QM ESI spectrometer in positive and negative modes, with detection between 150 and 1200 m/z units.

1.7 HPLC Purification

Assay products were purified by preparative reverse-phase HPLC using a Waters 2445SFO HPLC system with a Waters 2545 pump. The system was equipped with a Phenomenex Kinetex column (5 µ, C18, 100 Å, 250 x 21.20 mm) fitted with a Phenomenex Security Guard column (Luna C5 300 Å) and elution was carried out using a linear gradient of 60-95% MeCN

in H_2O with 0.05 % formic acid; flow rate: 16 mL/min. The post column solvent was split (100:1) and the minor flow was analysed by ELS (Waters 2424) and ESI mass spectrometry in positive and negative modes (Waters QM). The assay products were identified by ELS/MS collected using a Waters 2767 autosampler.

1.8 Chemical Synthesis

1.8.1 General Synthetic Methods

All reagents were obtained from commercial suppliers and used without further purification. All air and moisture sensitive reactions were carried out using standard Schlenk syringe-septa techniques, using flame dried glassware under a positive pressure of nitrogen. Anhydrous THF, MeCN, CH_2Cl_2, Et_2O, hexane and toluene were dried by passing through a modified Grubbs system of alumina columns, manufactured by Anhydrous Engineering, and stored over 3 Å molecular sieves. Anhydrous DMF was obtained from commercial suppliers and used without further drying. MeOH, TMP, DIPEA, and NEt_3 were distilled over CaH_2 prior to use. All stated temperatures below ambient are the temperatures of the cooling baths. TLC analysis was performed with aluminium backed silica TLC plates (Merck-Kieselgel 60 F_{254}) and the plates were visualised using UV fluorescence (254 nm) and/or developed with potassium permanganate solution. Flash column chromatography was performed according to the procedures described by Still,[5] using silica gel 60 (Fisher Scientific or Aldrich) and the stated solvent system.

1.8.2 Compound Characterisation

^{1}H, and ^{13}C spectra were recorded using Jeol ECZ 400, Jeol ECS 400, Bruker Nano 400, Bruker Avance III HD 500, Varian VNMRS500 and Bruker 700 spectrometers at ambient temperature. Chemical shifts (δ) are quoted in parts per million (ppm) and coupling constants (*J*) are in Hertz (Hz) rounded to 0.5 Hz intervals. Peaks were assigned as singlets (s), doublets (d), triplets (t), quartets (q) or a combination. When appropriate, peaks are also be described as apparent (app.) or broad (br.). Residual solvent peaks were used as the internal reference for proton and carbon chemical shifts ($CDCl_3$: 7.26 ppm for ^{1}H and 77.16 ppm for ^{13}C). Two-dimensional NMR techniques (^{1}H-^{1}H COSY, ^{1}H-^{13}C HSQC, ^{1}H-^{13}C HMBC) were used routinely for structural assignment. HRMS and MS analyses were performed by the Bristol Mass Spectrometry Service on either a Bruker Daltonics Apex 4, 7 Tesla FTICR or Bruker microTOF II. Samples were submitted in MeOH or CH_2Cl_2. Specific rotations ($[\alpha]_D^T$) were measured on a Bellingham and Stanley Ltd. ADP220 polarimeter and are quoted in (° ml) (g dm)$^{-1}$. Infra-red spectra were recorded neat as a thin film on a Perkin Elmer Spectrum 100 FTIR with an ATR accessory and selected frequencies are reported in wavenumbers (cm^{-1}).

1.8.3 Synthetic Compounds

Ethyl (2*E*,4*E*)-4-methylhexa-2,4-dienoate S2

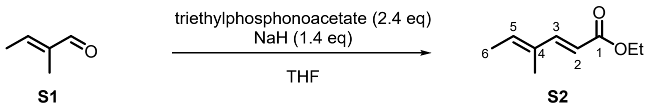

Under an atmosphere of nitrogen, NaH (60% suspension in mineral oil, 2.47 g, 61.8 mmol) was suspended in anhydrous THF (200 mL). The solution was cooled to 0 °C and triethylphosphonoacetate (17.0 mL, 85.7 mmol) was added dropwise. The solution was stirred at 0 °C temperature for 0.5 h and then tiglic aldehyde (2.30 mL, 23.8 mmol) was added dropwise. The reaction was allowed to warm to room temperature and stirred for 16 h. The reaction was quenched by the addition of sat. aq. NH_4Cl (50 mL) and the aqueous layer was extracted with EtOAc (3 × 80 mL). The combined organic layers were dried ($MgSO_4$), filtered and the solvent removed *in vacuo*. The crude residue was purified by column chromatography, eluting with 5% EtOAc in petroleum ether (40-60 °C) to give **S2** as a colourless oil (7.21 g, 98%). δ_H (400 MHz, $CDCl_3$) 7.29 (1H, d, *J* 15.5, 2-H), 5.95 (1H, q, *J* 7.0, 5-H), 5.75 (1H, d, *J* 15.5, 3-H), 4.18 (2H, q, *J* 7.0, $CO_2CH_2CH_3$), 1.78 (3H, d, *J* 7.0, 6-H_3), 1.74 (3H, s, 4-CH_3), 1.27 (3H, t, *J* 7.0, $CO_2CH_2CH_3$). δ_C (101 MHz, $CDCl_3$) 167.7 (C-1), 149.6 (C-2), 136.4 (C-5), 133.8 (C-4), 115.3 (C-3), 60.2 ($CO_2CH_2CH_3$), 14.6 (C-6), 14.4 ($CO_2CH_2CH_3$), 11.8 (4-CH_3). **MS** (APCI+) calc. for $[C_9H_{14}O_2+H]^+$155.1, found 155.1. **IR** (ν_{max}/cm^{-1}) 2982, 1707, 1620.

All data in accordance with the literature.[6]

(2*E*,4*E*)-4-Methylhexa-2,4-dien-1-ol S3

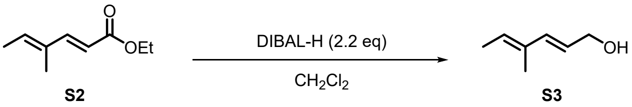

Under an atmosphere of nitrogen, ester **S2** (7.21 g, 46.8 mmol) was dissolved in CH_2Cl_2 (160 mL) and the solution cooled to –78 °C. DIBAL-H (1.0 M in hexanes, 103 mL, 103 mmol) was added dropwise and the reaction was allowed to warm to room temperature and stirred for 1.5 h. The reaction was cooled to 0 °C and sat. aq. potassium sodium tartrate (150 mL) was added. The resulting cloudy suspension was stirred vigorously until biphasic and the aqueous layer extracted with CH_2Cl_2 (3 × 100 mL). The combined organic layers were dried ($MgSO_4$), filtered and the solvent removed *in vacuo* to give **S3** as a colourless oil (4.75 g, 91%). δ_H (400 MHz, $CDCl_3$) 6.23 (1H, d, *J* 15.5, 3-H), 5.68 (1H, dt, *J* 15.5, 6.0, 2-H), 5.55 (1H, q, *J* 6.5, 5-H), 4.16 (2H, d, *J* 6.0, 1-H_2), 1.92 (1H, br s, OH) 1.72 (3H, overlapping s, 4-CH_3), 1.71 (3H, overlapping d, *J* 6.5, 6-H_3). δ_C (101 MHz, $CDCl_3$) 136.7 (C-2), 133.9 (C-4), 127.5 (C-5), 124.9

(C-3), 64.0 (C-1), 13.9 (C-6), 12.1 (4-CH_3). **MS** (APCI+) calc. for $[C_7H_{12}O\text{-}H_2O]^+$ 95.1, found 95.1. **IR** (v_{max}/cm^{-1}) 3320, 2918, 2860.

All data in accordance with the literature.[6]

Diethyl ((2*E*,4*E*)-4-methylhexa-2,4-dien-1-yl)phosphonate 8

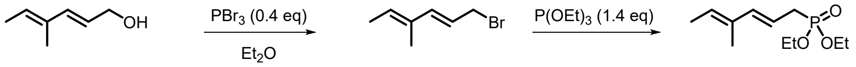

S3 **S4** **8**

Under an atmosphere of nitrogen, alcohol **S3** (3.85 g, 34.3 mmol) was dissolved in Et_2O (250 mL) and the solution was cooled to 0 °C. PBr_3 (1.30 mL, 13.73 mmol) was added dropwise and the reaction was stirred at 0 °C for 16 h. The reaction was quenched by the addition of H_2O (200 mL) and the aqueous layer was extracted with Et_2O (4 × 100 mL). The combined organic layers were washed with brine (200 mL) and sat. aq. $NaHCO_3$ (200 mL), dried ($MgSO_4$), filtered and the solvent removed *in vacuo* to give crude **S4** as a yellow oil which was used immediately without further purification.

Bromide **S4** was added dropwise to triethyl phosphite (9.76 mL, 57.0 mmol) and the reaction was heated at 160 °C for 16 h. The reaction was cooled to room temperature and fitted with a distillation apparatus. Excess triethyl phosphite was removed by distillation of the crude mixture at 200 °C. The crude residue was purified by column chromatography, eluting with 80% EtOAc in petroleum ether (40-60 °C) to give **8** as a light-yellow oil (3.54 g, 44% over two steps). **δ_H** (400 MHz, $CDCl_3$) 6.15 (1H, dd, *J* 15.5, 5.0, 3-H), 5.45 (2H, overlapping m, 2-H and 5-H), 4.06 (4H, m, OCH_2CH_3), 2.61 (2H, dd, *J* 22.0, 7.5, 1-H_2), 1.70 (3H, overlapping s, 4-CH_3), 1.68 (3H, overlapping d, *J* 7.5, 6-H_3) 1.28 (6H, t, *J* 7.0, OCH_2CH_3). **δ_C** (126 MHz, $CDCl_3$) 139.8 (d, *J* 15.0, C-3), 134.1(d, *J* 4.5, C-4), 126.8 (d, *J* 4.0, C-5), 114.7 (d, *J* 12.0, C-2), 62.0 (d, *J* 7.0, OCH_2CH_3), 30.8 (d, *J* 140, C-1), 16.6 (d, *J*, 6.0, OCH_2CH_3) 13.9 (C-6), 12.1 (4-CH_3). **δ_P** (162 MHz, $CDCl_3$) 28.33**.** **HRMS** (ESI) calc. for $[C_{11}H_{21}O_3P\text{+}Na]^+$ 255.1126, found 255.1124.

All data in accordance with the literature.[6]

(*E*)-7-(4′,4′,5′,5′-Tetramethyl-1′,3′,2′-dioxaborolan-2′-yl)hept-6-en-1-ol 9

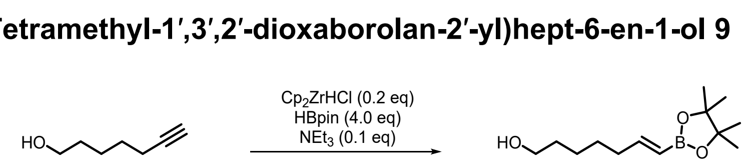

S5 **9**

Adapting a procedure reported by Wang, [7] under an atmosphere of nitrogen, HBpin (4.35 g, 34.0 mmol), NEt_3 (135 µL, 0.972 mmol) and Cp_2ZrHCl (753 mg, 2.92 mmol) were sequentially added to stirred **S5** (1.09 g, 9.72 mmol) and the reaction mixture was heated at 40 °C for 16

h. The reaction mixture was cooled to room temperature, quenched by the addition of H_2O (10 mL) and diluted with CH_2Cl_2 (20 mL). The aqueous layer was extracted with CH_2Cl_2 (3 × 20 mL). The organic layers were combined, dried ($MgSO_4$), filtered and the solvent removed *in vacuo.* The crude residue was purified by column chromatography, eluting with 30% EtOAc in petroleum ether (40-60 °C) to give **9** as a colourless oil (1.82 g, 78%). **δ_H** (400 MHz, $CDCl_3$) 6.62 (1H, dt, 18.0, 6.5, 6-H), 5.40 (1H, dt, 18.0, 1.5, 7-H), 3.62 (2H, t, *J* 6.5, 1-H_2), 2.16 (2H, dtd, 8.0, 6.5, 1.5, 5-H_2), 1.57 (2H, m, 2-H_2), 1.47 – 1.31 (4H, overlapping m, 3-H_2 and 4-H_2), 1.25 (12H, Bpin CH_3 × 4). **δ_C** (101 MHz, $CDCl_3$) 154.4 (C-6), 83.0 (Bpin *C*(CH_3)$_2$), 62.9 (C-1), 35.7 (C-5), 32.6 (C-2), 28.0 (C-4), 25.3 (C-3), 24.8 (Bpin C(CH_3)$_2$). NB C-7 not observed due to ^1J(C,B) coupling. **HRMS** (ESI) calc. for $[C_{13}H_{25}O_3B+Na]^+$ 263.1794, found 263.1796. **IR** (ν_{max}/cm^{-1}) 3309, 2933, 2861, 1637.

Methyl 5-oxo-7-(trimethylsilyl)hept-6-ynoate 13

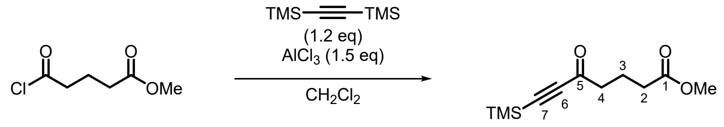

12 13

Under an atmosphere of nitrogen, $AlCl_3$ (14.6 g, 109 mmol) was suspended in CH_2Cl_2 (80 mL), and the suspension cooled to 0 °C. A solution of glutaric acid monomethyl ester chloride (10.1 mL, 73.0 mmol) and bistrimethylsilylacetylene (14.9 g, 87.5 mmol) in CH_2Cl_2 (100 mL) was added to the $AlCl_3$ suspension. The reaction was stirred at 0 °C for 45 minutes and then quenched by the slow addition of ice cold sat. aq. citric acid (80 mL). The aqueous layer was extracted with CH_2Cl_2 (2 × 100 mL), the organic layers combined, washed with brine (100 mL), dried ($MgSO_4$), filtered and the solvent removed *in vacuo*. The crude residue was purified by column chromatography eluting with 20% EtOAc in petroleum ether (40-60 °C) to give keto ester **13** as a yellow oil (13.2 g, 80%). **δ_H** (400 MHz, $CDCl_3$) 3.67 (3H, s, CO_2CH_3), 2.64 (2H, t, *J* 7.0, 4-H_2), 2.36 (2H, t, *J* 7.5, 2-H_2), 1.96 (2H, app. t, *J* 7.0, 3-H_2), 0.23 (9H, s, Si(CH_3)$_3$). **δ_C** (101 MHz, $CDCl_3$) 186.8 (C-5), 173.4 (C-1), 101.9 (C-6), 98.2 (C-7), 51.7 (CO_2*C*H_3), 44.3 (C-4), 32.9 (C-2), 19.1 (C-3), –0.7 (Si(CH_3)$_3$). **MS** (ESI) calc. for $[C_{11}H_{18}O_3Si+Na]^+$ 249.09, found 249.10. **IR** (ν_{max}/cm^{-1}) 2957, 2150, 1737, 1676.

All data in accordance with the literature.[8]

Methyl (*S*)-5-hydroxy-7-(trimethylsilyl)hept-6-ynoate 14

Synthesis of (*S*,*S*)-Noyori Catalyst S7

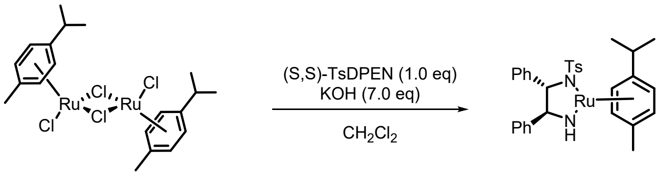

S6 (0.5 eq) **S7**

Adapting a procedure reported by Thomson,[9] under an atmosphere of nitrogen, (*S,S*)-TsDPEN (337 mg, 0.92 mmol) and [$RuCl_2$(η[6]-*p*-cymene)]$_2$ (282 mg, 0.46 mmol) were added to CH_2Cl_2 (15 mL). Powdered KOH (361 mg, 6.44 mmol) was added, and the orange solution was stirred for 5 minutes at room temperature. H_2O (15 mL) was added and the biphasic mixture was stirred for 10 minutes. The mixture was diluted with H_2O (30 mL) and the aqueous layer was extracted with CH_2Cl_2 (2 × 20 mL). The combined organic layers were dried (CaH_2), filtered and the solvent removed *in vacuo* to give the catalyst **S7** (524 mg, 95%) as a dark purple film that was used immediately without further purification.

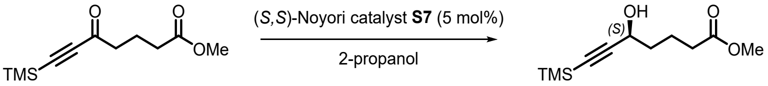

13 **14**

Under an atmosphere of nitrogen, **13** (4.00 g, 17.7 mmol) was dissolved in degassed 2-propanol (150 mL). (*S*,*S*)-Noyori catalyst **S7** (524 mg, 0.87 mmol) in CH_2Cl_2 (5 mL) was added and the reaction was stirred at room temperature for 1.5 h. The solvent was removed *in vacuo* and the crude residue was purified by column chromatography, eluting with 25% EtOAc in petroleum ether (40-60 °C) to give alcohol **14** as a colourless oil (3.72 g, 92% yield). ^{1}H NMR analysis of the (S)-Mosher's ester derivative **S8** indicated an enantiomeric excess of 96%. $[\alpha]^{22}_{D}$ = –3.0 (*c* 1.0, $CHCl_3$) (Lit. $[\alpha]^{24}_{D}$ = –1.1 (*c* 5.5, $CHCl_3$)).[8] **δ_H** (400 MHz, $CDCl_3$) 4.36 (1H, t, *J* 6.0, 5-H), 3.66 (3H, s, CO_2CH_3), 2.37 (2H, t, *J* 7.0, 2-H_2), 2.11 (1H, br s, OH), 1.88 – 1.63 (4H, overlapping m, 3-H_2 and 4-H_2), 0.15 (9H, s, $Si(CH_3)_3$). **δ_C** (101 MHz, $CDCl_3$) 174.1 (C-1), 106.5 (C-6), 89.7 (C-7), 62.5 (C-5), 51.7 (CO_2CH_3), 37.0 (C-4), 33.7 (C-2), 20.7 (C-3), –0.1 ($Si(CH_3)_3$). **MS** (ESI) calc. for $[C_{11}H_{20}O_3Si+Na]^+$ 251.1, found 251.1. **IR** (v_{max}/cm^{-1}) 3446, 2954, 2171, 1740.

All data in accordance with the literature.[8]

Synthesis of Mosher's ester S8 for *ee* determination

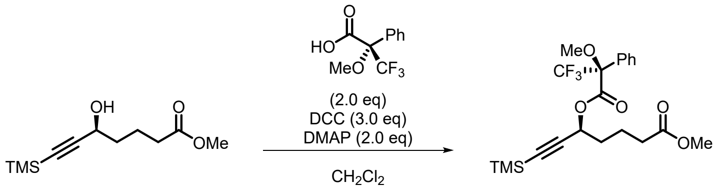

14 **S8**

Alcohol **14** (17 mg, 0.073 mmol) was dissolved in CH_2Cl_2 (1 mL) and the solution cooled to 0 °C. (*S*)-(−)-α-Methoxy-α-(trifluoromethyl)phenylacetic acid (34 mg, 0.145 mmol), DCC (45 mg, 0.219 mmol) and DMAP (18 mg, 0.145 mmol) were then added sequentially and the solution stirred at room temperature for 16 h. The reaction mixture was filtered through a pad of silica, the pad washed with CH_2Cl_2 (2 × 10 mL) and the filtrate concentrated *in vacuo* to give ester **S8** as a colourless oil (30 mg, 94%). The diastereomeric ratio of the product was established by ^{1}H NMR analysis.

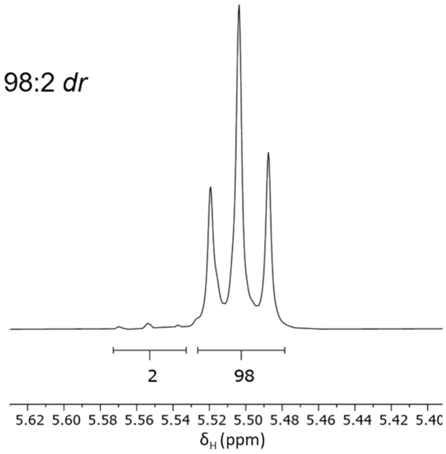

Methyl (*S*)-5-((*tert*-butyldimethylsilyl)oxy)-7-(trimethylsilyl)hept-6-ynoate 15

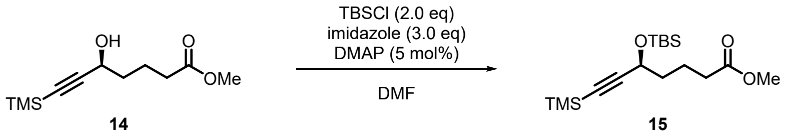

14 **15**

Under an atmosphere of nitrogen, alcohol **14** (3.13 g, 13.7 mmol) was dissolved in DMF (65 mL) and the solution cooled to 0 °C. TBSCl (4.13 g, 27.4 mmol), imidazole (2.80 g, 41.1 mmol) and DMAP (84 mg, 0.69 mmol) were added and the solution was allowed to warm to room temperature and stirred for 2 h. The reaction was quenched by the addition of sat. aq. NH_4Cl (5 mL) and the aqueous layer extracted with Et_2O (3 × 20 mL). The combined organic layers were washed with H_2O (2 × 20 mL) and brine (20 mL), dried ($MgSO_4$), filtered and the solvent removed *in vacuo*. The crude residue was purified by column chromatography, eluting with 5% EtOAc in petroleum ether (40-60 °C) to give **15** as a colourless oil (4.46 g, 95%). $[\alpha]_D^{22}$ = –36.0 (*c* 1.0, $CHCl_3$) (Lit. $[\alpha]_D^{20}$ = –42.2 (*c* 3.5, $CHCl_3$)).[8] **δ_H** (400 MHz, $CDCl_3$) 4.34 (1H, t, *J* 6.0, 5-H), 3.66 (3H, s, CO_2CH_3), 2.34 (2H, t, *J* 7.0, 2-H_2), 1.85 – 1.46 (4H, overlapping m, 3-H_2 and 4-H_2), 0.89 (9H, s, $SiC(CH_3)_3$), 0.14 (9H, s, $Si(CH_3)_3$), 0.12 (3H, s, $Si(CH_3)_2$), 0.10 (3H, s, $Si(CH_3)_2$). **δ_C** (101 MHz, $CDCl_3$) 174.1 (C-1), 107.5 (C-6), 88.9 (C-7), 63.1 (C-5), 51.6 (CO_2*C*H_3), 37.8 (C-4), 33.8 (C-2), 25.9 (SiC(*C*$H_3)_3$), 20.9 (C-3), 18.4 (Si*C*$(CH_3)_3$), –0.05

$(Si(CH_3)_3)$, –4.3 $(Si(CH_3)_2)$, –4.8 $(Si(CH_3)_2)$. **MS** (ESI) calc. for $[C_{17}H_{34}O_3Si_2+Na]^+$ 365.2, found 365.2. **IR** (ν_{max}/cm^{-1}) 2956, 2171, 1743.

All data in accordance with the literature.[8]

(*S*)-5-((*tert*-Butyldimethylsilyl)oxy)-7-(trimethylsilyl)hept-6-ynal 16

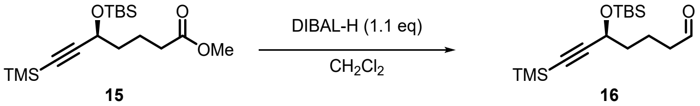

Under an atmosphere of nitrogen, **15** (9.04 g, 26.4 mmol) was dissolved in CH_2Cl_2 (200 mL) and the solution was cooled to –78 °C. DIBAL-H (1.0 M in hexanes, 29.0 mmol, 29.0 mL) was added dropwise and the solution was stirred for 1.5 h at the same temperature. The reaction was quenched at –78 °C by the addition of MeOH (3 mL), followed by sat. aq. potassium sodium tartrate (100 mL). The mixture was allowed to warm to room temperature and the resulting cloudy suspension was stirred vigorously until biphasic. The aqueous layer was extracted with CH_2Cl_2 (3 × 80 mL), the organic layers combined, dried ($MgSO_4$), filtered and the solvent removed *in vacuo.* The crude residue was purified by column chromatography, eluting with 5% EtOAc in petroleum ether (40-60 °C) to give **16** as a colourless oil (6.99 g, 85%). $[\alpha]_D^{22}$ = –12.0 (*c* 1.0, $CHCl_3$). **δ_H** (400 MHz, $CDCl_3$) 9.76 (1H, t, *J* 2.0, 1-H), 4.36 (1H, t, *J* 6.0, 5-H), 2.47 (2H, td, *J* 7.0, 2.0, 2-H_2), 1.83 – 1.65 (4H, overlapping m, 3-H_2 and 4-H_2), 0.90 (9H, s, $SiC(CH_3)_3$), 0.15 (9H, s, $Si(CH_3)_3$), 0.13 (3H, s, $Si(CH_3)_2$), 0.11 (3H, s, $Si(CH_3)_2$). **δ_C** (101 MHz, $CDCl_3$) 202.5 (C-1), 107.3 (C-6), 89.2 (C-7), 63.1 (C-5), 43.6 (C-2), 37.8 (C-4), 26.0 $(SiC(\mathit{C}H_3)_3)$, 18.4 $(Si\mathit{C}(CH_3)_3)$, 18.1(C-3), –0.1 $(Si(CH_3)_3)$, –4.3 $(Si(CH_3)_2)$, –4.8 $(Si(CH_3)_2)$. **HRMS** (APCI+) calc. for $[C_{16}H_{32}O_2Si_2-H]^+$ 311.1857, found 311.1859. **IR** (ν_{max}/cm^{-1}) 2955, 2857, 2172, 1729.

Ethyl (*S,E*)-7-((*tert*-butyldimethylsilyl)oxy)-2-methyl-9-(trimethylsilyl)non-2-en-8-ynoate 17

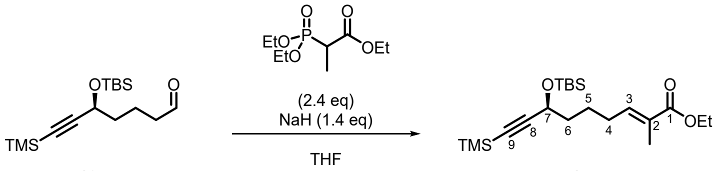

Under an atmosphere of nitrogen, NaH (60% suspension in mineral oil, 1.10 g, 27.4 mmol) was suspended in anhydrous THF (190 mL). The suspension was cooled to 0 °C and triethyl 2-phosphonopropionate (10.1 mL, 47.0 mmol) was added dropwise. The reaction was stirred at 0 °C for 0.5 h and then **16** (6.12 g, 19.6 mmol) in anhydrous THF (20 mL) was added dropwise. The reaction was allowed to warm to room temperature and stirred for 1.5 h. The

reaction was quenched by the addition of sat. aq. NH_4Cl (20 mL) and the aqueous layer was extracted with EtOAc (3 × 30 mL). The combined organic layers were dried ($MgSO_4$), filtered and the solvent removed *in vacuo*. The crude residue was purified by column chromatography, eluting with 5% EtOAc in petroleum ether (40-60 °C) to give **17** as a colourless oil (6.35 g, 82%). ^{1}H NMR showed a 2,3 *E*:*Z* ratio of 5:1. $[\alpha]_D^{22}$ = –20.0 (*c* 1.0, $CHCl_3$). **δ_H** (500 MHz, $CDCl_3$) 6.75 (1H, td, *J* 7.5, 1.5, 3-H), 4.34 (1H, dd, *J* 7.0, 5.5, 7-H), 4.19 (2H, q, *J* 7.0, $CO_2CH_2CH_3$), 2.20 (2H, m, 4-H_2), 1.83 (3H, d, *J* 1.5, 2-CH_3), 1.71 – 1.52 (4H, overlapping m, 5-H_2 and 6-H_2), 1.30 (3H, t, *J* 7.0, $CO_2CH_2CH_3$), 0.90 (9H, s, $SiC(CH_3)_3$), 0.15 (9H, s, $Si(CH_3)_3$), 0.13 (3H, s, $Si(CH_3)_2$), 0.11 (3H, s, $Si(CH_3)_2$). **δ_C** (126 MHz, $CDCl_3$) 168.4 (C-1), 142.0 (C-3), 128.2 (C-2), 107.7 (C-8), 88.9 (C-9), 63.3 (C-7), 60.6 ($CO_2CH_2CH_3$), 38.2 (C-6), 28.5 (C-4), 26.0 ($SiC(CH_3)_3$), 24.5 (C-5), 18.4 ($SiC(CH_3)_3$), 14.5 ($CO_2CH_2CH_3$), 12.6 (2-CH_3), 0.0 ($Si(CH_3)_3$), –4.3 ($Si(CH_3)_2$), –4.8 ($Si(CH_3)_2$). **HRMS** (ESI) calc. for $[C_{21}H_{40}O_3Si_2+Na]^+$ 419.2414, found 419.2415 **IR** (ν_{max}/cm^{-1}) 2956, 2858, 2172, 1712.

Methyl (*S*,*E*)-7-((*tert*-butyldimethylsilyl)oxy)-2-methylnon-2-en-8-ynoate 18

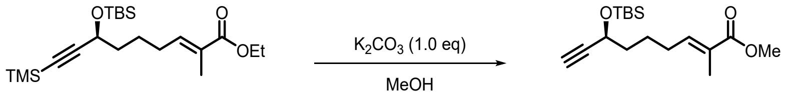

17 **18**

Under an atmosphere of nitrogen, **17** (1.81 g, 4.56 mmol) was dissolved in MeOH (46 mL), K_2CO_3 (630 mg, 4.56 mmol) was added and the solution was stirred at room temperature for 16 h. The solvent was removed *in vacuo*, the residue was partitioned between H_2O (30 mL) and EtOAc (30 mL) and the aqueous layer was extracted with EtOAc (3 × 30 mL). The combined organic layers were dried ($MgSO_4$), filtered and the solvent removed *in vacuo*. The crude residue was purified by column chromatography, eluting with 10% EtOAc in petroleum ether (40-60 °C) to give alkyne **18** as a colourless oil (1.35 g, 95%). ^{1}H NMR showed a 2,3 *E*:*Z* ratio of 5:1. $[\alpha]_D^{22}$ = –28.0 (*c* 1.0, $CHCl_3$). **δ_H** (400 MHz, $CDCl_3$) 6.75 (1H, m, 3-H), 4.35 (1H, ddd, *J* 6.5, 6.0, 2.0, 7-H), 3.73 (3H, s, CO_2CH_3), 2.37 (1H, d, *J* 2.0, 9-H), 2.20 (2H, m, 4-H_2), 1.83 (3H, d, *J* 1.5, 2-CH_3), 1.69 (2H, m, 6-H_2) 1.64 – 1.46 (2H, m, 5-H_2), 0.90 (9H, s, $SiC(CH_3)_3$), 0.13 (3H, s, $Si(CH_3)_2$), 0.10 (3H, s, $Si(CH_3)_2$). **δ_C** (101 MHz, $CDCl_3$) 168.8 (C-1), 142.2 (C-3), 128.0 (C-2), 85.5 (C-8), 72.4 (C-9), 62.6 (C-7), 51.8 (CO_2CH_3), 38.2 (C-6), 28.4 (C-4), 25.9 ($SiC(CH_3)_3$), 24.3 (C-5), 18.3 ($SiC(CH_3)_3$), 12.6 (2-CH_3), –4.4 ($Si(CH_3)_2$), –4.9 ($Si(CH_3)_2$). **HRMS** (ESI) calc. for $[C_{17}H_{30}O_3Si+Na]^+$ 333.1862, found 333.1862 **IR** (ν_{max}/cm^{-1}) 3310, 2951, 2858, 1715. NB Alkyne deprotection is complete after approximately 1 h, 16 h reaction time is required to avoid obtaining the product as a mixture of methyl and ethyl esters.

Methyl (*S*,2*E*,8*E*)-7-((*tert*-butyldimethylsilyl)oxy)-9-iodo-2-methylnona-2,8-dienoate 10

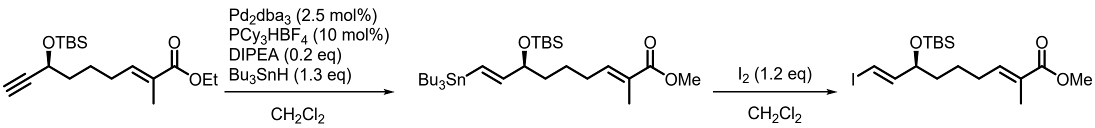

18 **19** **10**

Adapting a procedure reported by Darwish,[10]under an atmosphere of nitrogen, Pd_2dba_3 (208 mg, 0.227 mmol), PCy_3HBF_4 (334 mg, 0.908 mmol) and DIPEA (316 μL, 1.816 mmol) were added to degassed CH_2Cl_2 (60 mL) and the solution stirred at room temperature for 15 minutes. Alkyne **18** (2.82 g, 9.08 mmol) in degassed CH_2Cl_2 (10 mL) was added, and the solution was cooled to 0 °C. Bu_3SnH (3.18 mL, 11.80 mmol) in degassed CH_2Cl_2 (30 mL) was added dropwise and the solution was stirred for 2 h at 0 °C. The solvent was removed *in vacuo* and the crude residue was purified by column chromatography, eluting with 2% Et_2O in petroleum ether (40-60 °C) to give vinyl stannane **19** which was used immediately in the following step. **19** was dissolved in CH_2Cl_2 (60 mL) and the solution cooled to 0 °C. I_2 (2.76 g, 10.90 mmol) in CH_2Cl_2 was added and the solution was stirred at 0 °C for 15 minutes. The reaction was quenched by the addition of sat. aq. $Na_2S_2O_3$ (40 mL), diluted with CH_2Cl_2 (40 mL) and the aqueous layer extracted with CH_2Cl_2 (2 × 50 mL). The combined organic layers were washed with aq. KF (1 M, 3 × 40 mL), dried ($MgSO_4$), filtered and the solvent removed *in vacuo*. The crude residue was purified by column chromatography eluting with 2% Et_2O in petroleum ether (40-60 °C) to give vinyl iodide **10** a colourless oil (3.61 g, 90% over two steps). $[\alpha]_D^{23}$= –16.0 (*c* 1.0, $CHCl_3$). **δ_H** (400 MHz, $CDCl_3$) 6.74 (1H, m, 3-H), 6.50 (1H, dd, *J* 14.5, 6.0, 8-H), 6.21 (1H, dd, *J* 14.5, 1.0, 9-H), 4.09 (1H, m, 7-H), 3.73 (3H, s, CO_2CH_3), 2.17 (2H, m, 4-H_2), 1.82 (3H, d, *J* 1.5, 2-CH_3), 1.41 – 1.46 (4H, overlapping m, 5-H_2 and 6-H_2), 0.88 (9H, s, $SiC(CH_3)_3$), 0.04 (3H, s, $Si(CH_3)_2$), 0.03 (3H, s, $Si(CH_3)_2$). **δ_C** (101 MHz, $CDCl_3$) 168.8 (C-1), 149.1 (C-8), 142.2 (C-3), 128.0 (C-2), 76.0 (C-9), 75.1 (C-7), 51.9 (CO_2CH_3), 37.2 (C-6), 28.7 (C-4), 26.0 ($SiC(CH_3)_3$), 24.0 (C-5), 18.3 (Si*C*($CH_3)_3$), 12.6 (2-CH_3), –4.4 ($Si(CH_3)_2$), –4.7 ($Si(CH_3)_2$). **HRMS** (ESI) calc. for $[C_{17}H_{31}IO_3Si+Na]^+$ 461.0985, found 461.0000 **IR** (v_{max}/cm^{-1}) 2950, 2856, 1714.

Methyl (*S*,2*E*,8*E*,10*E*)-7-((*tert*-butyldimethylsilyl)oxy)-16-hydroxy-2-methylhexadeca - 2,8,10-trienoate 20

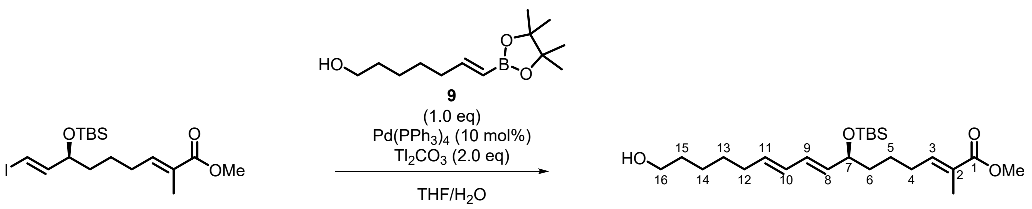

10 **20**

Under an atmosphere of nitrogen, **9** (101 mg, 0.420 mmol) was dissolved in a mixture of degassed THF (4 mL) and degassed H_2O (2 mL). Vinyl iodide **10** (184 mg, 0.420 mmol) in degassed THF (2 mL) and $Pd(PPh_3)_4$ (49 mg, 0.042 mmol) were added, and the reaction was stirred at room temperature for 15 minutes. Tl_2CO_3 (394 mg, 0.840 mmol) was added, and the reaction was heated at 50 °C for 16 h. The reaction was cooled to room temperature and filtered through a pad of celite, washing with Et_2O (20 mL) and H_2O (10 mL). The layers were separated, and the aqueous layer extracted with Et_2O (3 × 20 mL). The combined organic layers were dried ($MgSO_4$), filtered and the solvent removed *in vacuo*. The crude residue was purified by column chromatography eluting with 30% EtOAc in petroleum ether (40-60 °C) to give **20** as a colourless oil (159 mg, 89%). $[\alpha]^{23}_{D}$= +8.0 (*c* 1.0, $CHCl_3$). **δ_H** (400 MHz, $CDCl_3$) 6.74 (1H, ddd, *J* 9.0, 7.0, 1.5, 3-H), 6.09 – 5.96 (2H, overlapping m, 9-H and 10-H), 5.63 (1H, dt, *J* 14.0, 7.0, 11-H), 5.49 (1H, dd, *J* 14.5, 6.0, 8-H), 4.10 (1H, app. q, *J* 7.0, 7-H), 3.72 (3H, s, CO_2CH_3), 3.64 (2H, t, *J* 6.5 16-H_2), 2.15 (2H, m, 4-H_2), 2.09 (2H, m, 12-H_2), 1.81 (3H, s, 2-CH_3), 1.63 – 1.32 (10H, overlapping m, 5-H_2, 6-H_2, 13-H_2, 14-H_2 and 15-H_2), 0.88 (9H, s, $SiC(CH_3)_3$), 0.03 (3H, s, $Si(CH_3)_2$), 0.01 (3H, s, $Si(CH_3)_2$). **δ_C** (101 MHz, $CDCl_3$) 168.9 (C-1), 142.7 (C-3), 134.5 (C-8), 134.3 (C-11), 130.0 (C-9), 129.8 (C-10), 127.7 (C-2), 73.2 (C-7), 63.1 (C-16), 51.8 ($CO_2\mathit{C}H_3$), 38.2 (C-6), 32.8 (C-12), 32.7 (C-15), 29.2 (C-13), 28.8 (C-4), 26.0 ($SiC(\mathit{C}H_3)_3$), 25.5 (C-14), 24.4 (C-5), 18.4 ($Si\mathit{C}(CH_3)_3$), 12.5 (2-CH_3), –4.1 ($Si(CH_3)_2$), –4.7 ($Si(CH_3)_2$). **HRMS** (ESI) calc. for $[C_{24}H_{44}O_4Si+Na]^+$ 447.2907, found 447.2903 **IR** (v_{max}/cm^{-1}) 3416, 2930, 2857, 1716.

Methyl (*S*,2*E*,8*E*,10*E*)-7-((*tert*-butyldimethylsilyl)oxy)-2-methyl-16-oxohexadeca-2,8,10-trienoate 21

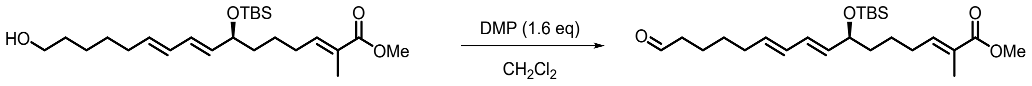

20 21

Under an atmosphere of nitrogen, **20** (123 mg, 0.290 mmol) was dissolved in CH_2Cl_2 (3 mL). The solution was cooled to 0 °C and DMP (196 mg, 0.464 mmol) was added. The reaction was allowed to warm to room temperature and stirred for 1 h. The reaction was quenched by the addition of sat. aq. $Na_2S_2O_3$ (5 mL) and sat. aq. $NaHCO_3$ (1 mL). The aqueous layer was extracted with CH_2Cl_2 (3 × 10 mL) and the combined organic layers were washed with sat. aq. $NaHCO_3$ (2 × 10 mL), dried ($MgSO_4$), filtered and the solvent removed *in vacuo*. The crude residue was purified by column chromatography, eluting with 10% EtOAc in petroleum ether (40-60 °C) to give **21** as a colourless oil (101 mg, 82%). **δ_H** (400 MHz, $CDCl_3$) 9.76 (1H, t, *J* 2.0, 16-H), 6.74 (1H, td, *J* 7.5, 1.5, 3-H), 6.16 – 5.90 (2H, overlapping m, 9-H and 10-H), 5.61 (1H, m, 11-H), 5.49 (1H, dt, *J* 14.5, 6.5, 8-H), 4.10 (1H, m, 7-H), 3.73 (3H, s, CO_2CH_3), 2.43

(2H, td, *J* 7.5, 2.0, 15-H_2), 2.16 (2H, app. q, *J* 7.0, 4-H_2), 2.09 (2H, m, 12-H_2), 1.82 (3H, s, 2-CH_3), 1.64 (2H. app. p, *J* 7.5, 14-H_2), 1.55 – 1.39 (6H, overlapping m, 5-H_2, 6-H_2 and 13-H_2), 0.88 (9H, s, SiC(CH_3)$_3$), 0.04 (3H, s, Si(CH_3)$_2$), 0.01 (3H, s, Si(CH_3)$_2$). **δ_C** (126 MHz, $CDCl_3$) 202.8 (C-16), 168.9 (C-1), 142.7 (C-3), 134.9 (C-8), 133.6 (C-11), 130.4 (C-10), 129.7 (C-9), 127.7 (C-2), 73.2 (C-7), 51.8 (CO_2*C*H_3), 43.9 (C-15), 38.2 (C-6), 32.4 (C-12), 28.86 (C-13), 28.80 (C-4), 26.0 (SiC(*C*H_3)$_3$), 24.4 (C-5), 21.8 (C-14), 18.4 (Si*C*(CH_3)$_3$), 12.4 (2-CH_3), –4.1(Si(CH_3)$_2$), –4.6 (Si(CH_3)$_2$). **HRMS** (ESI) calc. for $[C_{24}H_{42}O_4Si+Na]^+$ 445.2750, found 445.2754. **IR** (ν_{max}/cm^{-1}) 2953, 2865, 1716.

Methyl (*S*,2*E*,8*E*,10*E*,16*E*,18*E*,20*E*)-7-((*tert*-butyldimethylsilyl)oxy)-2,20-dimethyldocosa-2,8,10,16,18,20-hexaenoate 22

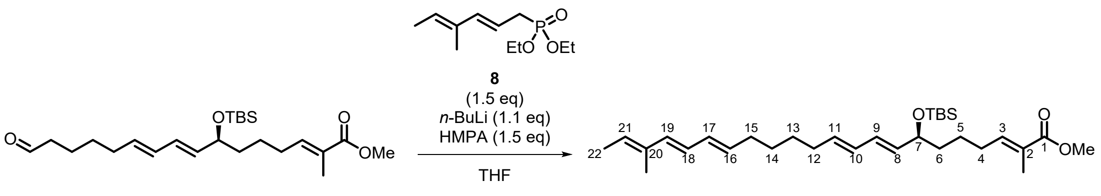

Adapting a procedure reported by Thomas,[11]under an atmosphere of nitrogen, phosphonate **8** (262 mg, 1.13 mmol) was dissolved in anhydrous THF (4 mL). The solution was cooled to –78 °C and *n*-BuLi (1.11 M in hexane, 748 µL, 0.830 mmol) was added dropwise. The reaction was stirred for 50 minutes at –78 °C and then the reaction flask was raised out of the cooling bath and stirred at room temperature for 10 minutes. The reaction was re-cooled to –78 °C, aldehyde **21** (318 mg, 0.75 mmol) in anhydrous THF (1 mL) was added dropwise and the reaction was stirred for 1 h at –78 °C. HMPA (196 µL, 1.13 mmol) was added and the reaction was allowed to warm to room temperature and stirred for a further 16 h. The reaction was quenched by the addition of sat. aq. NH_4Cl (5 mL) and the aqueous layer was extracted with EtOAc (3 × 10 mL). The combined organic layers were washed with H_2O (2 × 10 mL) and brine (1 × 10 mL), dried ($MgSO_4$), filtered and the solvent removed *in vacuo*. The crude residue was purified by column chromatography eluting with 3% Et_2O in petroleum ether (40-60 °C) to give **22** as a colourless oil (250 mg, 63%). ^{1}H NMR showed a 16,17 *E*:*Z* ratio of 4:1. $[\alpha]_D^{23}$ = –8.6 (*c* 0.7, $CHCl_3$). **δ_H** (400 MHz, $CDCl_3$) 6.74 (1H, td, *J* 7.5, 1.5, 3-H), 6.24 – 5.91 (5 H, overlapping m, 9-H, 10-H, 17-H, 18-H and 19-H), 5.68 (2H, overlapping m, 11-H and 16-H), 5.51 (2H, overlapping m, 8-H and 21-H), 4.10 (1H, m, 7-H), 3.73 (3H, s, CO_2CH_3), 2.19 – 2.01 (6H, overlapping m, 4-H_2, 12-H_2 and 15-H_2), 1.82 (3H, d, *J* 1.5, 2-CH_3), 1.73 (3H, overlapping s, 20-CH_3), 1.72 (3H, overlapping d, *J* 7.5, 22-H_3), 1.55– 1.37 (8H, overlapping m, 5-H, 6-H, 13-H and 14-H), 0.88 (9H, s, SiC(CH_3)$_3$), 0.04 (3H, s, Si(CH_3)$_2$), 0.02 (3H, s, Si(CH_3)$_2$). **δ_C** (126 MHz, $CDCl_3$) 168.9 (C-1), 142.7 (C-3), 135.7(C-19), 134.9 (C-20), 134.5 (C-8 and C-11),

133.7(C-16), 131.2 (C-17), 129.9 (C-9 and C-10), 127.7 (C-2), 126.7 (C-21), 126.3 (C-18), 73.3 (C-7), 51.8 (CO_2CH_3), 38.2 (C-6), 32.8 (C-12 or C-15), 32.7 (C-12 or C-15), 29.2 (C-13 or C-14), 28.9 (C-13 or C-14), 28.8 (C-4), 26.1 ($SiC(CH_3)_3$), 24.4 (C-5), 18.4 ($SiC(CH_3)_3$), 14.1 (21-CH_3), 12.6 (2-CH_3), 12.1 (C-22), –4.1 ($Si(CH_3)_2$), –4.6 ($Si(CH_3)_2$). **HRMS** (ESI) calc. for $[C_{31}H_{52}O_3Si+Na]^+$ 523.3583, found 523.3578. **IR** (ν_{max}/cm^{-1}) 2927, 2856, 1715. NB The assignment of the major isomer as the (*E*) product was facilitated by ^{1}H NMR data acquisition on a 700 MHz spectrometer which allowed the resolution of the overlapping 11-H and 16-H signals and thus the $J_{16\text{-}17}$ coupling value to be determined as 15.0 Hz.

4-Methoxy-5-methylenefuran-2(5*H*)-one 11

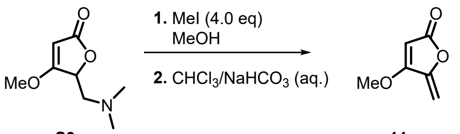

S9 **11**

Under an atmosphere of nitrogen, tetronate **S9,**[12] (4.93 g, 28.8 mmol) was dissolved in anhydrous MeOH (30 mL). Iodomethane (7.17 mL, 115 mmol) was added dropwise, and the reaction was stirred at room temperature for 16 h. The reaction mixture was concentrated *in vacuo* and the residue was dissolved in $CHCl_3$ (10 mL) and sat. aq. $NaHCO_3$ (36 mL). The mixture was stirred at room temperature for 2 h. The aqueous layer was extracted with CH_2Cl_2 (3 × 50 mL), the organic layers combined, dried ($MgSO_4$), filtered and the solvent removed *in vacuo*. The crude residue was purified by column chromatography, eluting with 50-60% EtOAc in petroleum ether (40-60 °C) to give **11** as a white solid (2.79 g, 77%). **δ_H** (400 MHz, $CDCl_3$) 5.25 (1H, dd, *J* 1.5, 0.5, 3-H), 5.05 (1H, dd, *J* 2.5, 1.5, 6-*H*H), 5.03 (1H, dd, *J* 2.5, 0.5, 6-H*H*), 3.93 (3H, s, OCH_3). **δ_C** (101 MHz, $CDCl_3$) 169.9 (C-4), 168.4 (C-2), 149.9 (C-5), 92.5 (C-6), 90.1 (C-3), 59.4 (OCH_3). **MS** (EI) calc. for $[C_6H_6O_3]$ 126.0, found 126.0.

((*S*,2*E*,8*E*,10*E*,16*E*,18*E*,20*E*)-1-(5′-Methoxy-4′-methylene-2′-oxo-2′,4′-dihydrofuran-1′-yl)-7-((*tert*-butyldimethylsilyl)oxy)-2,20-dimethyldocosa-2,8,10,16,18,20-hexaen-1-one 226

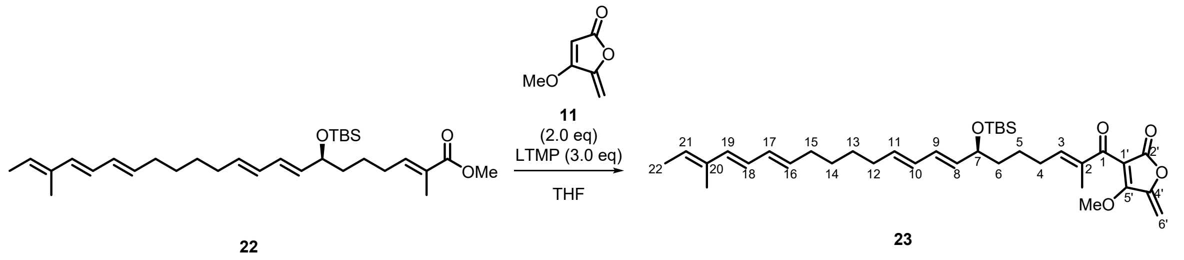

Adapting a procedure reported by Montgomery,[13] under an atmosphere of nitrogen, 2,2,6,6-tetramethylpiperidine (170 µL, 1.00 mmol) was dissolved in anhydrous THF (1.4 mL) and the solution cooled to –78 °C. *n*-BuLi (2.4 M in hexane, 438 µL, 1.05 mmol) was added dropwise

and the solution stirred at −78 °C for 15 minutes. The solution was allowed to warm to room temperature and stirred for a further 15 minutes to give a 0.5 M solution of LTMP that was re-cooled to −78 °C.

In a separate flask, under an atmosphere of nitrogen, tetronate **11** (68 mg, 0.540 mmol) was dissolved in anhydrous THF (8 mL) and the solution cooled to −78 °C. LTMP (0.5 M in THF, 1.62 mL, 0.810 mmol) was added dropwise and the reaction was stirred for 45 minutes at −78 °C. A solution of ester **22** (135 mg, 0.270 mmol) in THF (2 mL) was added dropwise and the reaction mixture was stirred at −78 °C for 24 h. The reaction was quenched by the addition of sat. aq. citric acid (8 mL) and the aqueous layer was extracted with CH_2Cl_2 (3 × 20 mL). The combined organic layers were dried ($MgSO_4$), filtered and the solvent removed *in vacuo*. The crude residue was purified by column chromatography, eluting with 10% EtOAc in petroleum ether (40-60 °C) to give **23** as a colourless oil (86 mg, 53%). ^{1}H NMR showed a 16,17 *E*:*Z* ratio of 7:1. $[\alpha]_D^{23}$ = +4.8 (*c* 0.8, $CHCl_3$). **δ$_H$** (500 MHz, $CDCl_3$) 6.57 (1H, tq, *J* 7.5, 1.0, 3-H), 6.19 – 5.92 (5H, overlapping m, 9-H, 10-H, 17-H, 18-H and 19-H), 5.65 (2H, overlapping m, 11-H and 16-H), 5.51(2H, overlapping m, 8-H and 21-H), 5.16 (1H, d, *J* 3.0, 6′-*H*H), 5.15 (1H, d, *J* 3.0, 6′-H*H*), 4.12 (1H, app. q, *J* 6.0, 7-H), 3.90 (3H, s, OCH_3), 2.31 (2H, app. q, *J* 7.0, 4-H_2), 2.09 (4H, overlapping m, 12-H_2 and 15-H_2), 1.87 (3H, d, *J* 1.0, 2-CH_3), 1.74 (3H, overlapping s, 20-CH_3), 1.73 (3H, overlapping d, *J* 8.0, 22-H_3), 1.51 (4H, overlapping m, 5-H_2 and 6-H_2), 1.40 (4H, overlapping m, 13-H_2 and 14-H_2), 0.88 (9H, s, $SiC(CH_3)_3$), 0.03 (3H, s, $Si(CH_3)_2$), 0.02 (3H, s, $Si(CH_3)_2$). **δ$_C$** (126 MHz, $CDCl_3$) 191.2 (C-1), 166.3 (C-2′), 165.5 (C-5′), 151.4 (C-3), 149.1 (C-4′), 138.5 (C-2), 135.7 (C-19), 134.9 (C-20), 134.6 (C-8), 134.2 (C-11), 133.7 (C-16), 131.2 (C-17 or C-10), 130.0 (C-17 or C-10), 129.8 (C-9), 126.7 (C-21), 126.3 (C-18), 104.9 (C-1′), 94.3 (C-6′), 73.0 (C-7), 61.0 (OCH_3), 38.2 (C-6), 32.8 (C-12 or C-15), 32.7 (C-12 or C-15), 29.7 (C-4), 29.2 (C-13 or C-14), 28.9 (C-13 or C-14), 26.0 ($SiC(CH_3)_3$), 24.0 (C-5), 18.4 ($SiC(CH_3)_3$), 14.1 (C-22), 12.1 (20-CH_3), 11.3 (2-CH_3), –4.1 ($Si(CH_3)_2$), –4.7 ($Si(CH_3)_2$). **IR** (ν_{max}/cm^{-1}, film) 2928, 2856, 1775, 1631. Not observed by ESI or APCI MS.

((*S*,2*E*,8*E*,10*E*,16*E*,18*E*,20*E*)-1-(5′-Methoxy-4′-methylene-2′-oxo-2′,4′-dihydrofuran-1′-yl)-7-hydroxy-2,20-dimethyldocosa-2,8,10,16,18,20-hexaen-1-one 24

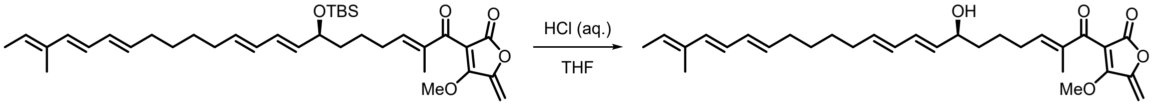

23 24

Tetronate **23** (15.0 mg, 0.0252 mmol) was dissolved in THF (1 mL) and the solution cooled to 0 °C. 2 M aq. HCl (378 µL, 0.756 mmol) was added and the reaction stirred at 0 °C for 1 h. A further portion of 2 M aq. HCl (378 µL, 0.756 mmol) was added and the reaction was allowed

to warm to room temperature and stirred for a further 1.5 h. The reaction was quenched by the addition of sat. aq. $NaHCO_3$ (2 mL) and the aqueous layer was extracted with EtOAc (3 × 10 mL). The combined organic layers were, dried ($MgSO_4$), filtered and the solvent removed *in vacuo*. The crude residue was purified by column chromatography, eluting with 30% EtOAc in petroleum ether (40-60 °C) to give **24** as a colourless oil (11.0 mg, 91%). ^{1}H NMR showed a 16,17 *E*:*Z* ratio of 9:1. $[\alpha]_D^{22}$= +4.0 (*c* 1.0, $CHCl_3$). **δ_H** (500 MHz, $CDCl_3$) 6.58 (1H, tq, *J* 7.5, 1.5, 3-H), 6.24 – 5.97 (5H, overlapping m, 9-H, 10-H, 17-H, 18-H and 19-H), 5.68 (2H, overlapping m, 11-H and 16-H), 5.55 (2H, overlapping m, 8-H and 21-H) 5.17 (1H, d, *J* 3.0, 6′-*H*H), 5.15 (1H, d, *J* 3.0, 6′-H*H*), 4.12 (1H, m, 7-H), 3.91 (3H, s, OCH_3), 2.35 (2H, m, 4-H_2), 2.09 (4H, overlapping m, 12-H_2 and 15-H_2), 1.88 (3H, d, *J* 1.5, 2-CH_3), 1.74 (3H, overlapping s, 20-CH_3), 1.73 (3H, overlapping d, *J* 8.0, 22-H_3), 1.55 (4H, overlapping m, 6-H_2 and 5-H_2) 1.40 (4H, overlapping m, 13-H_2 and 14-H_2). **δ_C** (126 MHz, $CDCl_3$) 191.2 (C-1), 166.4 (C-2′), 165.7 (C-5′), 151.1 (C-3), 149.0 (C-4′), 138.5 (C-2), 135.8 (C-19), 135.7 (C-11), 134.9 (C-20), 133.6 (C-16), 133.3 (C-8), 131.4 (C-9), 131.2 (C-17), 129.6 (C-10), 126.8 (C-21), 126.3 (C-18), 104.9 (C-1′), 94.4 (C-6′), 72.6 (C-7), 61.1 (OCH_3), 36.9 (C-6), 32.8 (C-12 or C-15), 32.6 (C-12 or C-15), 29.6 (C-4), 29.1 (C-13 or C-14), 28.8 (C-13 or C-14), 24.3 (C-5), 14.1 (C-22), 12.1 (20-CH_3), 11.3 (2-CH_3). **HRMS** (ESI) calc. for $[C_{30}H_{40}O_5+Na]^+$ 503.2768 found 503.2769. **IR** (ν_{max}/cm^{-1}, film) 3429, 2924, 2852, 1772, 1628.

Methyl (*S*,2*E*,8*E*,10*E*)-7-((*tert*-butyldimethylsilyl)oxy)-2-methylheptadeca-2,8,10,16-tetraenoate 26

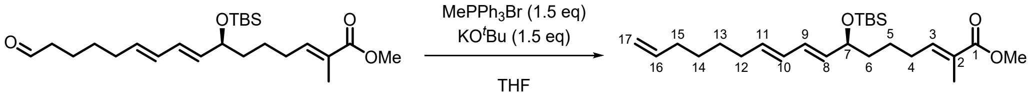

21 26

Under an atmosphere of nitrogen, methyltriphenylphosphonium bromide (265 mg, 0.741 mmol) was dissolved in anhydrous THF (5 mL). The solution was cooled to 0 °C and KO*t*Bu (1.0 M in THF, 0.74 mmol, 0.74 mL) was added dropwise and the solution stirred at 0 °C for 1 h. Aldehyde **21** (209 mg, 0.494 mmol) in anhydrous THF (1 mL) was added dropwise and the reaction mixture was allowed to warm to room temperature and stirred for 1 h. The reaction was quenched by the addition of sat. aq. NH_4Cl (6 mL) and the aqueous layer was extracted with EtOAc (3 × 20 mL). The combined organic layers were dried ($MgSO_4$), filtered and the solvent removed *in vacuo*. The crude residue was purified by column chromatography eluting with 5% EtOAc in petroleum ether (40-60 °C) to give **26** as a colourless oil (176 mg, 85%). $[\alpha]_D^{24}$= –6.0 (*c* 1.0, $CHCl_3$). **δ_H** (400 MHz, $CDCl_3$) 6.73 (1H, tq, *J* 7.5, 1.5, 3-H), 6.03 (2H, overlapping m, 9-H and 10-H), 5.80 (1H, ddt, *J* 17.0, 10.0, 6.5, 16-H), 5.63 (1H, dt, *J* 15.0, 7.0, 11-H), 5.49 (1H, dd, *J* 15.0, 7.0, 8-H), 5.00 (1H, dt, *J* 17.0, 1.5, 17-*H*H), 4.93 (1H, ddt, *J* 10.0,

2.5, 1.0, 17-H*H*), 4.11 (1H, m, 7-H), 3.73 (3H, s, CO_2CH_3), 2.15 (2H, app. q, *J* 7.0, 4-H_2), 2.12 – 2.01 (4H, overlapping m, 15-H_2 and 12-H_2), 1.82 (3H, d, *J* 1.5, 2-CH_3), 1.59 – 1.36 (8H, overlapping m, 5-H_2, 6-H_2, 13-H_2 and 14-H_2), 0.89 (9H, s, $SiC(CH_3)_3$), 0.04 (3H, s, $Si(CH_3)_2$), 0.02 (3H, s, $Si(CH_3)_2$). **δ_C** (101 MHz, $CDCl_3$) 168.8 (C-1), 142.6 (C-3), 139.1 (C-16), 134.5 (C-8 and C-11), 129.9 (C-9 and C-10), 127.7 (C-2), 114.5 (C-17), 73.2 (C-7), 51.8 (OCH_3), 38.2 (C-6), 33.8 (C-15), 32.6 (C-12), 28.9 (C-13), 28.8 (C-5), 28.6 (C-14), 26.1 (SiC(*C*H_3)$_3$), 24.4 (C-5), 18.4 (Si*C*(CH_3)$_3$), 12.5 (2-CH_3), –4.1 ($Si(CH_3)_2$), –4.6 ($Si(CH_3)_2$). **IR** (ν_{max}/cm^{-1}, film) 2829, 2856, 1717, 1642. Not observed by ESI or APCI MS.

(*S*,2*E*,8*E*,10*E*)-7-((*tert*-Butyldimethylsilyl)oxy)-1-(5′-methoxy-4′-methylene-2′-oxo-2′,4′-dihydrofuran-1′-yl)-2-methylheptadeca-2,8,10,16-tetraen-1-one S10

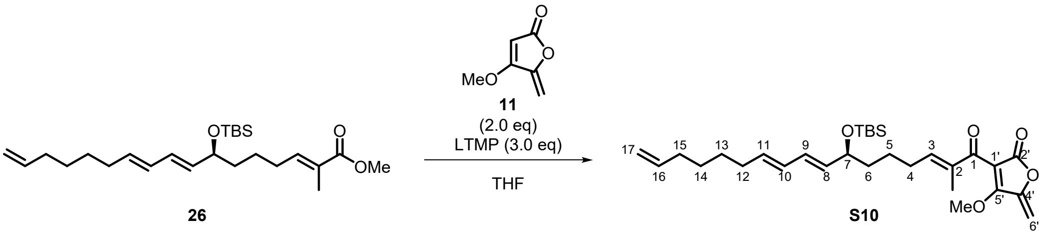

Under an atmosphere of nitrogen, 2,2,6,6-tetramethylpiperidine (204 µL, 1.20 mmol) was dissolved in anhydrous THF (1.7 mL) and the solution cooled to –78 °C. *n*-BuLi (2.4 M in hexane, 0.53 mL, 1.26 mmol) was added dropwise and the solution stirred at –78 °C for 15 minutes. The solution was allowed to warm to room temperature and stirred for a further 15 minutes to give a 0.5 M solution of LTMP that was re-cooled to –78 °C.

In a separate flask, under an atmosphere of nitrogen, tetronate **11** (81 mg, 0.642 mmol) was dissolved in anhydrous THF (10 mL) and the solution cooled to –78 °C. LTMP (0.5 M in THF, 1.93 mL, 0.963 mmol) was added dropwise and the reaction was stirred for 30 minutes at –78 °C. A solution of ester **26** (135 mg, 0.321 mmol) in THF (1 mL) was added dropwise and the reaction mixture was stirred at –78 °C for 24 h. The reaction was quenched by the addition of sat. aq. citric acid (10 mL) and the aqueous layer was extracted with CH_2Cl_2 (3 × 20 mL). The combined organic layers were dried ($MgSO_4$), filtered and the solvent removed *in vacuo*. The crude residue was purified by column chromatography, eluting with 5% EtOAc in petroleum ether (40-60 °C) to give **S10** as a colourless oil (78 mg, 47%). $[\alpha]_D^{23}$ = –4.0 (*c* 1.0, $CHCl_3$). **δ_H** (400 MHz, $CDCl_3$) 6.57 (1H, td, *J* 7.5, 1.5, 3-H), 6.02 (2H, m, 9-H and 10-H), 5.80 (1H, ddt, *J* 17.0, 10.0, 6.5, 16-H), 5.63 (1H, dt, *J* 14.5, 6.5, 11-H), 5.48 (dd, *J* 14.5, 6.5, 8-H), 5.16 (1H, d, *J* 3.0, 6′-*H*H), 5.14 (1H, d, *J* 3.0, 6′-H*H*), 4.99 (1H, dt, *J* 17.0, 1.5, 17-*H*H), 4.93 (1H, dt, *J* 10.0, 2.5, 17-H*H*), 4.11 (1H, q, *J* 6.0, 7-H), 3.90 (3H, s, OCH_3), 2.33 (2H, m, 4-H_2), 2.05 (4H, m, 15-H_2 and 12-H_2), 1.87 (3H, d, *J* 1.5, 2-CH_3), 1.53 – 1.32 (8H, overlapping m, 5-H_2, 6-H_2, 13-

H_2 and 14-H_2), 0.87 (9H, s, SiC(CH_3)$_3$), 0.03 (3H, s, Si(CH_3)$_2$), 0.01 (3H, s, Si(CH_3)$_2$). **δ_C** (101 MHz, $CDCl_3$) 191.2 (C-1), 166.2 (C-2′), 165.4 (C-5′), 151.3 (C-3), 149.0 (C-4′), 139.0 (C-16), 138.5 (C-2), 134.6 (C-11), 134.1 (C-8), 130.0 (C-10), 129.8 (C-9), 114.4 (C-17), 104.8 (C-1′), 94.2 (C-6′), 73.0 (C-7), 61.0 (OCH_3), 38.1 (C-6), 33.7 (C-15), 32.6 (C-12), 29.6 (C-4), 28.8 (C-13), 28.6 (C-14), 26.0 (SiC(CH$_3$)$_3$), 24.0 (C-5), 18.3 (SiC(CH_3)$_3$), 11.3 (2-CH_3), –4.2 (Si(CH_3)$_2$), –4.7 (Si(CH_3)$_2$). **HRMS** (ESI) calc. for $[C_{30}H_{46}O_5Si+Na]^+$ 537.3007, found 537.2291. **IR** (v_{max}/cm^{-1}, film) 2928, 2856, 1775, 1638.

(*S*,2*E*,8*E*,10*E*)-7-hydroxy-1-(5′-methoxy-4′-methylene-2′-oxo-2′,4′-dihydrofuran-1′-yl)-2-methylheptadeca-2,8,10,16-tetraen-1-one 27

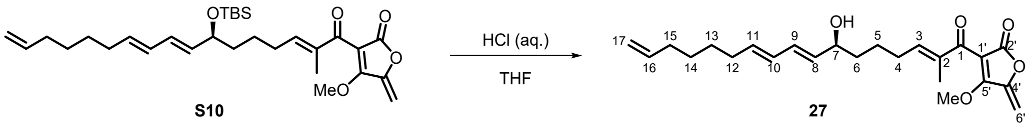

Tetronate **S10** (32 mg, 0.062 mmol) was dissolved in THF (3 mL) and the solution cooled to 0 °C. 2 M aq. HCl (930 µL, 1.86 mmol) was added and the reaction stirred at 0 °C for 30 minutes. A further portion of 2 M aq. HCl (930 µL, 1.86 mmol) was added and the reaction was allowed to warm to room temperature and stirred for a further 2 h. The reaction was quenched by the addition of sat. aq. $NaHCO_3$ (6 mL) and the aqueous layer was extracted with EtOAc (3 × 20 mL). The combined organic layers were dried ($MgSO_4$), filtered and the solvent removed *in vacuo*. The crude residue was purified by column chromatography, eluting with 30% EtOAc in petroleum ether (40-60 °C) to give **27** as a colourless oil (19 mg, 76 %). $[\alpha]_D^{21}$= –4.0 (*c* 1.0, $CHCl_3$). **δ_H** (500 MHz, $CDCl_3$) 6.58 (1H, t, *J* 7.5, 3-H), 6.17 (1H, dd, *J* 15.5, 10.5, 9-H), 6.01 (1H, dd, *J* 15.0, 10.5, 10-H), 5.80 (1H, ddt, *J* 17.0, 10.0, 7.0, 16-H), 5.70 (1H, dt, *J* 14.5, 7.0, 11-H), 5.55 (1H, dd, *J* 15.0, 7.0, 8-H), 5.17 (1H, d, *J* 2.5, 6′-*H*H), 5.15 (1H, d, J 2.5, 6′-H*H*), 4.99 (1H, app. dq, *J* 17.0, 2.0, 17-*H*H), 4.94 (1H, m, 17-H*H*), 4.12 (1H, q, *J* 6.0, 7-H), 3.91 (3H, s, OCH_3), 2.34 (2H, m, 4-H_2), 2.06 (4H, overlapping m, 15-H_2 and 12-H_2), 1.88 (3H, s, 2-CH_3), 1.56 (4H, m, 6-H_2 and 5-H_2), 1.40 (4H, m, 13-H_2 and 14-H_2). **δ_C** (151 MHz, $CDCl_3$) 191.2 (C-1), 166.4 (C-2′), 165.7 (C-5′), 151.0 (C-3), 149.1 (C-4′), 139.0 (C-16), 138.5 (C-2), 135.8 (C-11), 133.3 (C-8), 131.4 (C-9), 129.6 (C-10), 114.5 (C-17), 104.9 (C-1′), 94.4 (C-6′), 72.6 (C-7), 61.1 (OCH_3), 36.9 (C-6), 33.8 (C-15), 32.6 (C-12), 29.6 (C-4), 28.8 (C-13), 28.6 (C-14), 24.3 (C-5), 11.3 (2-CH_3). **HRMS** (ESI) calc. for $[C_{24}H_{32}O_5+Na]^+$ 423.2142, found 423.2122. **IR** (v_{max}/cm^{-1}, film) 3462, 2927, 2856, 1771, 1632, 1455.

1.9 Enzymatic Reaction Products

Decalin 28

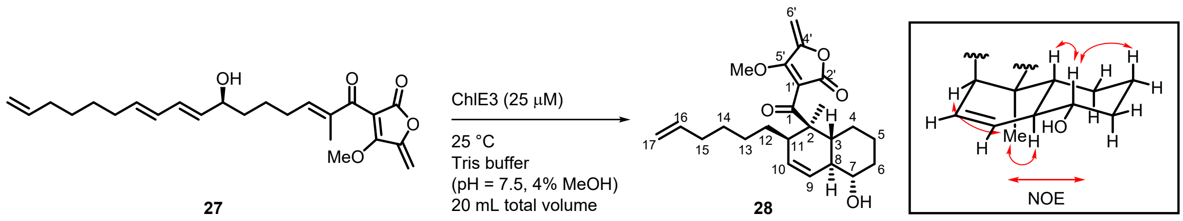

28: **δ_H** (700 MHz, $CDCl_3$) 5.96 (1H, d, *J* 10.0, 9-H), 5.84 (1H, ddd, *J* 10.0, 5.0, 2.5, 10-H), 5.78 (1H, ddt, *J* 17.0, 10.0, 6.5, 16-H), 5.19 (1H, d, *J* 3.0, 6′-*H*H), 5.15 (1H, d, *J* 3.0, 6′-H*H*), 4.96 (1H, dd, *J* 17.0, 2.0, 17-*H*H), 4.90 (1H, m, 17-H*H*), 3.94 (3H, s, OCH_3), 3.36 (1H, td, *J* 10.5, 4.5, 7-H), 3.02 (1H, m, 11-H), 2.06 – 2.02 (1H, m, 6-*H*H), 2.02 – 1.98 (2H, m, 15-H_2), 1.85 (1H, td, *J* 11.0, 2.0, 3-H), 1.76 (1H, m, 5-*H*H), 1.65 (1H, m, 8-H), 1.48 – 1.42 (2H, m, 4-*H*H and 5-H*H*), 1.40 – 1.32 (5H, m, 6-H*H*, 13-H_2 and 14-H_2), 1.28 (3H, s, 2-CH_3), 1.18 (2H, m, 12-H_2), 0.85 (1H, m, 4-H*H*). **δ_C** (176 MHz, $CDCl_3$) 203.6 (C-1), 167.6 (C-5′), 165.4 (C-2′), 149.3 (C-4′), 139.3 (C-16), 129.5 (C-10), 124.4 (C-9), 114.3 (C-17), 108.5 (C-1′), 94.8 (C-6′), 74.0 (C-7), 63.0 (OCH_3), 54.6 (C-2), 46.3 (C-8), 41.8 (C-11), 38.1 (C-3), 36.4 (C-6), 34.2 (C-12), 33.9 (C-15), 29.4 (C-14), 26.8 (C-13), 26.4 (C-4), 24.4 (C-5), 17.5 (2-CH_3).

Spirotetronate 30a and 30b

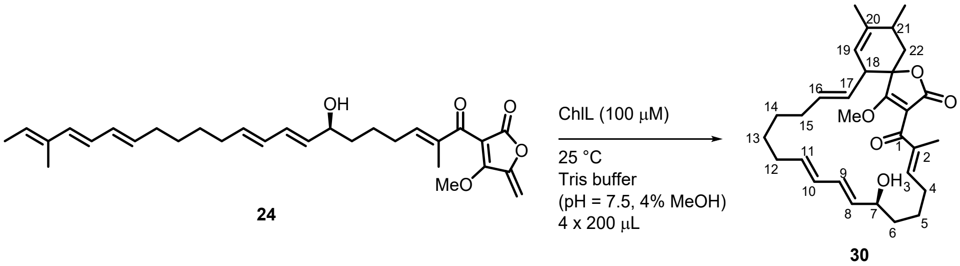

24

30
(2 diastereomers)

30a: (t_R = 11.1) δ_H (700 MHz, CDCl$_3$): 6.55 (1H, t, *J* 7.5, 3-H) , 6.14 (1H, dd, *J* 15.5, 10.5, 9-H), 6.00 (1H, dd, *J* 15.0, 10.5, 10-H), 5.60 (1H, ddd, *J* 15.0, 8.5, 6.0, 11-H), 5.52 (1H, dd, *J* 15.5, 8.0, 8-H), 5.38 (1H, m, 16-H), 5.28 (1H, dd, 15.5, 7.5, 17-H), 5.21 (1H, br s, 19-H), 4.14 (1H, td, *J* 8.0, 4.0, 7-H) 3.70 (3H, s, OCH$_3$), 2.77 (1H, br t, *J* 5.0, 18-H), 2.43 (1H, br s, 21-H), 2.36 (1H, m, 4-*H*H), 2.23 (1H, m, 4-H*H*), 2.17 (1H, m, 12-*H*H), 2.05 (1H, m, 12-H*H*), 1.96 (2H, q, *J* 7.5, 15-H$_2$), 1.87 (3H, s, 2-CH$_3$), 1.77 – 1.66 (6H, m, 20-CH$_3$, 6-*H*H, 22-H$_2$), 1.50 (3H, m, 6-H*H* and 13-H$_2$), 1.39 – 1.26 (4H, m, 5-H$_2$, and 14-H$_2$), 1.07 (3H, d, *J* 7.0, 21-CH$_3$).

30b: (t_R = 12.3) δ_H (700 MHz, CDCl$_3$): 6.49 (1H, t, *J* 7.5, 3-H), 6.17 (1H, dd, *J* 15.5, 10.5, 9-H), 6.06 (1H, dd, J 15.5, 10.5, 9-H, 10-H), 5.64 (1H, ddd, J 15.0, 8.5, 6.0, 11-H), 5.51 (2H, m, 8-H and 16-H), 5.28 (1H, dd, *J* 15.5, 8.5, 17-H), 5.12 (1H, br s, 19-H), 4.18 (1H, td, *J* 8.0, 4.0, 7-H), 3.86 (3H, s, OCH$_3$), 3.11 (1H, br d, *J* 8.5, 18-H), 2.31 (1H, br s, 21-H), 2.20 (3H, m, 4-H$_2$ and 12-*H*H), 2.13 (1H, dd, *J* 14.0, 7.0, 22-*H*H), 2.07 (1H, m, 12-H*H*), 1.95 (2H, q, *J* 7.5, 15-H$_2$), 1.81 (3H, s, 2-CH$_3$), 1.78 (1H, m, 6-*H*H) 1.73 (3H, s, 20-CH$_3$), 1.71 (1H, dd, *J* 14.0, 2.5, 22-H*H*), 1.64 (1H, m, 6-H*H*), 1.50 (2H, m, 13-H$_2$), 1.44 (2H, m, 5-H$_2$), 1.39 – 1.31 (2H, m, 14-H$_2$). 1.18 (3H, d, *J* 7.0, 21-CH$_3$).

Spirotetronate 29

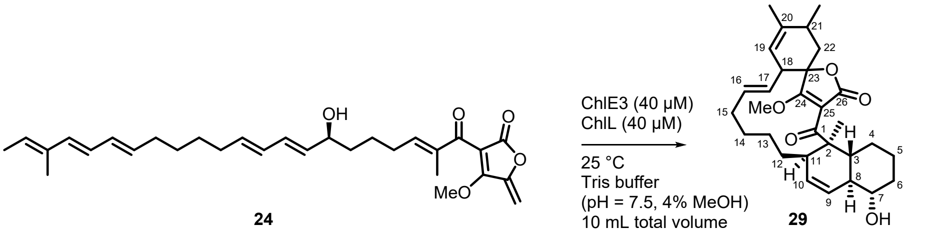

24

29

29: δ_H (700 MHz, CDCl$_3$): 5.81 (1H, d, *J* 10.0, 9-H), 5.64 (1H, ddd, *J* 10.0, 5.5, 2.4, 10-H), 5.13 (2H, m, 16-H and 19-H), 5.00 (1H, dd, *J* 15.5, 9.0, 17-H), 3.98 (3H, s, OCH$_3$), 3.31 (1H, td, *J* 10.0, 5.5, 7-H), 3.11 (1H, br d, *J* 9.5, 18-H), 2.96 (1H, br t, *J* 6.5, 11-H), 2.29 (2H, m, 22-HH and 21-H), 2.14 (1H, m, 15-*H*H), 2.03 (1H, m, 6-*H*H), 2.00 (1H, m, 4-*H*H), 1.73 (5H, m, 3-H, 5-*H*H and 20-CH$_3$), 1.69 (1H, m, 22-H*H*), 1.67 (1H, m, 8-H), 1.57 (1H, m, 15-H*H*), 1.46 (3H, s, 2-CH$_3$), 1.44 (1H, m, 5-H*H*), 1.32 – 1.23 (6H, m, 6-H*H*, 13-*H*H, 14-*H*H and 21-CH$_3$), 1.01 (2H,

m, 12-*H*H and 13-H*H*), 0.90 (2H, m, 12-H*H* and 14-H*H*), 0.75 (1H, m, 4-H*H*). **δ_C** (126 MHz, $CDCl_3$): 200.9 (C-1), 191.0 (C-24), 169.0 (C-26), 137.6 (C-20), 135.6 (C-16), 131.9 (C-10), 128.7 (C-17), 122.5 (C-9), 120.7 (C-19), 109.6 (C-25), 83.3 (C-23), 74.3 (C-7), 64.7 (OCH_3), 52.5 (C-2), 46.3 (C-18), 46.0 (C-8), 43.1 (C-11), 38.8 (C-3), 36.5 (C-6), 35.8 (C-22), 33.0 (C-15), 32.1 (C-13), 32.0 (C-21), 27.5 (C-12), 27.2 (C-4), 26.6 (C-14), 24.5 (C-5), 22.1 (20-CH_3), 20.0 (21-CH_3), 15.1 (2-CH_3).

2. Supplementary Tables

Table S1: [4+2]-cyclases from class II spirotetronate and spirotetramate BGCs.[14,15,16,17]

BGC	Decalin [4+2]-cyclase	Spirotetronate [4+2]-cyclase
pyr (pyrroindomycins A and B)	PyrE3	PyrI4
chl (chlorothricin and deschlorothricin)	ChlE3	ChlL
tca (tetrocarcin A)	TcaE1	TcaU4
kij (kijanimicin)	KijA	KijU
mak (maklamicin)	MakC1	Orf23
lob (lobophorins A and B)	LobP3	LobU2
vst (versipelostatin)	VstK	VstJ
plo (pyrrolosporin A)	PloE3	PloI4

Table S2: Statistics of X-ray data collection and refinement of ChlE3 structure (PDB: 9SRP)

**Data collection**	**ChlE3**
Beamline wavelength (Å)	0.99987
Space group	C 1 2 1
Cell dimensions	
a, b, c (Å)	191.87, 69.76, 188.72
a, b, g, (°)	90.00, 109.37, 90.00
Resolution (Å)	90.669 – 1.84 (1.87 – 1.84)[a]
R_{merge}	0.105 (2.386)[a]
No. of reflections	1350145 (65782)[a]
No. of unique reflections	204156 (10017)[a]
I/σI	8.2 (0.7)[a]
$CC_{1/2}$	0.996 (0.322)[a]

Completeness (%)	100.0 (100.0)[a]
Redundancy	6.6 (6.6)[a]
**Refinement**	
R_{work}/R_{free}	0.1831/ 0.2226
No. of atoms	
Protein	14297
Ligand/ion	212
Water	502
B factors Å^2	
Protein	55.51
Ligand/ion	38.94
Water	46.45
Root mean square deviations	
Bond lengths (Å)	0.0148
Bond angles (°)	2.3478
Ramachandran favored (%)	96.63
Ramachandran outliers (%)	0.05

(a) Values in parentheses are for highest resolution shell

3. Supplementary Figures

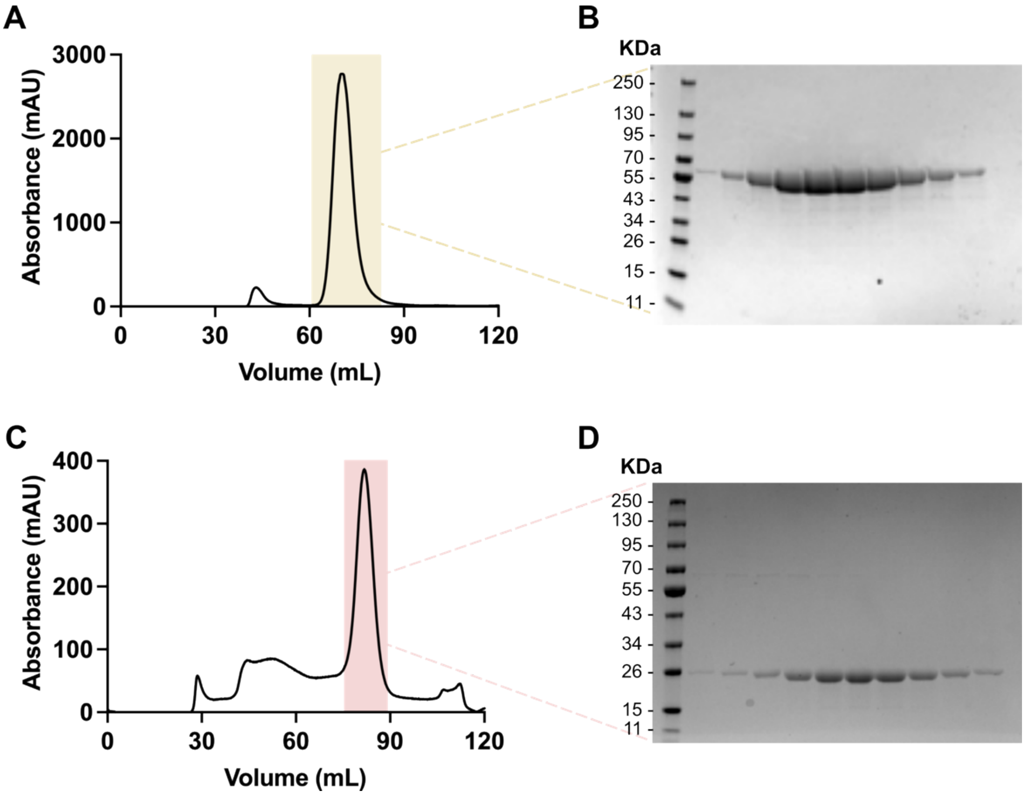

Figure S1. Purification of ChlE3 and ChlL. Size exclusion chromatogram of the purification of **A)** ChlE3 and **C)** ChlL. Quality control of the purification of **B)** ChlE3 and **D)** ChlL was followed by SDS-PAGE gel, recovering fractions of each recombinant enzyme from the size exclusion chromatography.

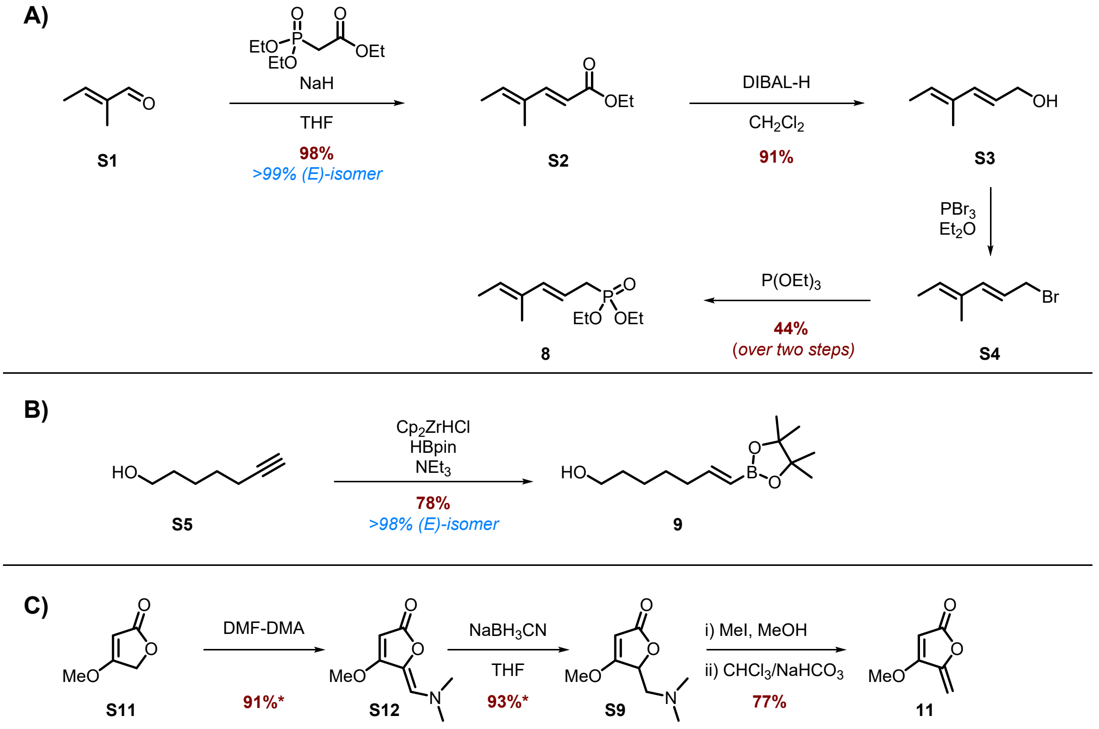

Figure S2: Synthetic routes to chlorothricin linear precursor fragments. **A)** Synthesis of phosphonate **8** **B)** Synthesis of vinyl boronate **9** **C)** Synthesis of vinyl iodide **10** **C)** Synthesis of *exo*-methylene tetronate **11**. *Procedures for first two steps reported in earlier publication.[12]

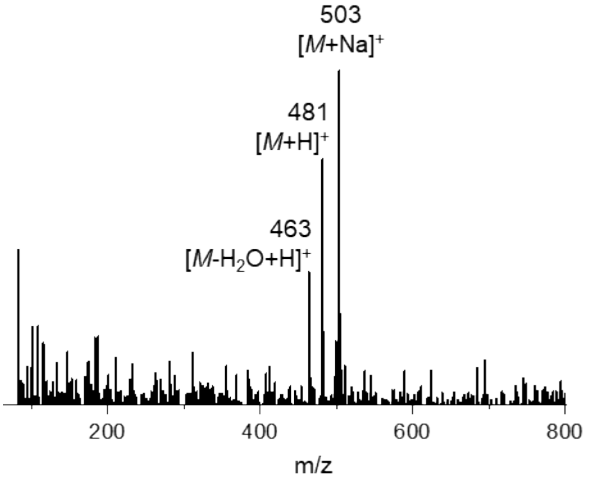

Figure S3: Positive mode ESI MS of ChlE3 assay product (t_R = 14.7).

4. Molecular docking studies

Molecular docking studies were performed using AutoDock Vina (version 1.1.2)[18] with an exhaustiveness of 32 and a box size of 20 Angstrom. Ligands were constructed in AceDRG[19] as deployed within the CCP4i2 suite. MGLTools (Version 1.5.7) was used to prepare protein structures, with ligands prepared using Obabel.[20]

5. Supplementary References

[1] P. R. Evans and G. N. Murshudov, *Acta Crystallogr. D. Biol. Crystallogr.* **2013**, *69, 1204-14.*

[2] A. J. McCoy, R. W. Grosse-Kunstleve , P. D. Adams, M.D. Winn, L. C. Storoni, R. J. Read, *J. Appl. Crystallogr.* **2007**, *40, 658-674.*

[3] P. Emsley, B. Lohkamp, W. G. Scott, K. Cowtan, *Acta Crystallogr. D Biol. Crystallogr.* **2010**, *66, 486-501.*

[4] G. N. Murshudov, P. Skubak, A. A. Lebedev, N. S. Pannu, R. A. Steiner, R. A. Nicholls, M. D. Winn, F. Long, A. A. Vagin, *Acta Crystallogr. D Biol. Crystallogr.* **2011**, *67, 355-367.*

[5] W. C. Still, M. Kahn, A. Mitra, *J. Org. Chem.* **1978**, *43, 2923–2925.*

[6] D. Enders, G. Geibel, S. Osborne, *Chemistry* **2000**, *6,1302-1309.*

[7] Y. D. Wang, G. Kimball, A. S. Prashad, Y. Wang, *Tetrahedron Lett.,* **2005**, *46, 8777-8780.*

[8] R. E. Boer, J. A. Giménez-Bastida, O. Boutaud, S. Jana, C. Schneider, G. A. Sulikowski, *Org. Lett.,* **2018**, *20, 4020-4022.*

[9] M. I. Thomson, G. S. Nichol, A. L. Lawrence, *Org. Lett.* **2017**, *19, 2199–2201.*

[10] A. Darwish and J. M. Chong, *Tetrahedron* **2012**, *68, 654–658.*

[11] E. J. Thomas and J. W. F. Whitehead, *J. Chem. Soc., Perkin Trans. 1* **1989**, *507-518.*

[12] A. J. Devine, A. E. Parnell, C. R. Back, N. R. Lees, S. T. Johns, A. Z. Zulkepli, R. Barringer, K. Zorn, J. E. M. Stach, M. P. Crump, M.A. Hayes, M. W. van der Kamp, P. R. Race, C. L. Willis, *Angew Chem Int Ed Engl.* **2023**, *62, e202213053.*

[13] L. J. Montgomery and G. L. Challis, *Synlett* **2008**, *2164–2168.*

[14] R. Daduang, S. Kitani, J. Hashimoto, A. Thamchaipenet, Y. Igarashi, K. Shin-ya, H. Ikeda, T. Nihira, *Microbiol Res.* **2015***, 180, 30-39.*

[15] T. Hashimoto, J. Hashimoto, K. Teruya, T. Hirano, K. Shin-ya, H. Ikeda, H. W. Liu, M. Nishiyama, T. Kuzuyama, *J. Am. Chem. Soc.* **2015***, 137, 2, 572–575.*

[16] H. Wang, Y. Zou, M. Li, Z. Tang, J. Wang, Z. Tian, N. Strassner, Q. Yang, Q. Zheng, Y. Guo, W. Liu, L. Pan and K. N. Houk, *Nat. Chem.* **2023***, 15, 177–184.*

[17] Z. Tian, P. Sun, Y. Yan, Z. Wu, Q. Zheng, S. Zhou, H. Zhang, F. Yu, X. Jia, D. Chen, T. Kurt and W. Liu, *Nat. Chem. Biol.* **2015***, 11, 259–265.*

[18] J. Eberhardt, D. Santos-Martins, A. F. Tillack, and S. Forli, *J. Chem. Inf. Model.*, **2021**, *61*, 3891- 3898.

[19] F. Long, R. A. Nicholls, P. Emsley, S. Grazulis, A. Merkys, A. Vaitkus and G. N. Murshudov, *Struct. Biol.*, **2017**, *73*, 112-122.

[20] N. M. O'Boyle, M. Banck, C. A. James, C. Morley, T. Vandermeersch, and G. R. Hutchison, *J. Cheminform*, **2011**, 3:33.

6. ¹H and ¹³C NMR Spectra

6.1 Synthetic Compounds

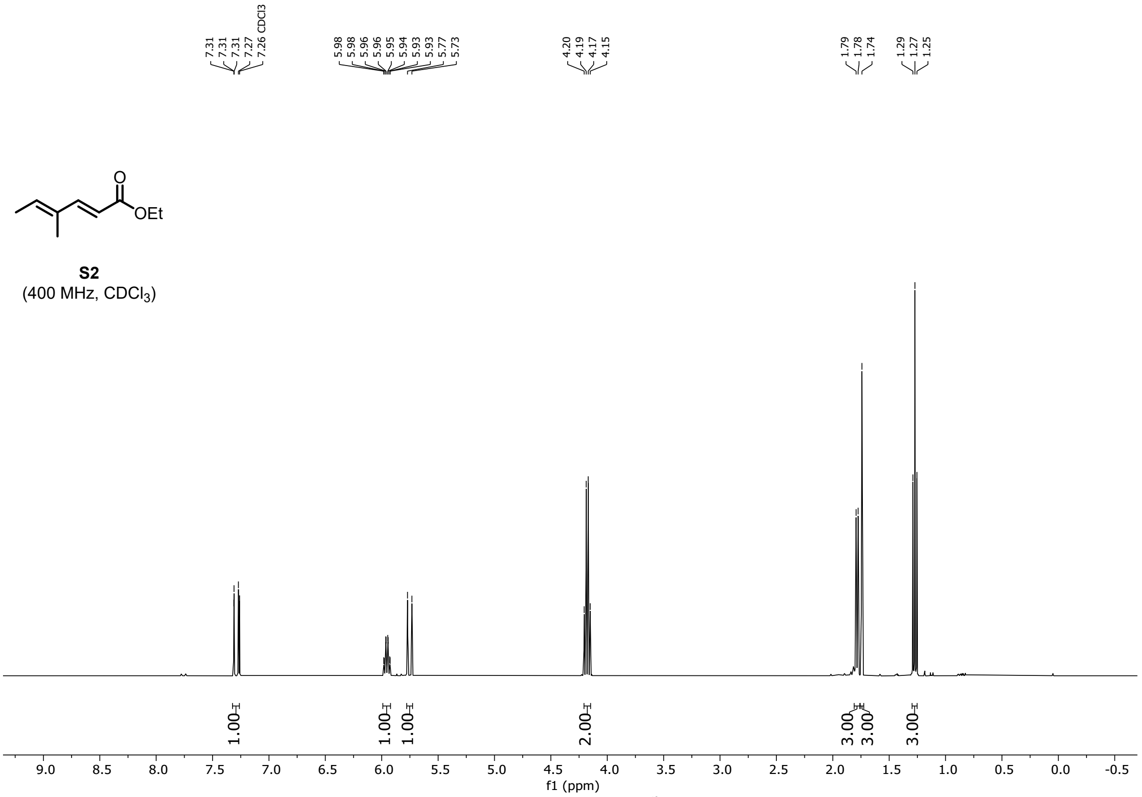

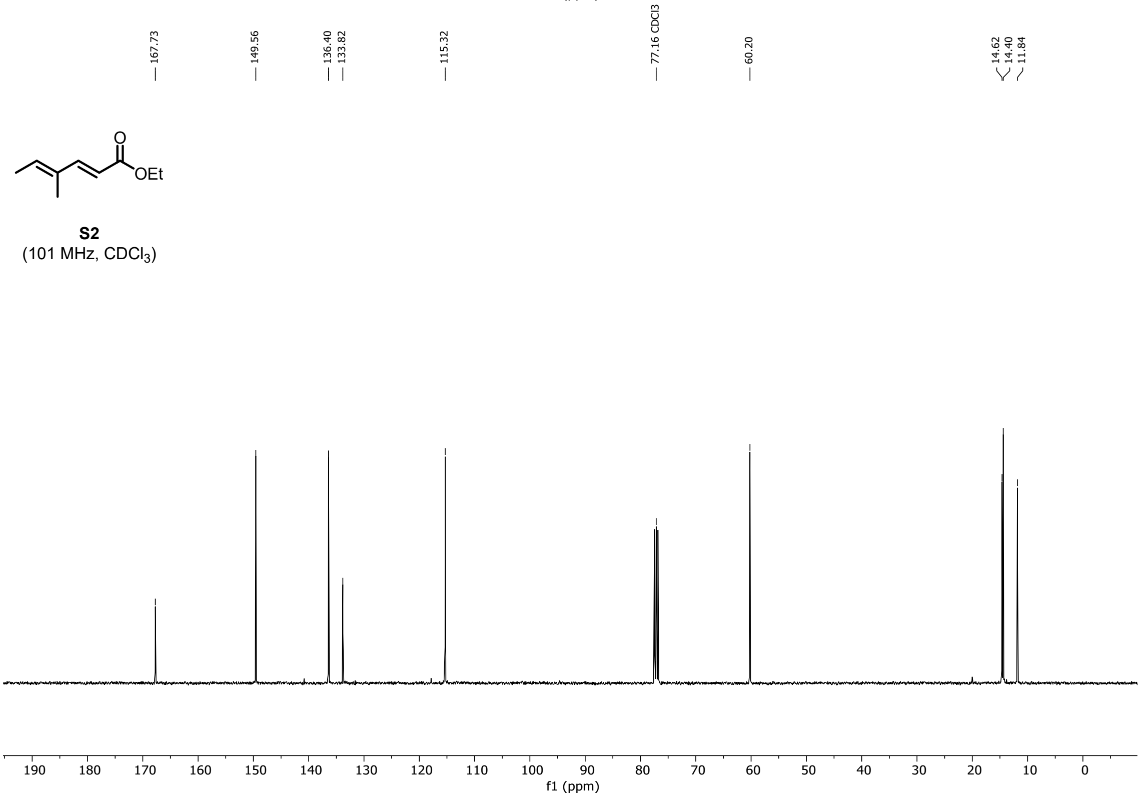

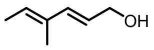

S3
(400 MHz, $CDCl_3$)

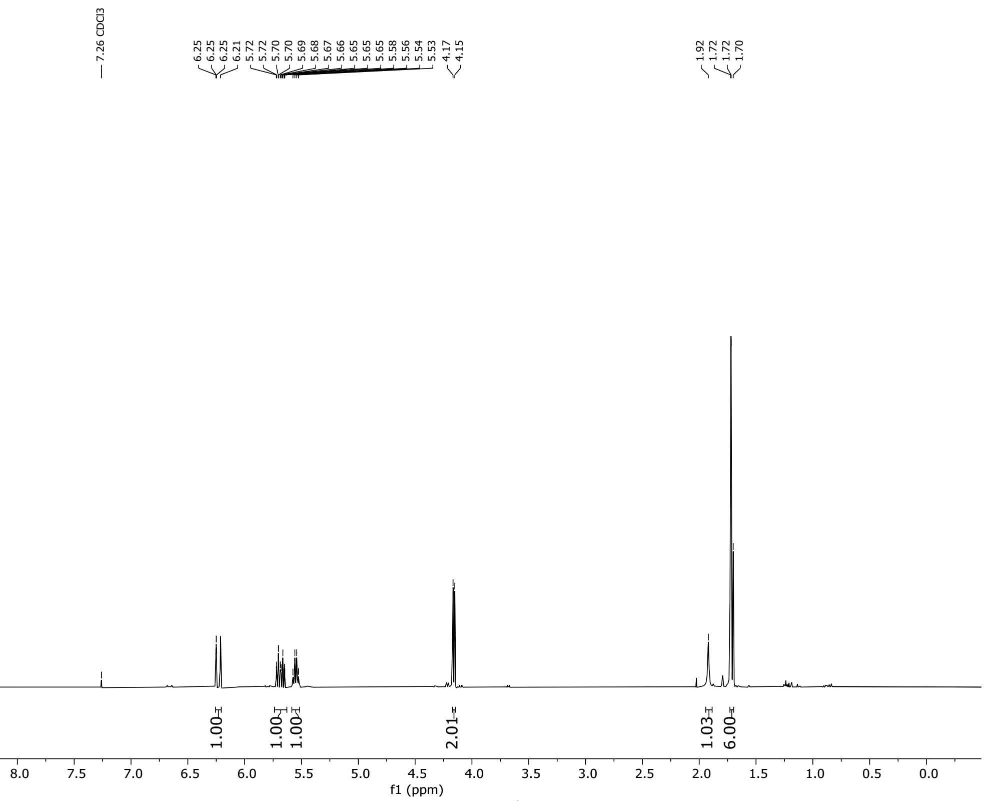

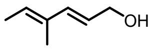

S3
(101 MHz, $CDCl_3$)

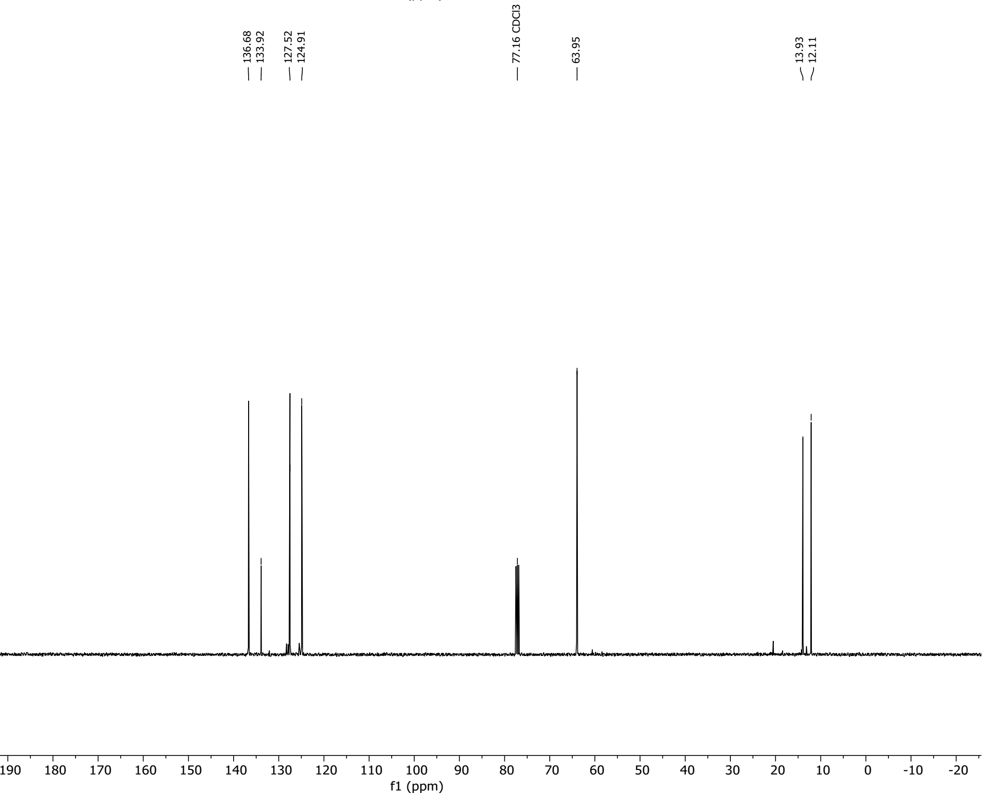

8
(400 MHz, $CDCl_3$)

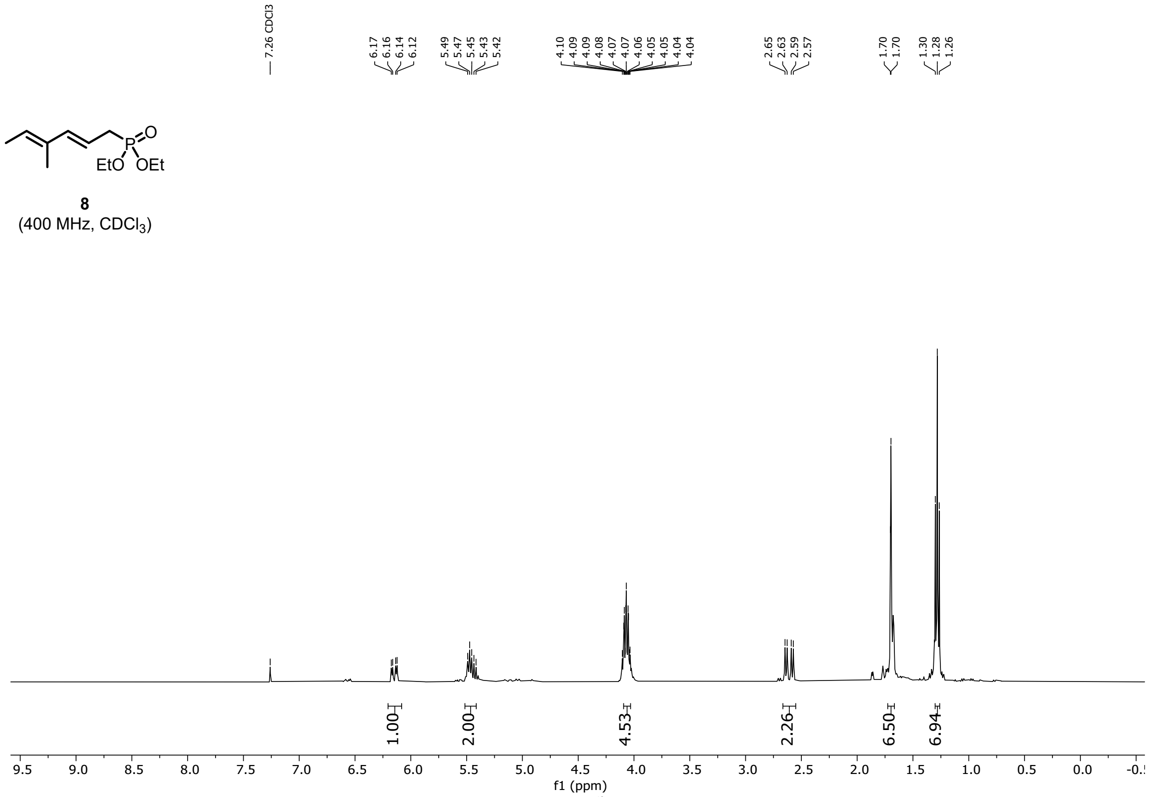

8
(101 MHz, $CDCl_3$)

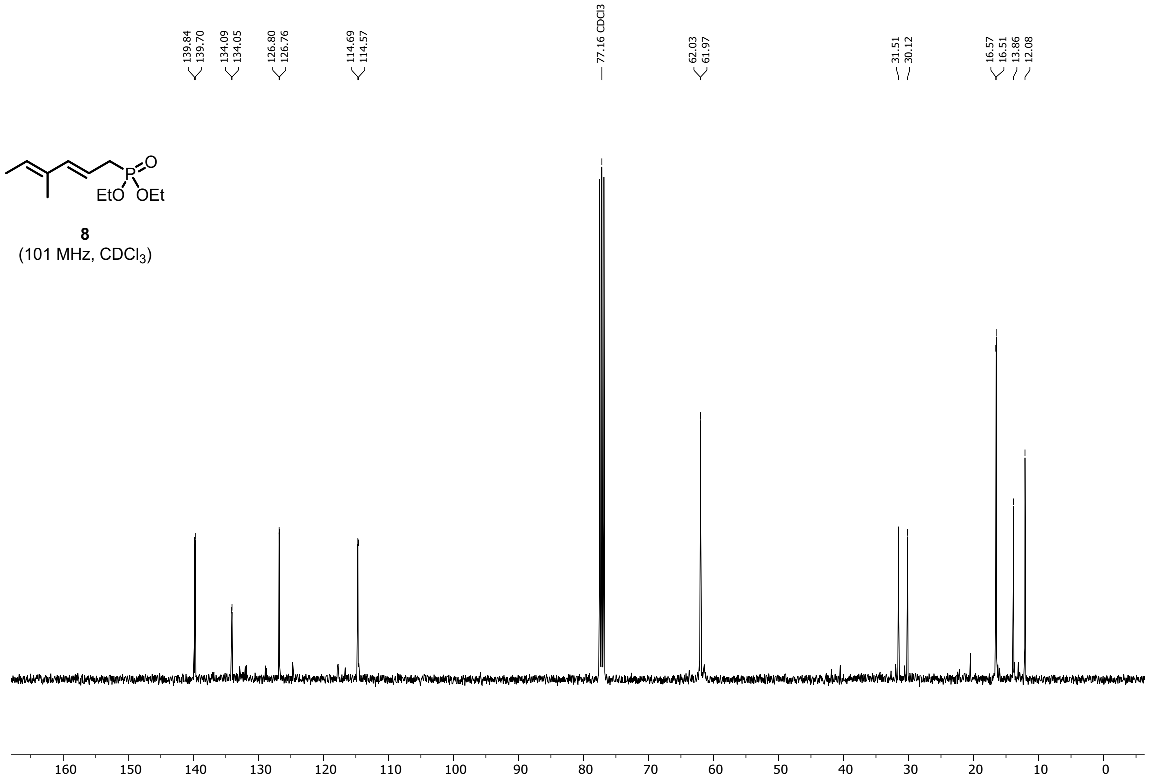

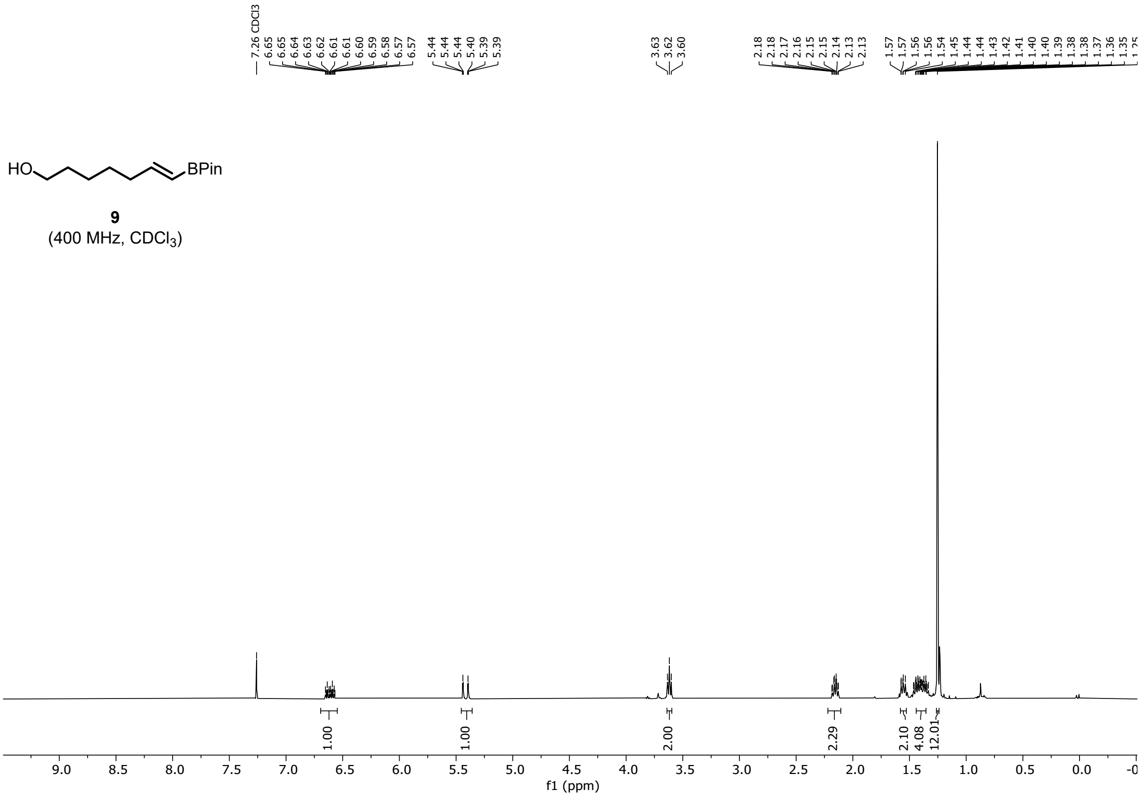
7.26 CDCl3
6.65 6.65 6.64 6.63 6.62 6.61 6.61 6.60 6.59 6.58 6.57 6.57
5.44 5.44 5.44 5.40 5.39 5.39
3.63 3.62 3.60
2.18 2.18 2.17 2.16 2.15 2.15 2.14 2.13 2.13
1.57 1.57 1.56 1.56 1.54 1.45 1.44 1.44 1.43 1.42 1.41 1.40 1.40 1.39 1.38 1.38 1.37 1.36 1.35
HO
BPin
9
(400 MHz, CDCl3)
1.00 1.00 2.00 2.29 2.10 4.08 12.01
9.0 8.5 8.0 7.5 7.0 6.5 6.0 5.5 5.0 4.5 4.0 3.5 3.0 2.5 2.0 1.5 1.0 0.5 0.0
f1 (ppm)

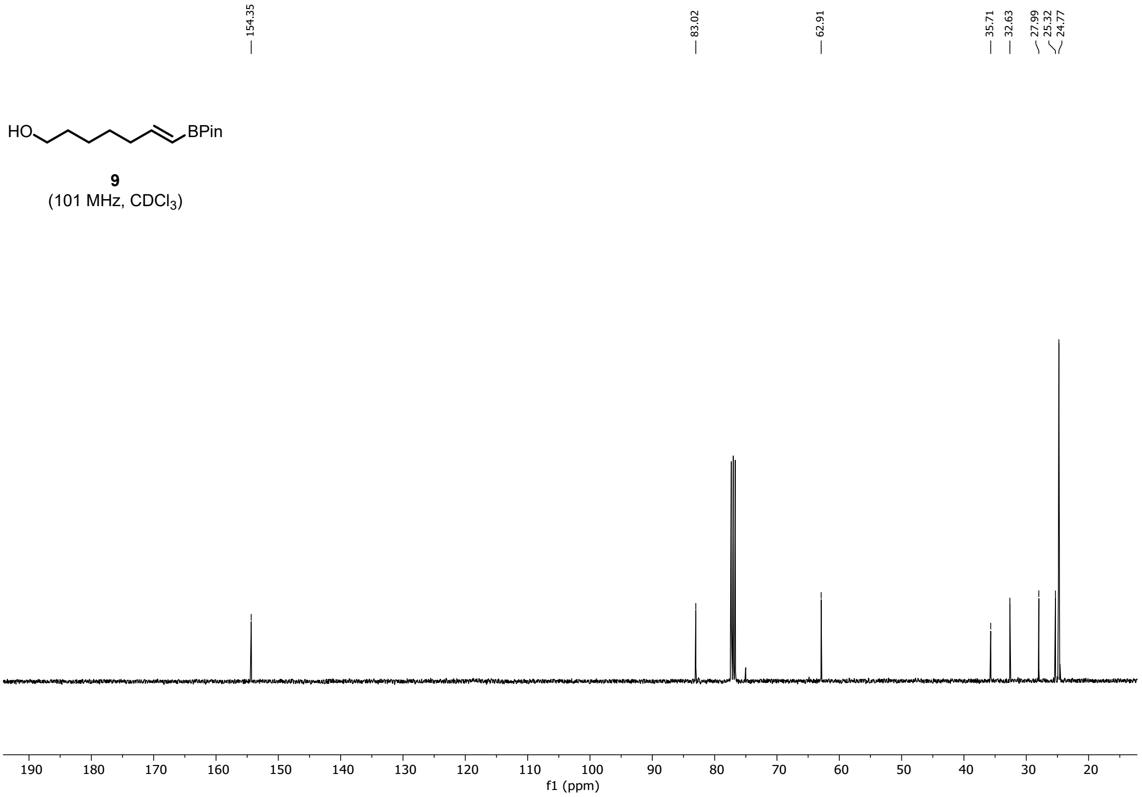
154.35
83.02
62.91
35.71
32.63
27.99
25.32
24.77
HO
BPin
9
(101 MHz, CDCl3)
190 180 170 160 150 140 130 120 110 100 90 80 70 60 50 40 30 20
f1 (ppm)

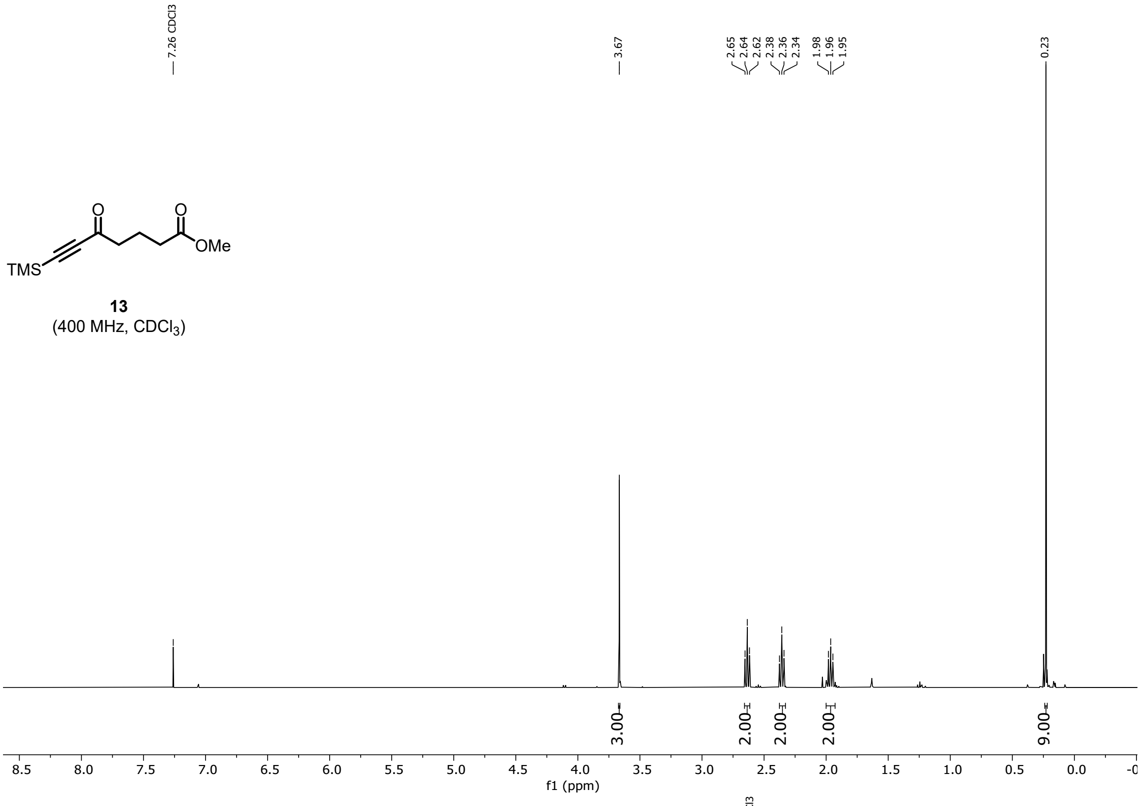
7.26 CDCl3
3.67
2.65
2.64
2.62
2.38
2.36
2.34
1.98
1.96
1.95
0.23
OMe
TMS
13
(400 MHz, CDCl3)
3.00
2.00
2.00
2.00
9.00
f1 (ppm)

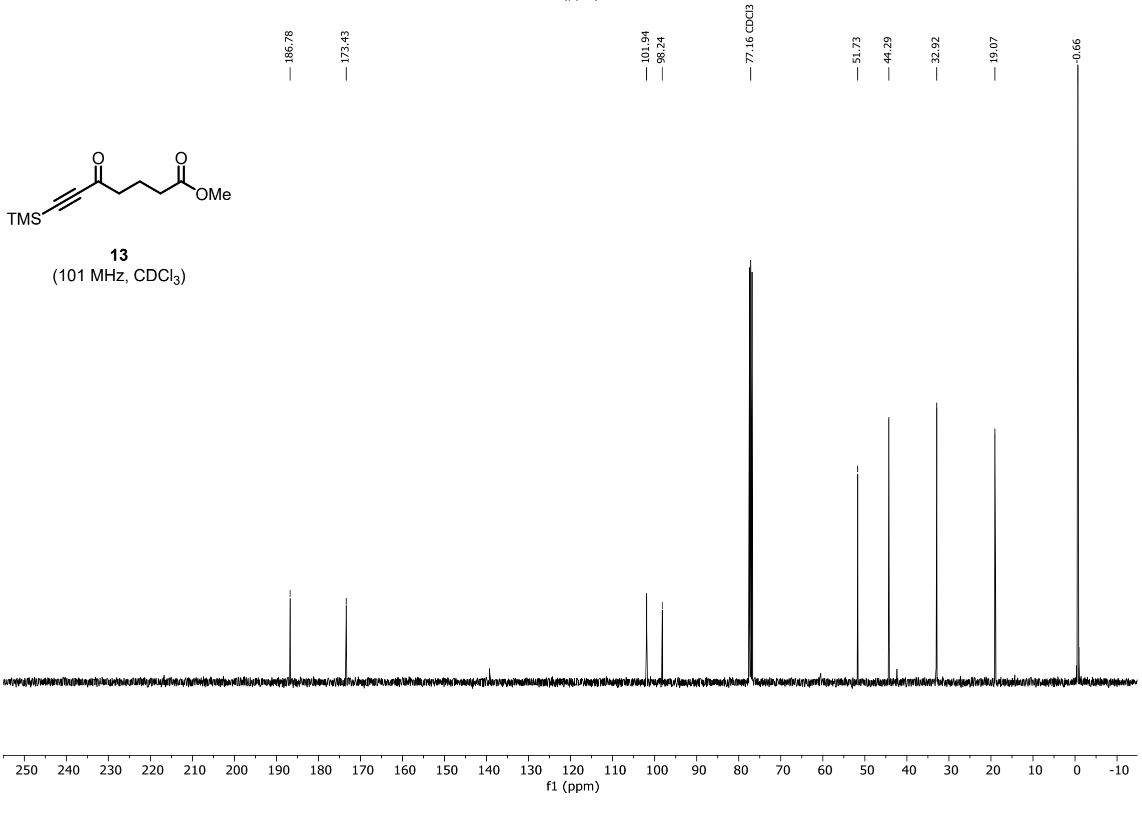
186.78
173.43
101.94
98.24
77.16 CDCl3
51.73
44.29
32.92
19.07
-0.66
OMe
TMS
13
(101 MHz, CDCl3)
f1 (ppm)

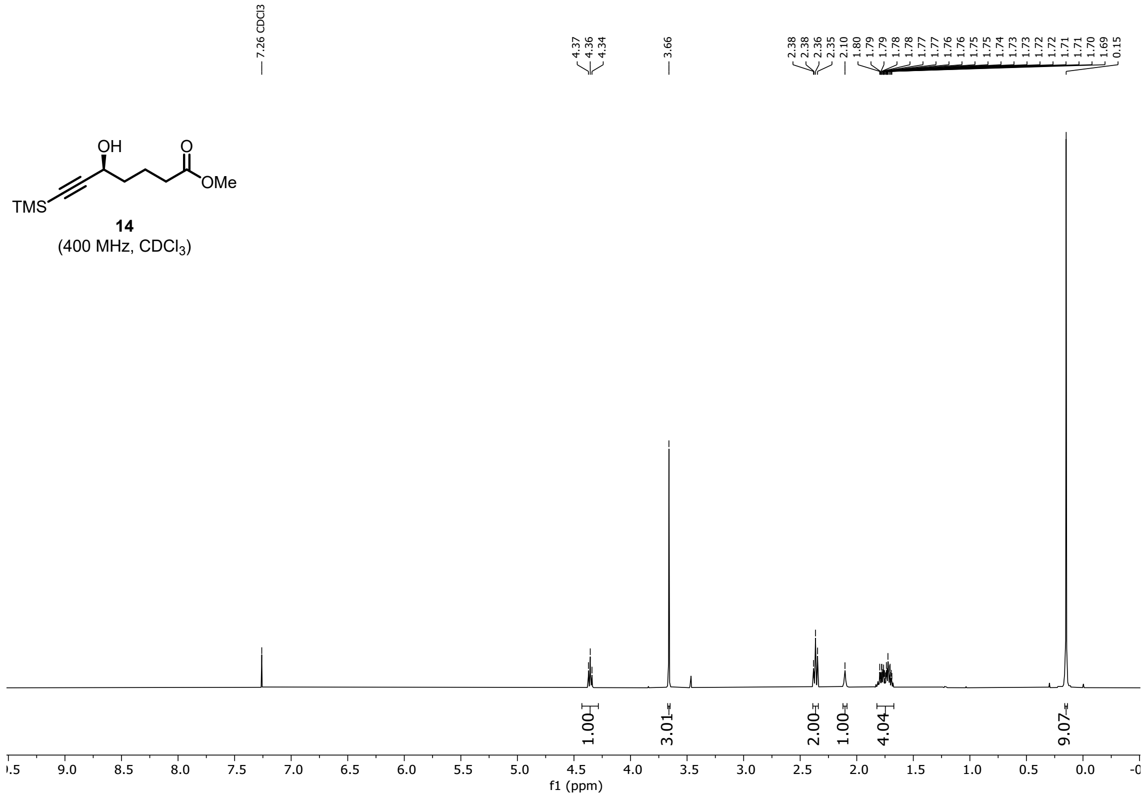

14
(400 MHz, $CDCl_3$)

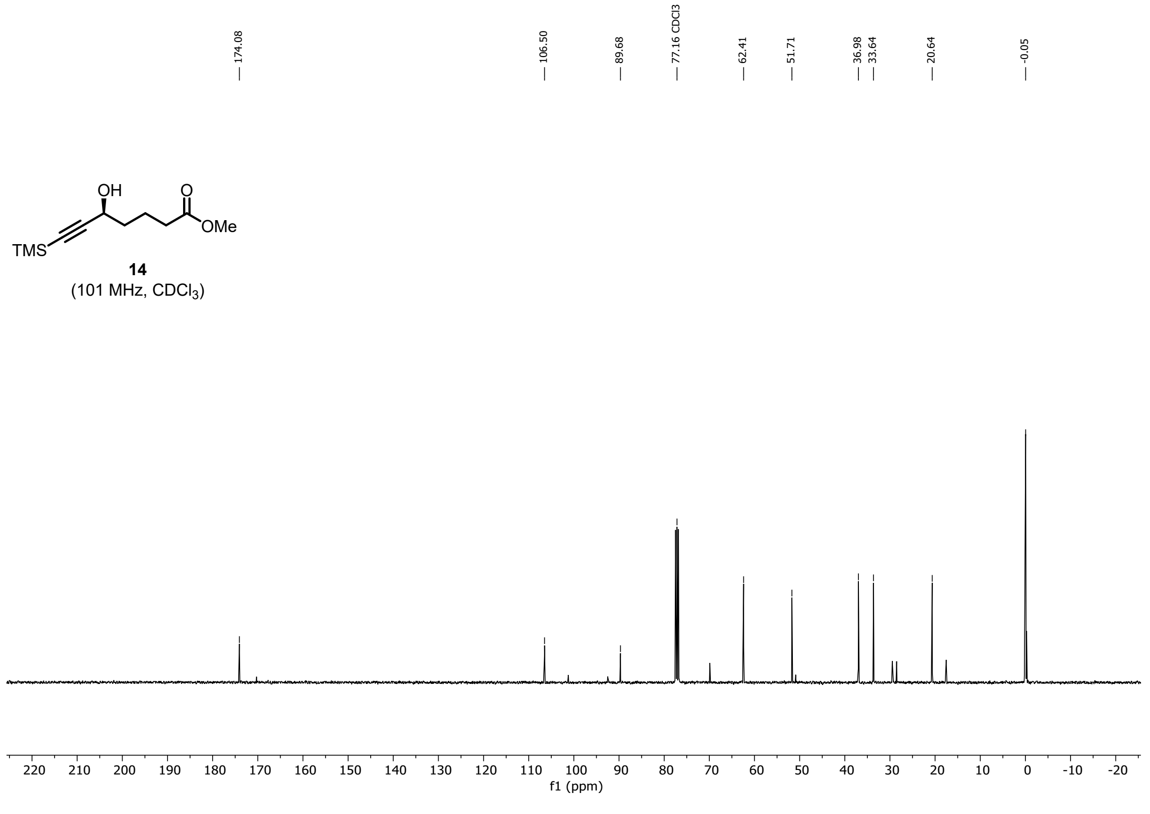

14
(101 MHz, $CDCl_3$)

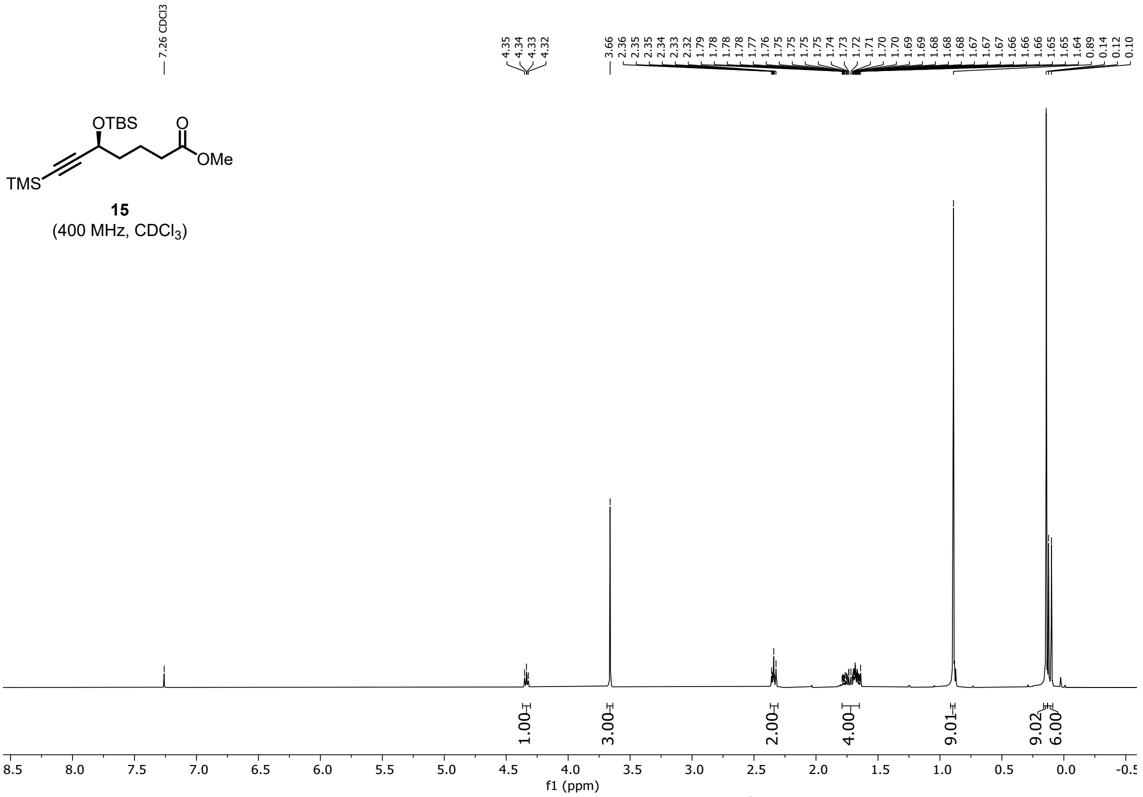

15
(400 MHz, $CDCl_3$)

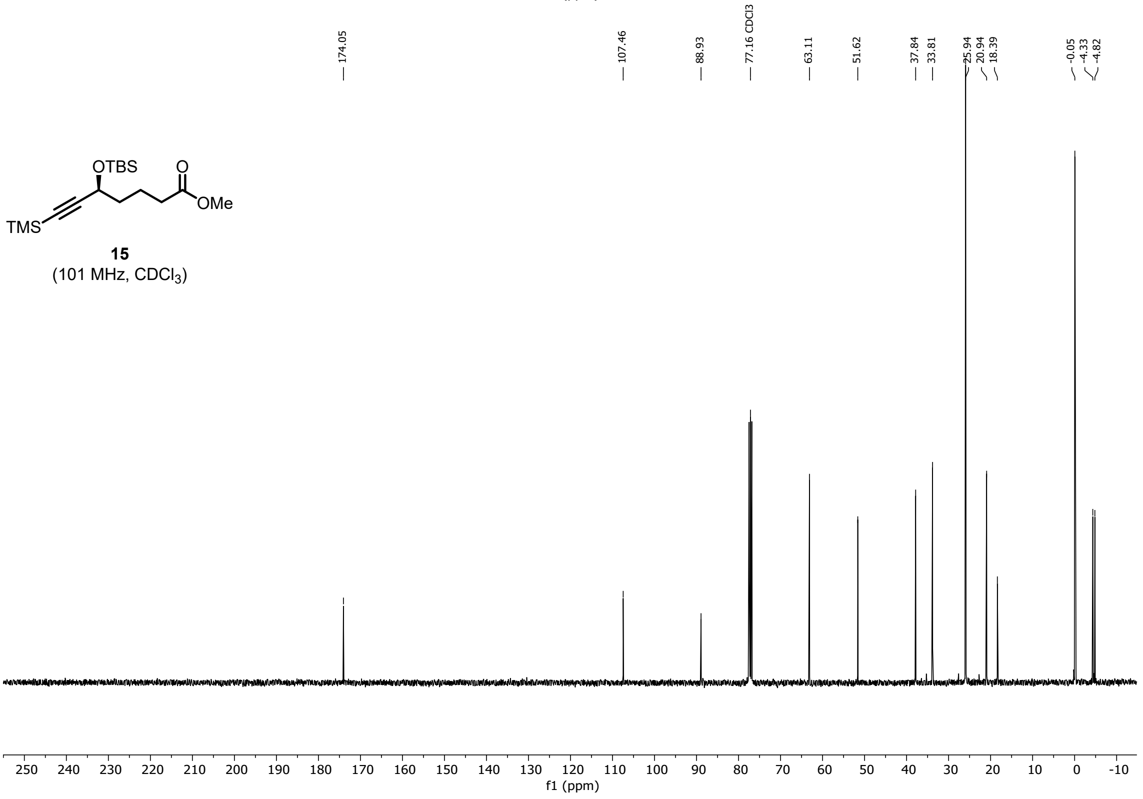

15
(101 MHz, $CDCl_3$)

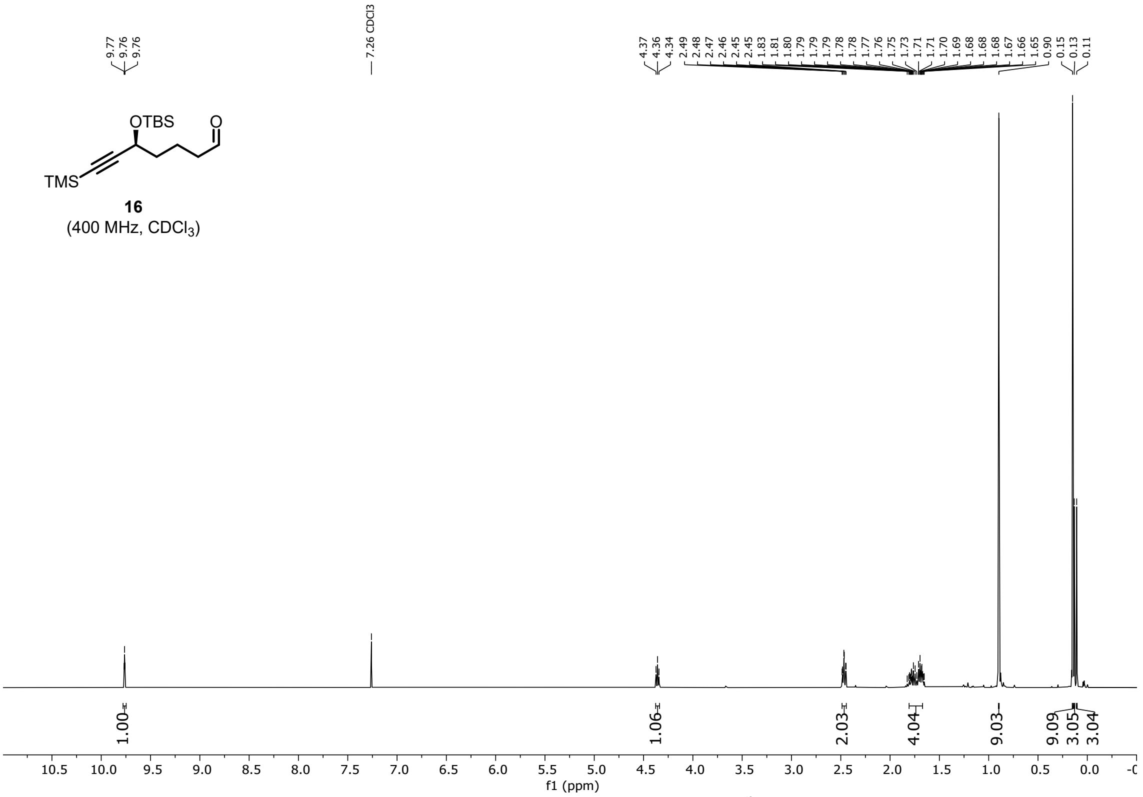
OTBS
O
TMS
16
(400 MHz, CDCl3)
9.77
9.76
9.76
7.26 CDCl3
4.37
4.36
4.34
0.90
0.15
0.13
0.11
1.00
1.06
2.03
4.04
9.03
9.09
3.05
3.04
f1 (ppm)

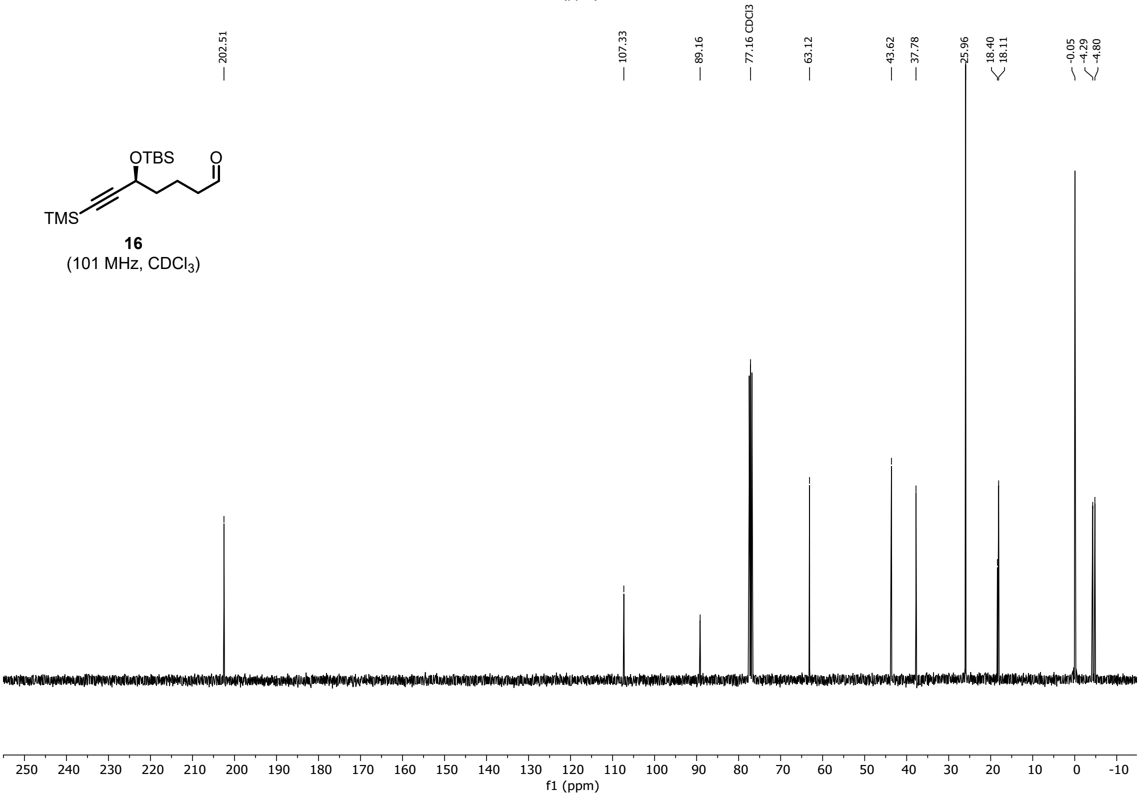
OTBS
O
TMS
16
(101 MHz, CDCl3)
202.51
107.33
89.16
77.16 CDCl3
63.12
43.62
37.78
25.96
18.40
18.11
-0.05
-4.29
-4.80
f1 (ppm)

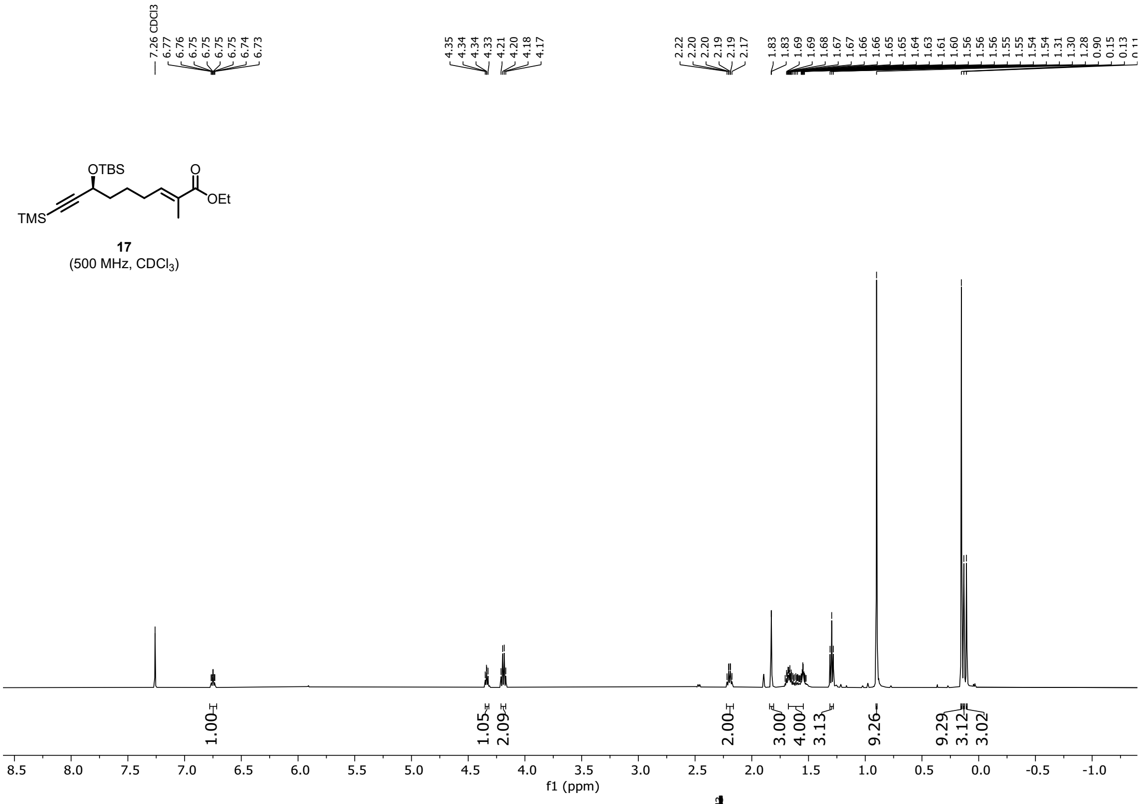
7.26 CDCl3
6.77
6.76
6.75
6.75
6.75
6.74
6.73
4.35
4.34
4.34
4.33
4.21
4.20
4.18
4.17
2.22
2.20
2.20
2.19
2.19
2.17
1.83
1.83
1.69
1.69
1.68
1.67
1.67
1.66
1.65
1.65
1.64
1.63
1.61
1.60
1.56
1.56
1.56
1.55
1.55
1.54
1.54
1.31
1.30
1.28
0.90
0.15
0.13
0.11
OTBS
O
OEt
TMS
17
(500 MHz, $CDCl_3$)
1.00
1.05
2.09
2.00
3.00
4.00
3.13
9.26
9.29
3.12
3.02
f1 (ppm)

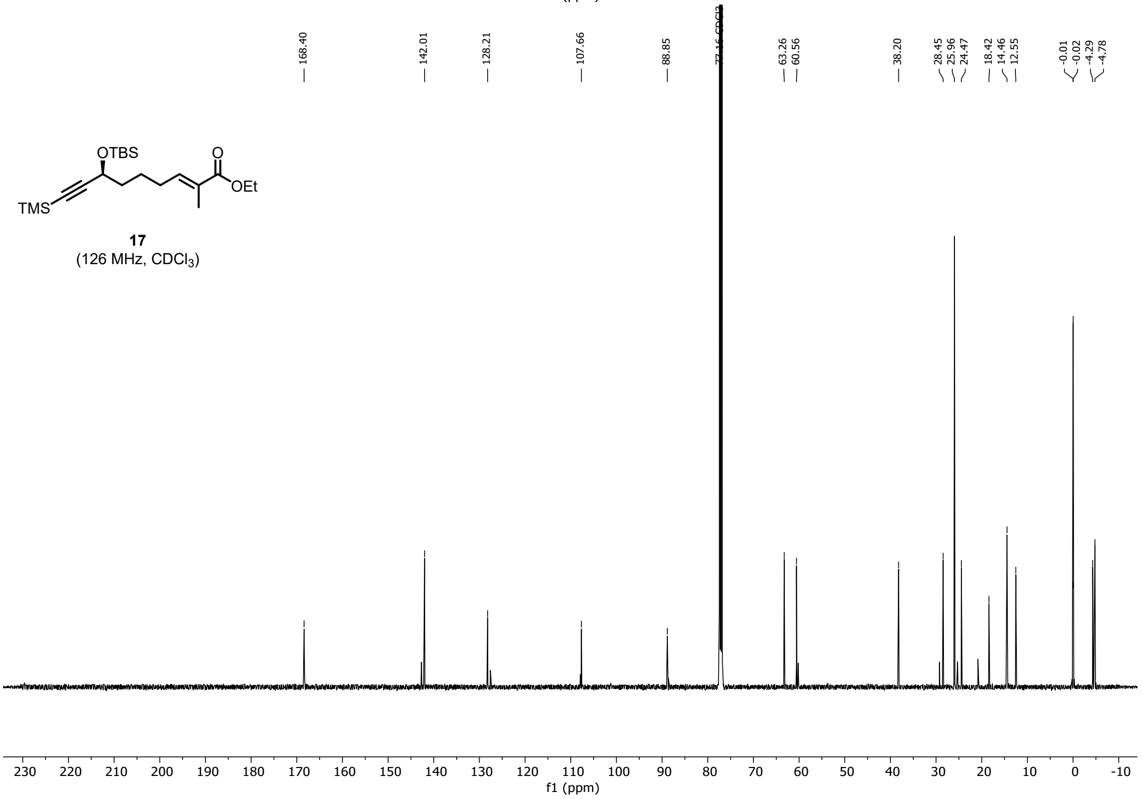
168.40
142.01
128.21
107.66
88.85
77.16 CDCl3
63.26
60.56
38.20
28.45
25.96
24.47
18.42
14.46
12.55
-0.01
-0.02
-4.29
-4.78
OTBS
O
OEt
TMS
17
(126 MHz, $CDCl_3$)
f1 (ppm)

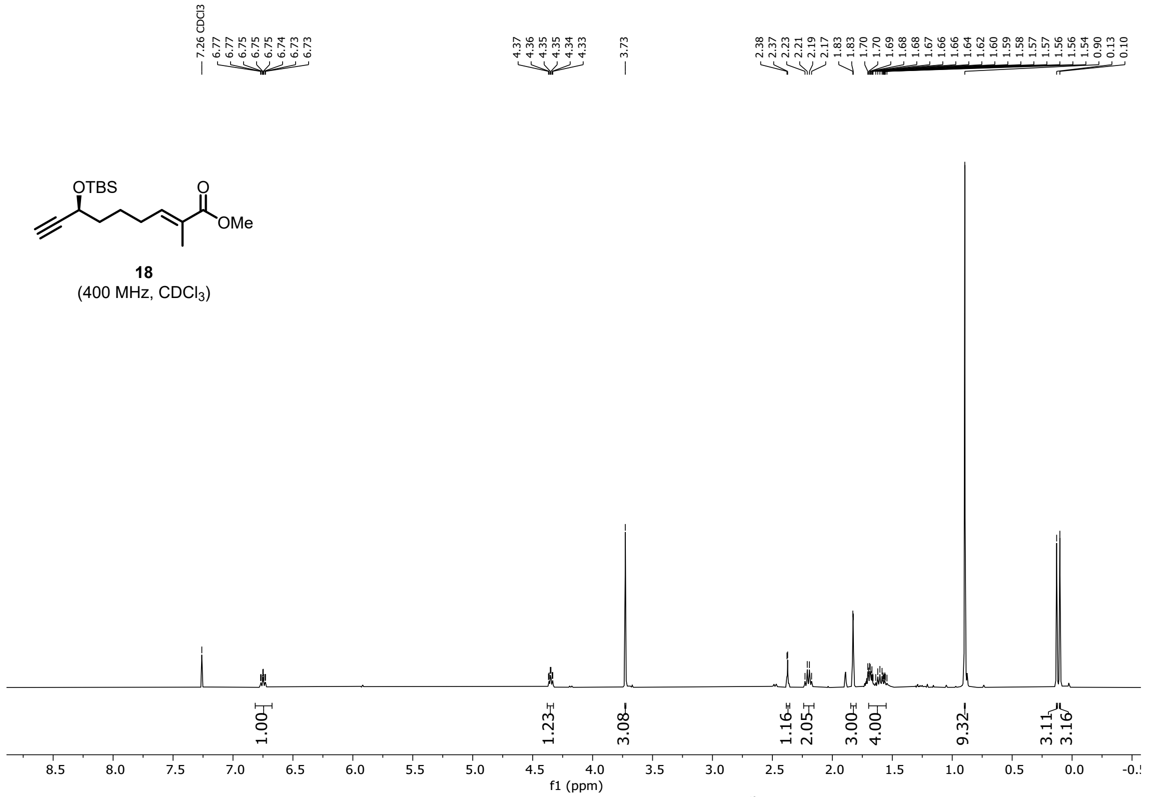
OTBS
O
OMe
18
(400 MHz, CDCl$_3$)
f1 (ppm)

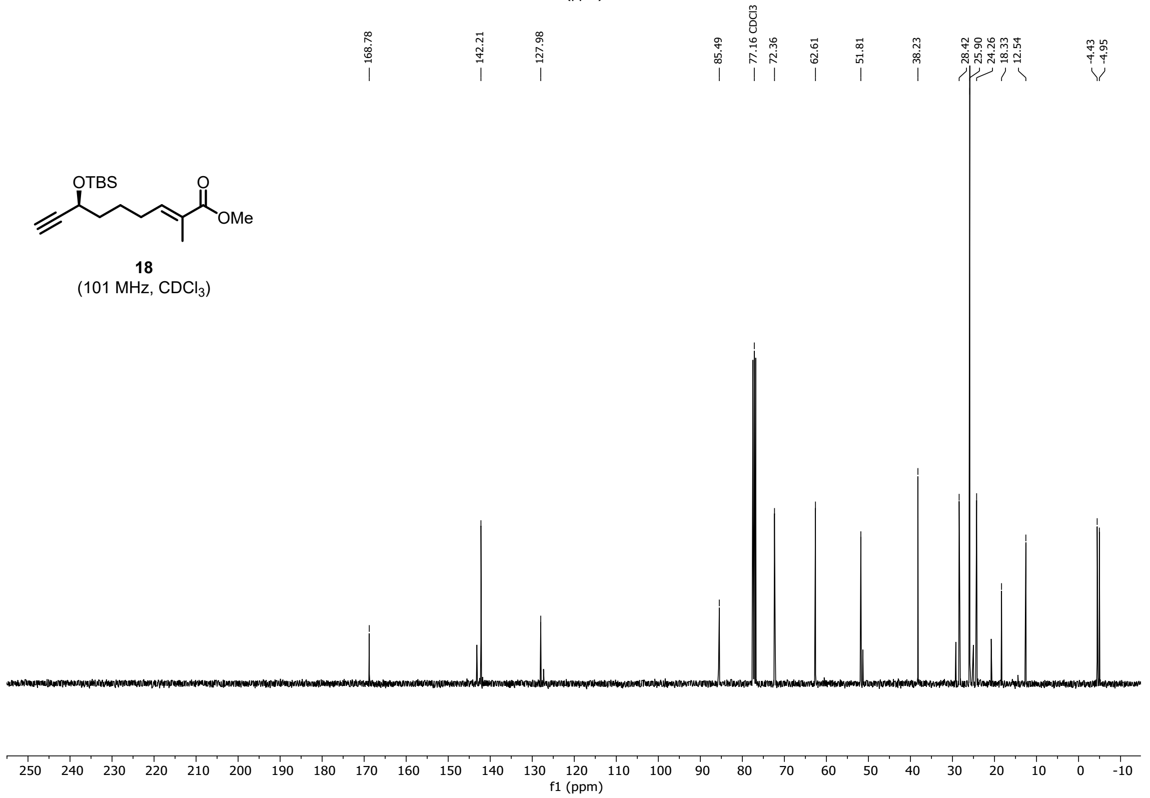
OTBS
O
OMe
18
(101 MHz, CDCl$_3$)
f1 (ppm)

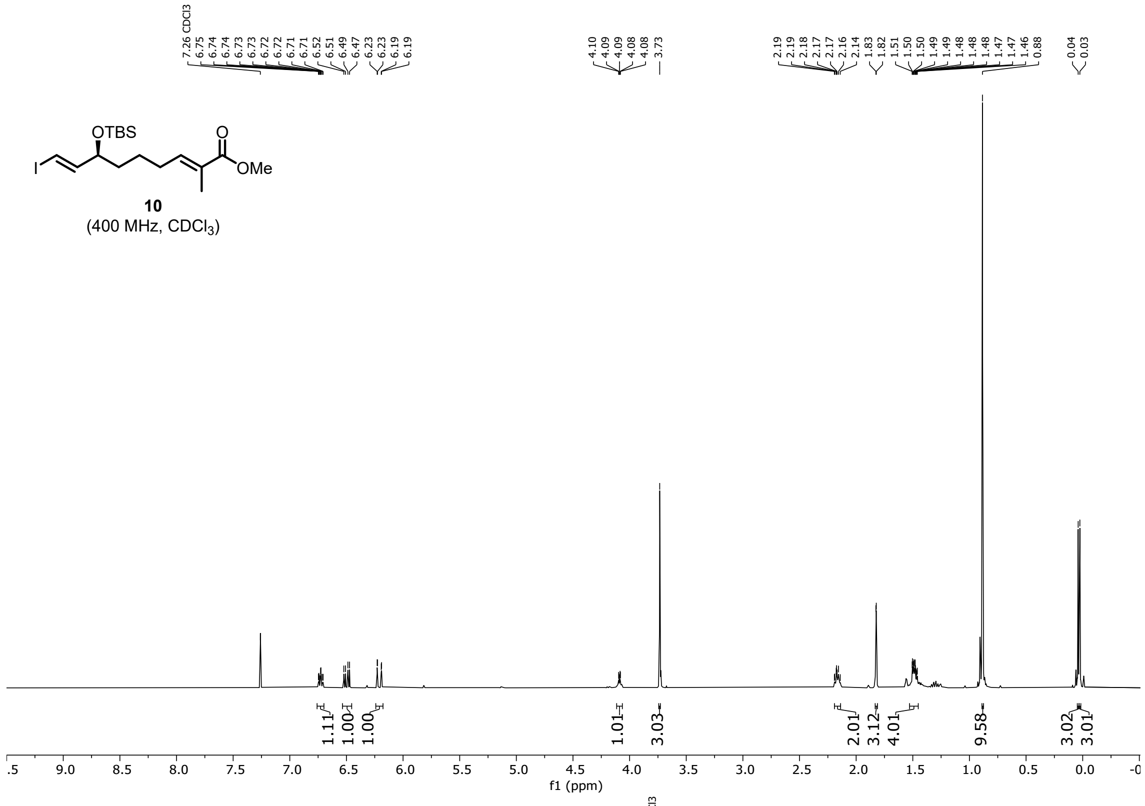
OTBS
I
O
OMe
10
(400 MHz, CDCl3)
7.26 CDCl3
6.75
6.74
6.74
6.73
6.73
6.72
6.72
6.71
6.71
6.52
6.51
6.49
6.47
6.23
6.23
6.19
6.19
4.10
4.09
4.09
4.08
4.08
3.73
2.19
2.19
2.18
2.17
2.17
2.16
2.14
1.83
1.82
1.51
1.50
1.50
1.49
1.49
1.48
1.48
1.48
1.47
1.47
1.46
0.88
0.04
0.03
1.11
1.00
1.00
1.01
3.03
2.01
3.12
4.01
9.58
3.02
3.01
f1 (ppm)

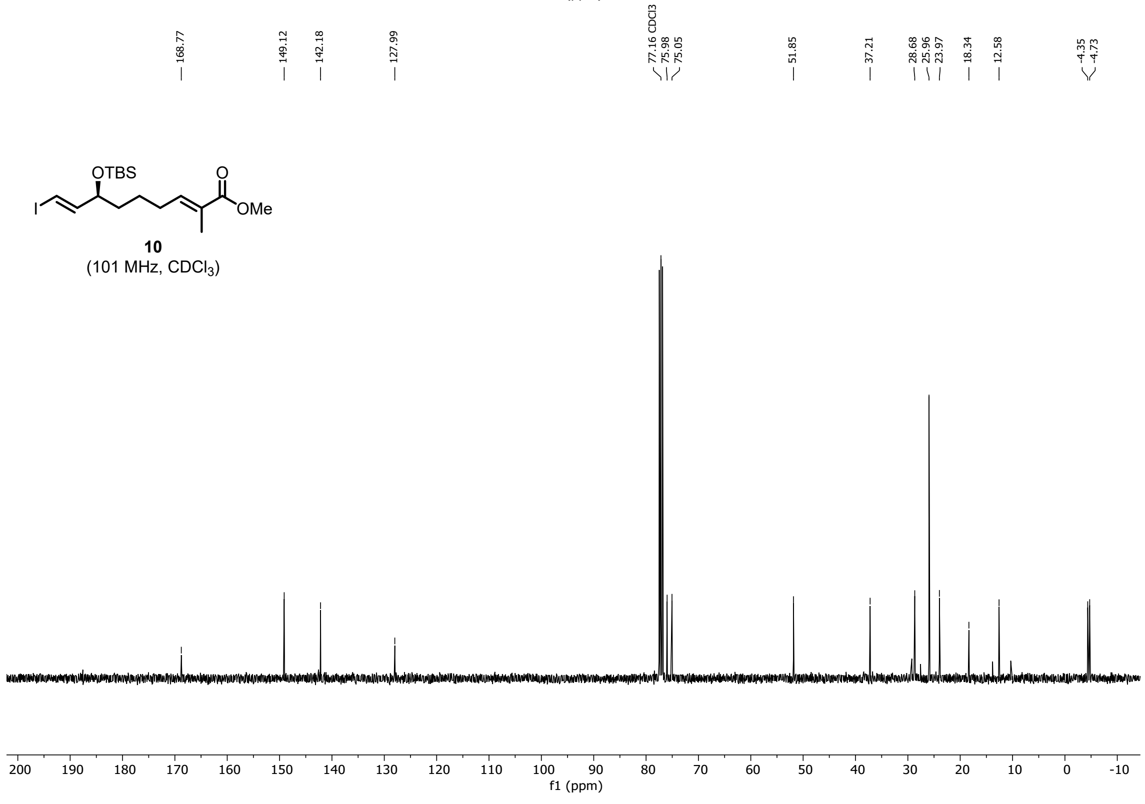
OTBS
I
O
OMe
10
(101 MHz, CDCl3)
168.77
149.12
142.18
127.99
77.16 CDCl3
75.98
75.05
51.85
37.21
28.68
25.96
23.97
18.34
12.58
-4.35
-4.73
f1 (ppm)

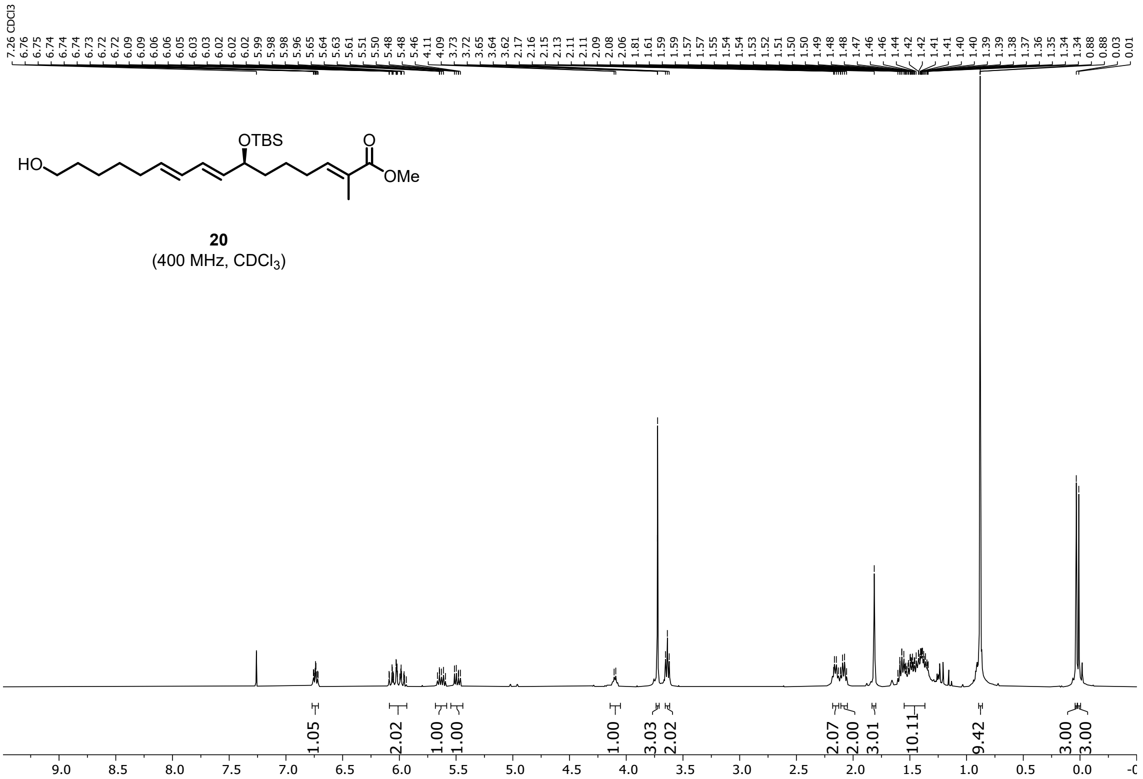
7.26 CDCl3
6.76
6.75
6.74
6.74
6.74
6.73
6.72
6.72
6.09
6.09
6.06
6.06
6.05
6.03
6.03
6.02
6.02
5.99
5.98
5.98
5.96
5.65
5.64
5.63
5.61
5.51
5.50
5.48
5.48
5.46
4.11
4.09
3.73
3.72
3.65
3.64
3.62
2.17
2.16
2.15
2.13
2.11
2.11
2.09
2.08
2.06
1.81
1.61
1.59
1.59
1.57
1.57
1.55
1.54
1.54
1.53
1.52
1.51
1.50
1.50
1.49
1.48
1.48
1.47
1.46
1.46
1.44
1.42
1.42
1.41
1.41
1.40
1.39
1.39
1.38
1.37
1.36
1.35
1.34
1.34
0.88
0.03
0.01
HO
OTBS
O
OMe
20
(400 MHz, CDCl3)
1.05
2.02
1.00
1.00
1.00
3.03
2.02
2.07
2.00
3.01
10.11
9.42
3.00
3.00
f1 (ppm)

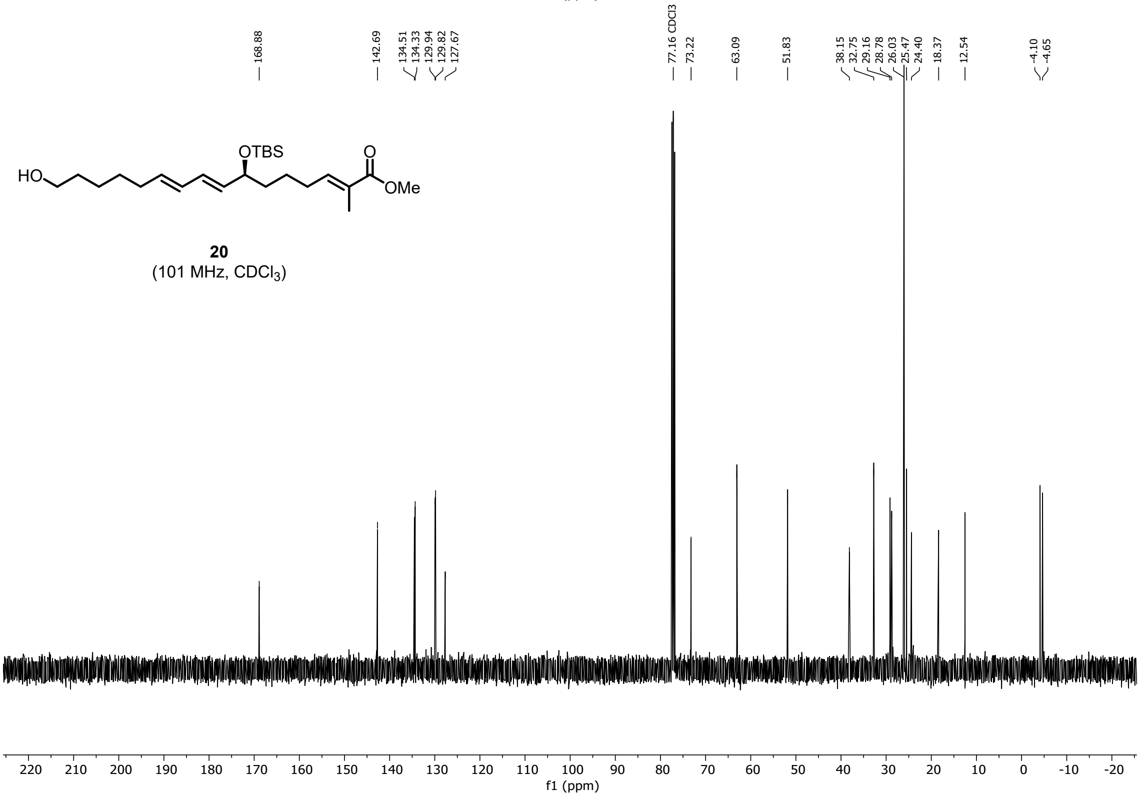
168.88
142.69
134.51
134.33
129.94
129.82
127.67
77.16 CDCl3
73.22
63.09
51.83
38.15
32.75
29.16
28.78
26.03
25.47
24.40
18.37
12.54
-4.10
-4.65
HO
OTBS
O
OMe
20
(101 MHz, CDCl3)
f1 (ppm)

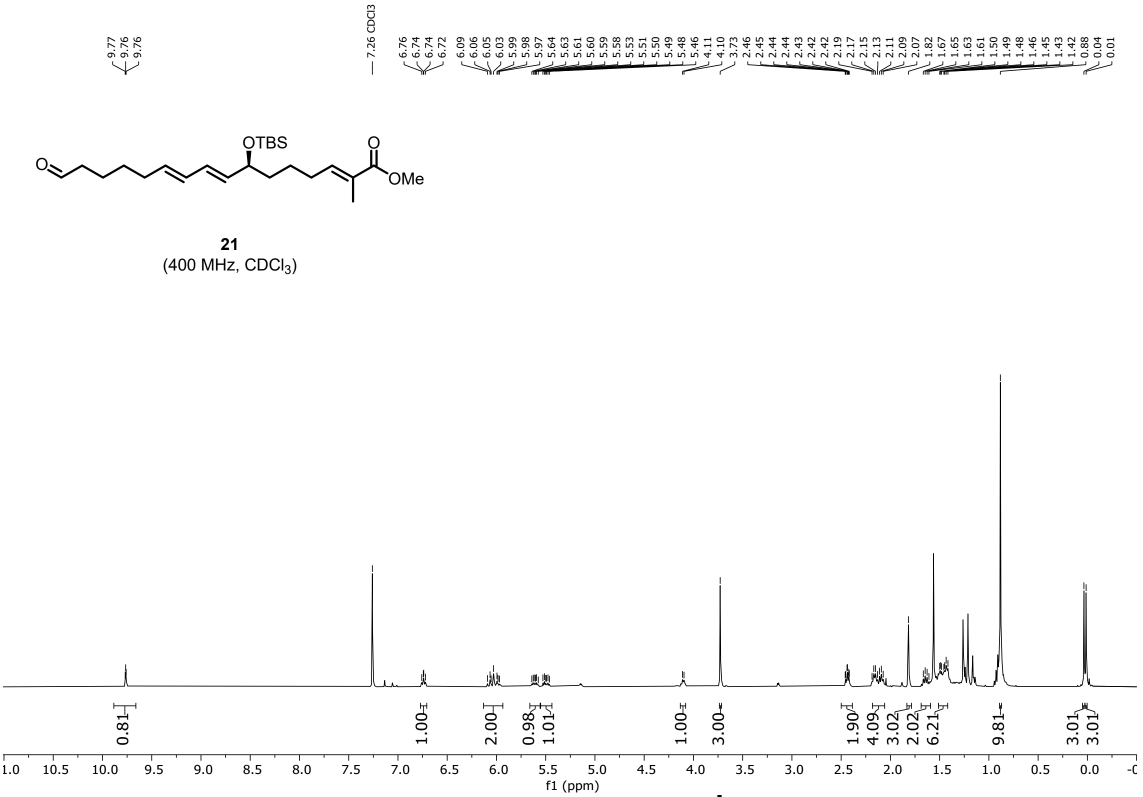

21
(400 MHz, $CDCl_3$)

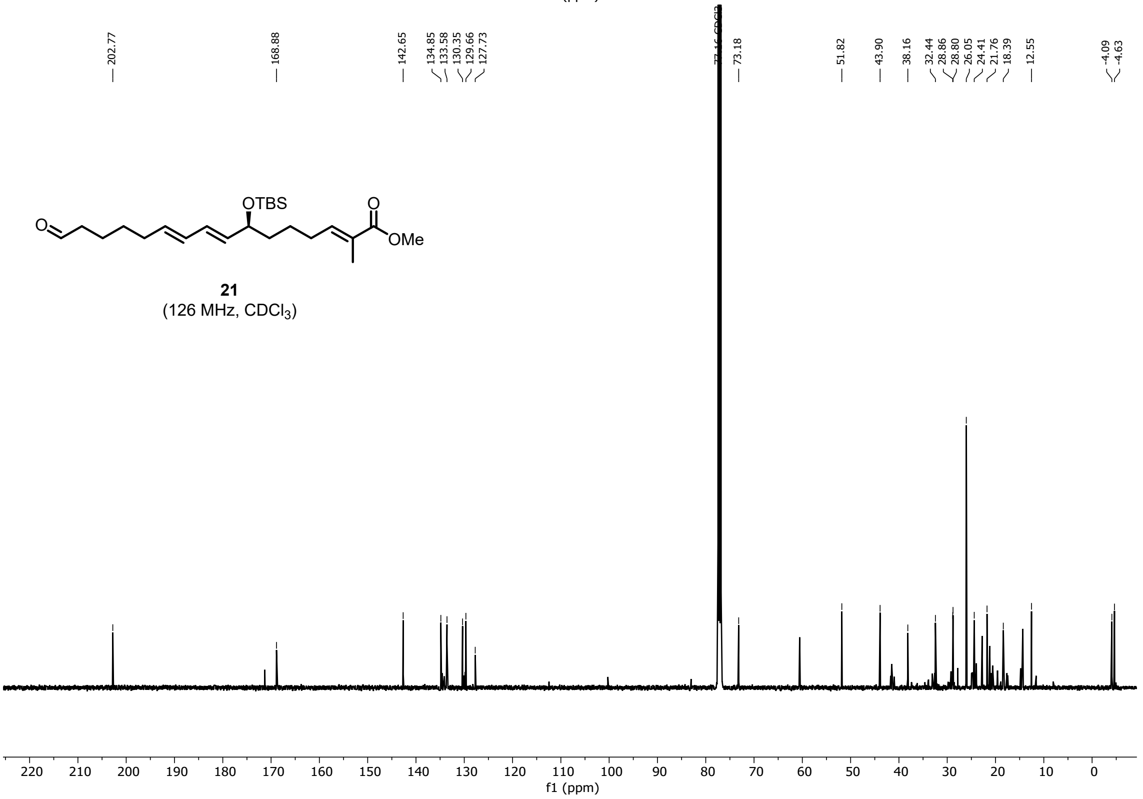

21
(126 MHz, $CDCl_3$)

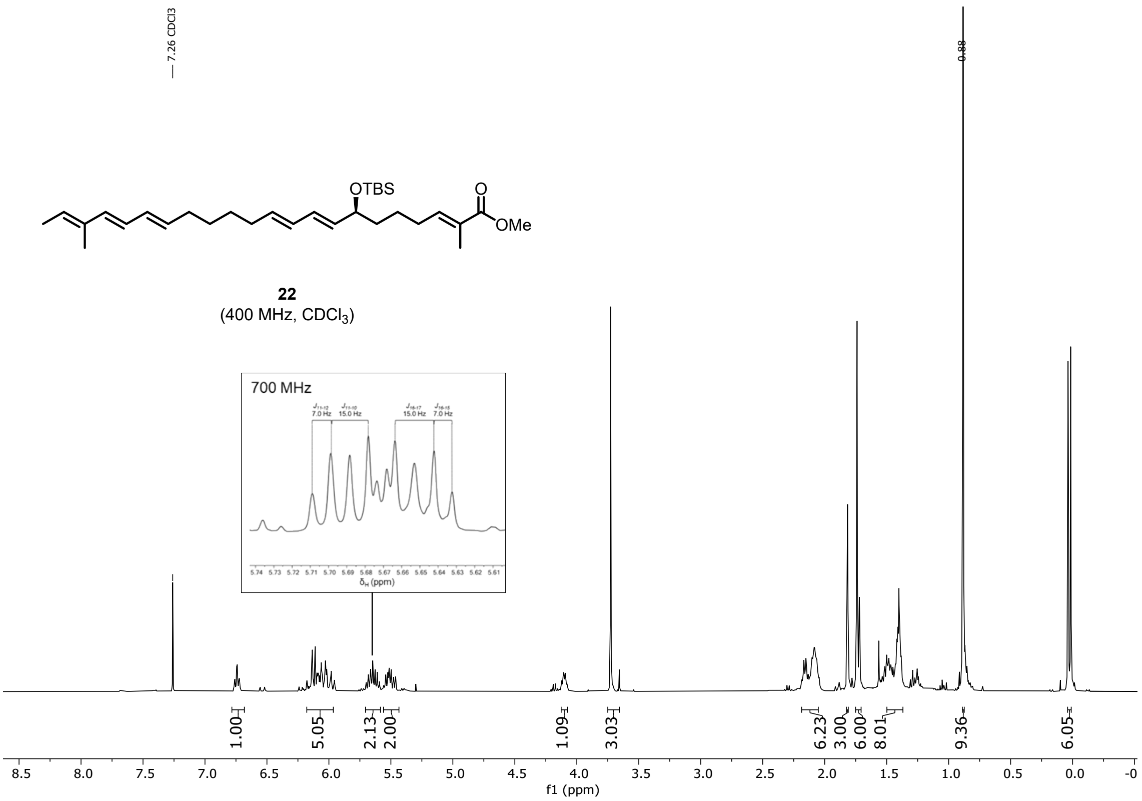
7.26 CDCl3
0.88
OTBS
O
OMe
22
(400 MHz, CDCl3)
700 MHz
δH (ppm)
1.00
5.05
2.13
2.00
1.09
3.03
6.23
3.00
6.00
8.01
9.36
6.05
f1 (ppm)

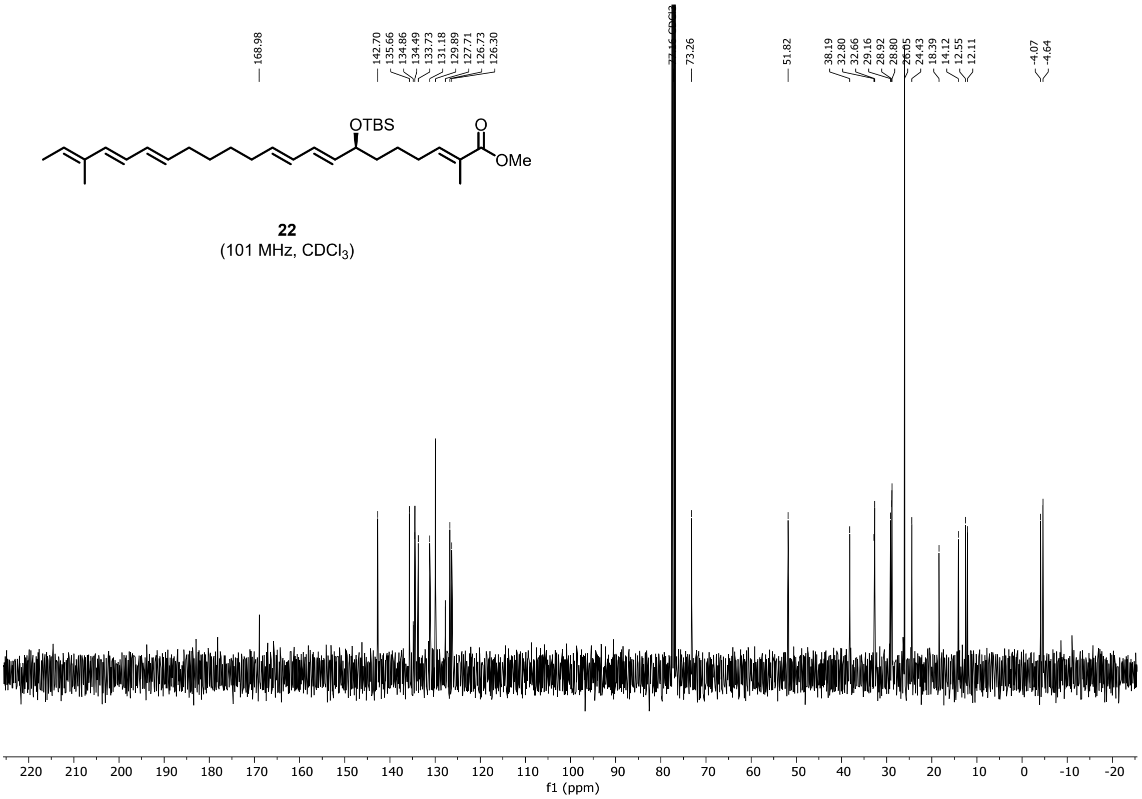
168.98
142.70
135.66
134.86
134.49
133.73
131.18
129.89
127.71
126.73
126.30
77.16 CDCl3
73.26
51.82
38.19
32.80
32.66
29.16
28.92
28.80
26.05
24.43
18.39
14.12
12.55
12.11
-4.07
-4.64
OTBS
O
OMe
22
(101 MHz, CDCl3)
f1 (ppm)

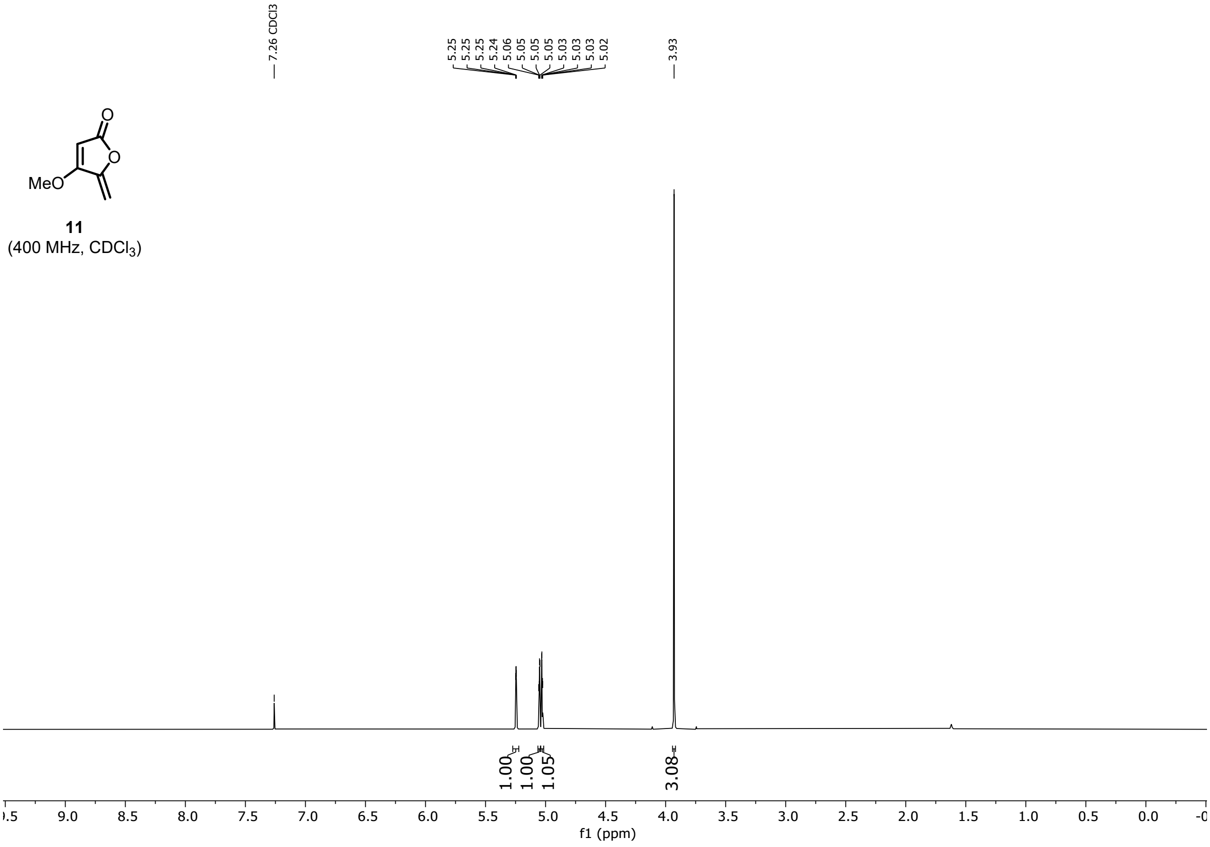
11
(400 MHz, $CDCl_3$)
7.26 CDCl3
5.25
5.25
5.25
5.24
5.06
5.05
5.05
5.05
5.03
5.03
5.03
5.02
3.93
1.00
1.00
1.05
3.08
f1 (ppm)

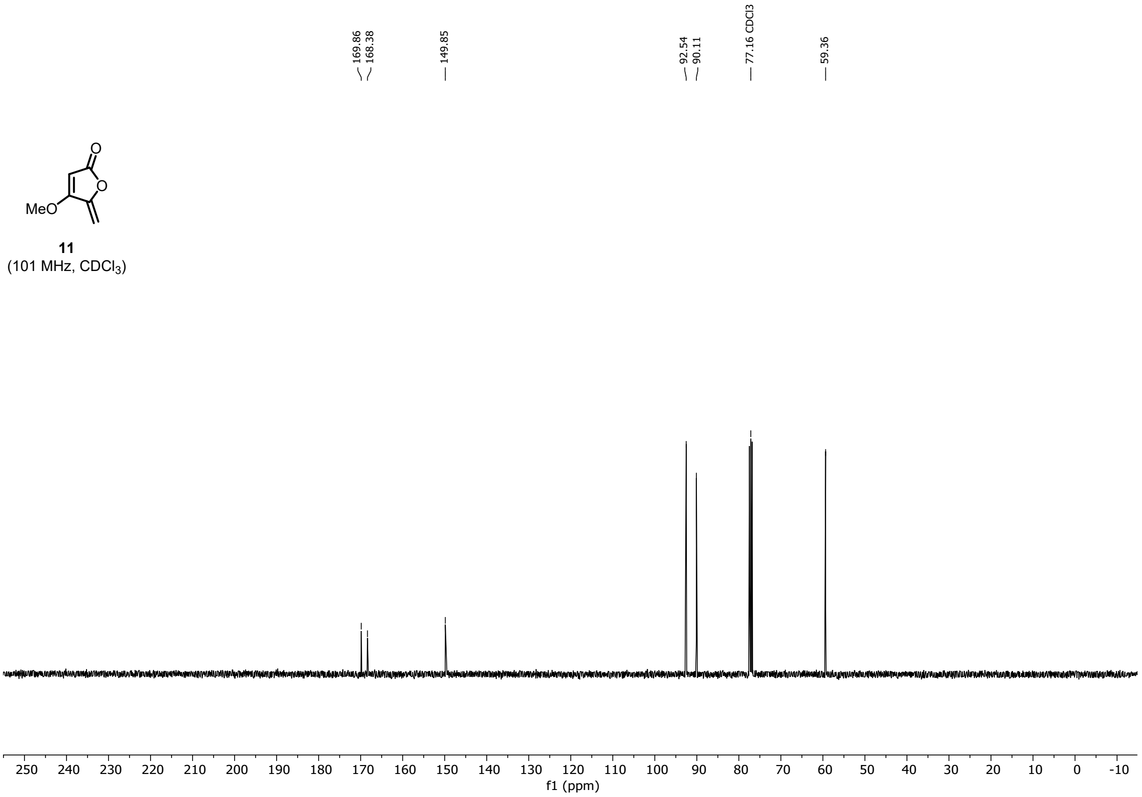
11
(101 MHz, $CDCl_3$)
169.86
168.38
149.85
92.54
90.11
77.16 CDCl3
59.36
f1 (ppm)

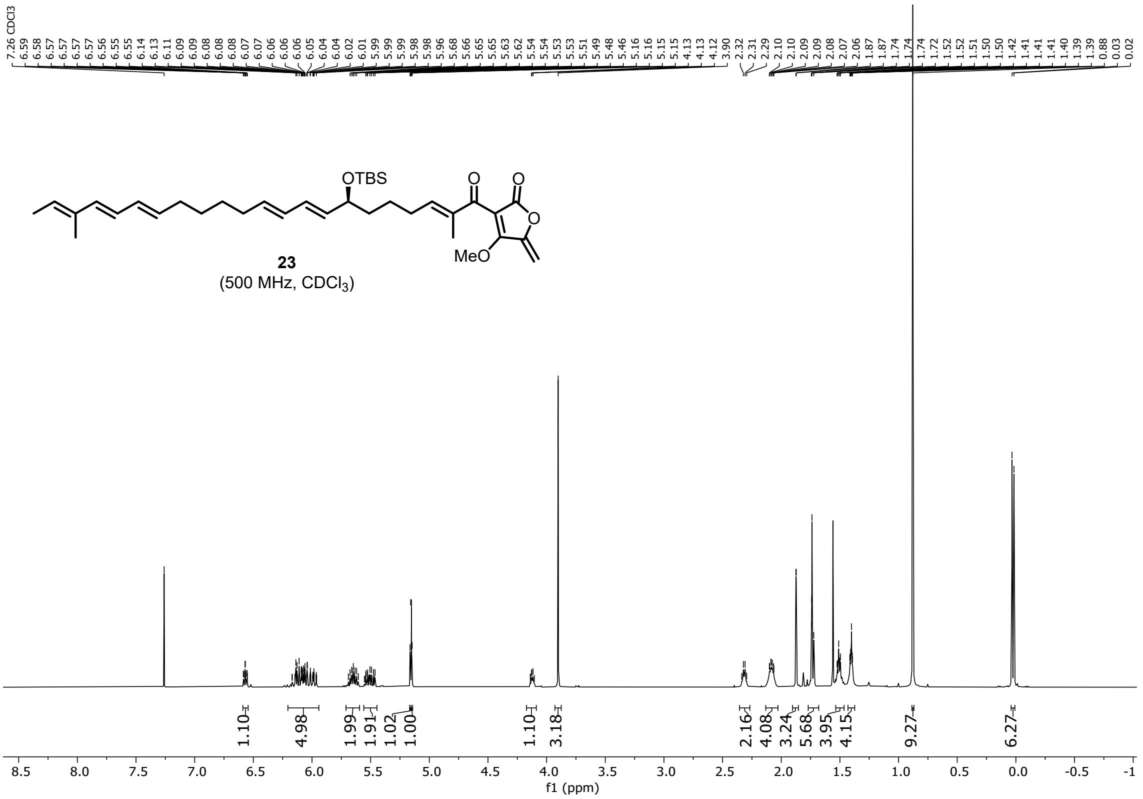
OTBS
MeO
23
(500 MHz, CDCl3)
f1 (ppm)

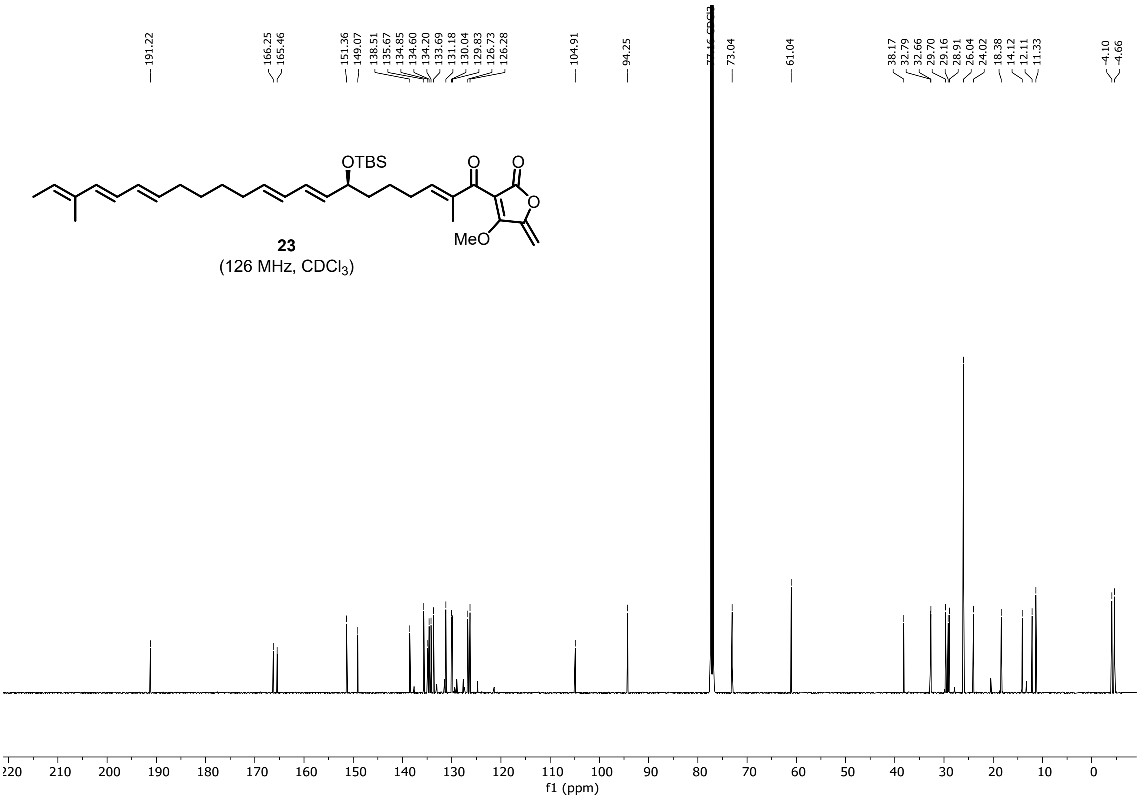
OTBS
MeO
23
(126 MHz, CDCl3)
f1 (ppm)

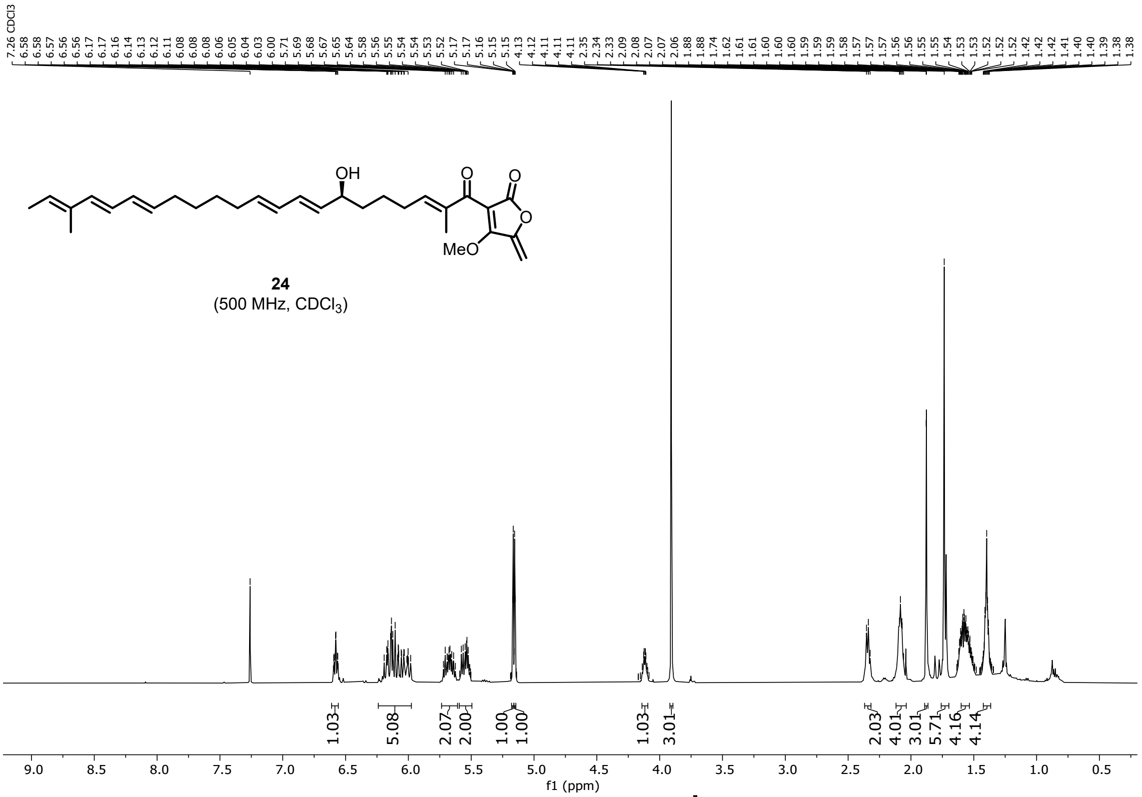

24
(500 MHz, $CDCl_3$)

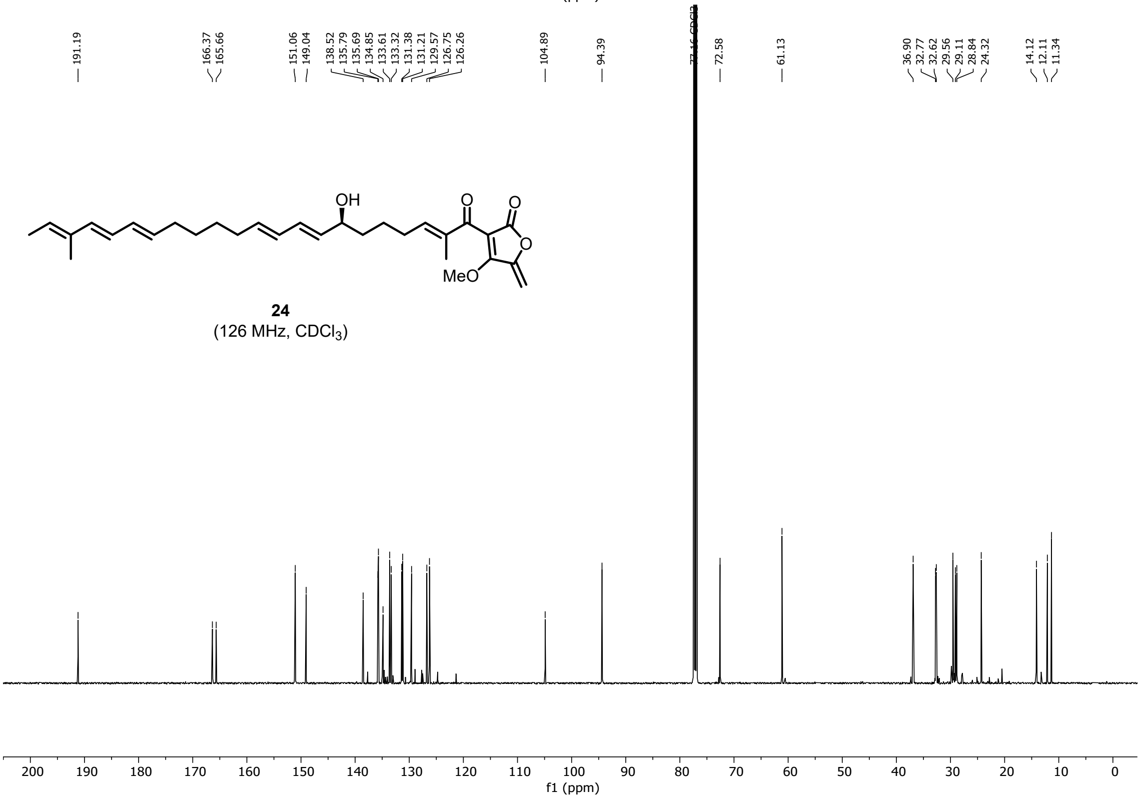

24
(126 MHz, $CDCl_3$)

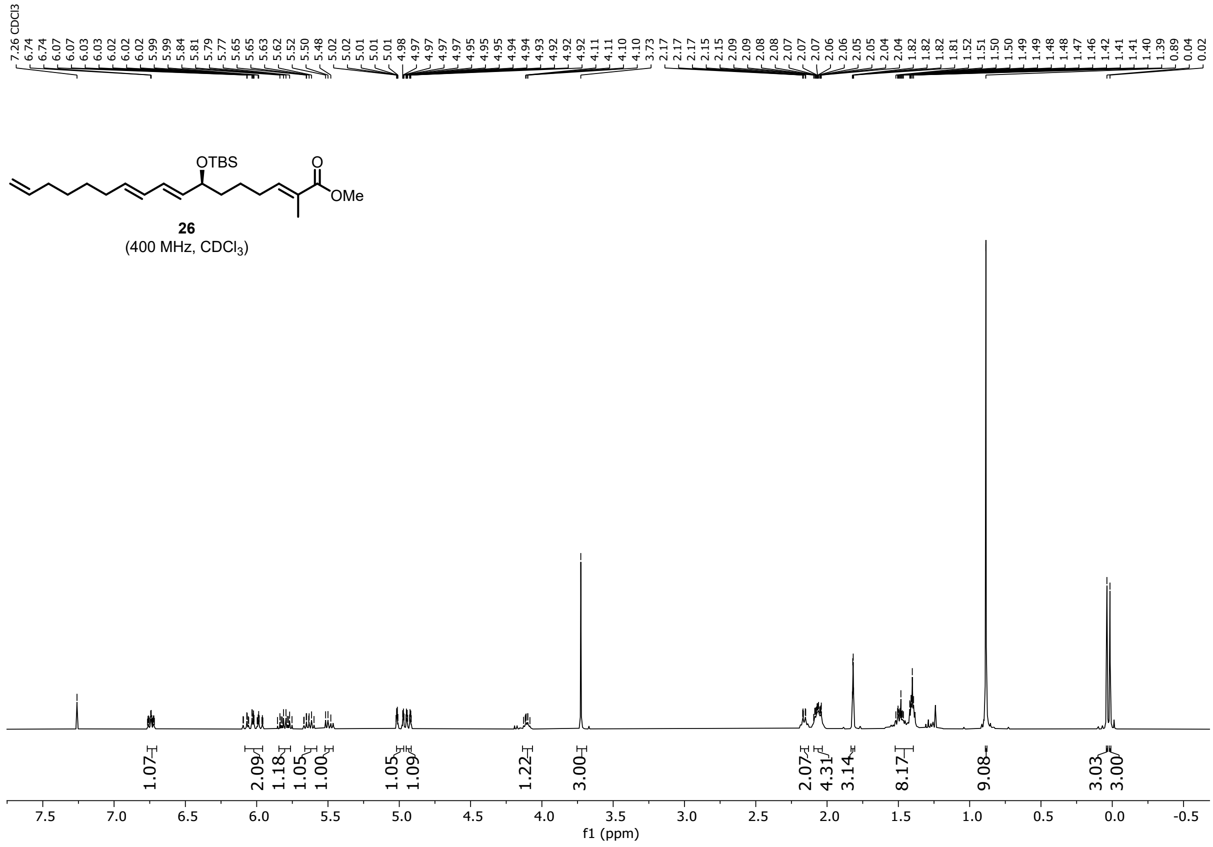
7.26 CDCl3
6.74
6.74
6.07
6.07
6.03
6.03
6.02
6.02
6.02
5.99
5.99
5.84
5.81
5.79
5.77
5.65
5.65
5.63
5.62
5.52
5.50
5.48
5.02
5.02
5.01
5.01
5.01
4.98
4.97
4.97
4.97
4.97
4.95
4.95
4.95
4.94
4.93
4.92
4.92
4.92
4.92
4.11
4.11
4.10
4.10
3.73
2.17
2.17
2.17
2.15
2.15
2.09
2.09
2.08
2.08
2.07
2.07
2.07
2.06
2.06
2.05
2.05
2.04
2.04
1.82
1.82
1.82
1.81
1.52
1.51
1.50
1.50
1.49
1.49
1.48
1.47
1.46
1.42
1.41
1.41
1.40
1.39
0.89
0.04
0.02
OTBS
O
OMe
26
(400 MHz, CDCl3)
1.07
2.09
1.18
1.05
1.00
1.05
1.09
1.22
3.00
2.07
4.31
3.14
8.17
9.08
3.03
3.00
f1 (ppm)

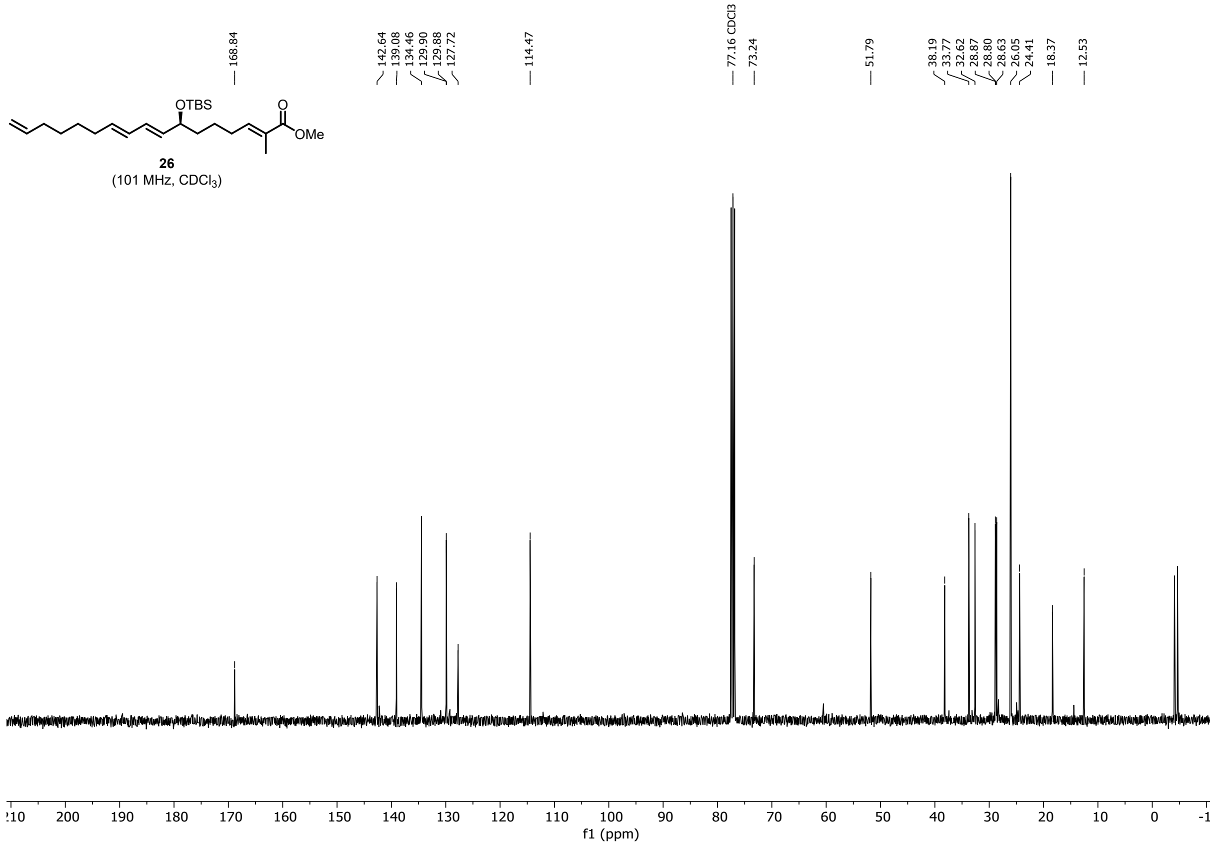
168.84
142.64
139.08
134.46
129.90
129.88
127.72
114.47
77.16 CDCl3
73.24
51.79
38.19
33.77
32.62
28.87
28.80
28.63
26.05
24.41
18.37
12.53
OTBS
O
OMe
26
(101 MHz, CDCl3)
f1 (ppm)

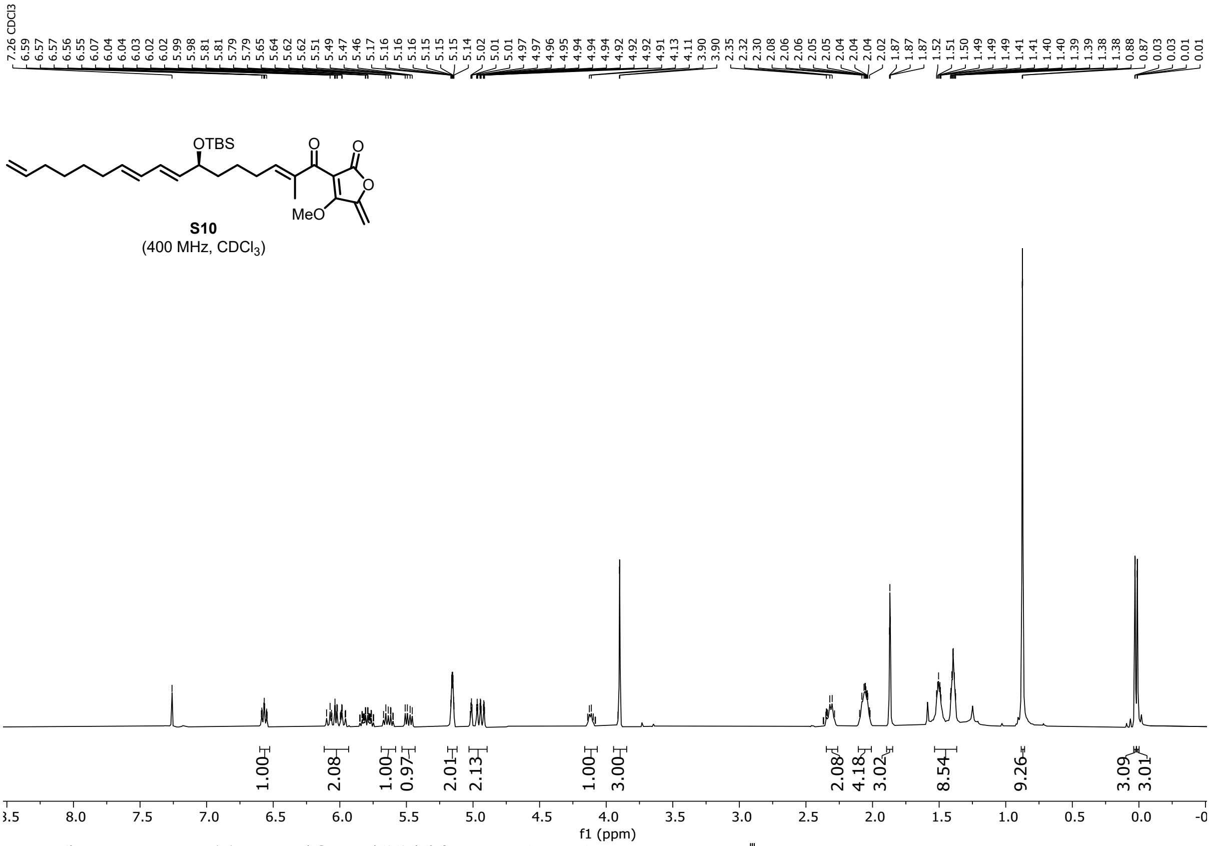
OTBS
MeO
O
S10
(400 MHz, CDCl3)
1.00
2.08
1.00
0.97
2.01
2.13
1.00
3.00
2.08
4.18
3.02
8.54
9.26
3.09
3.01
f1 (ppm)

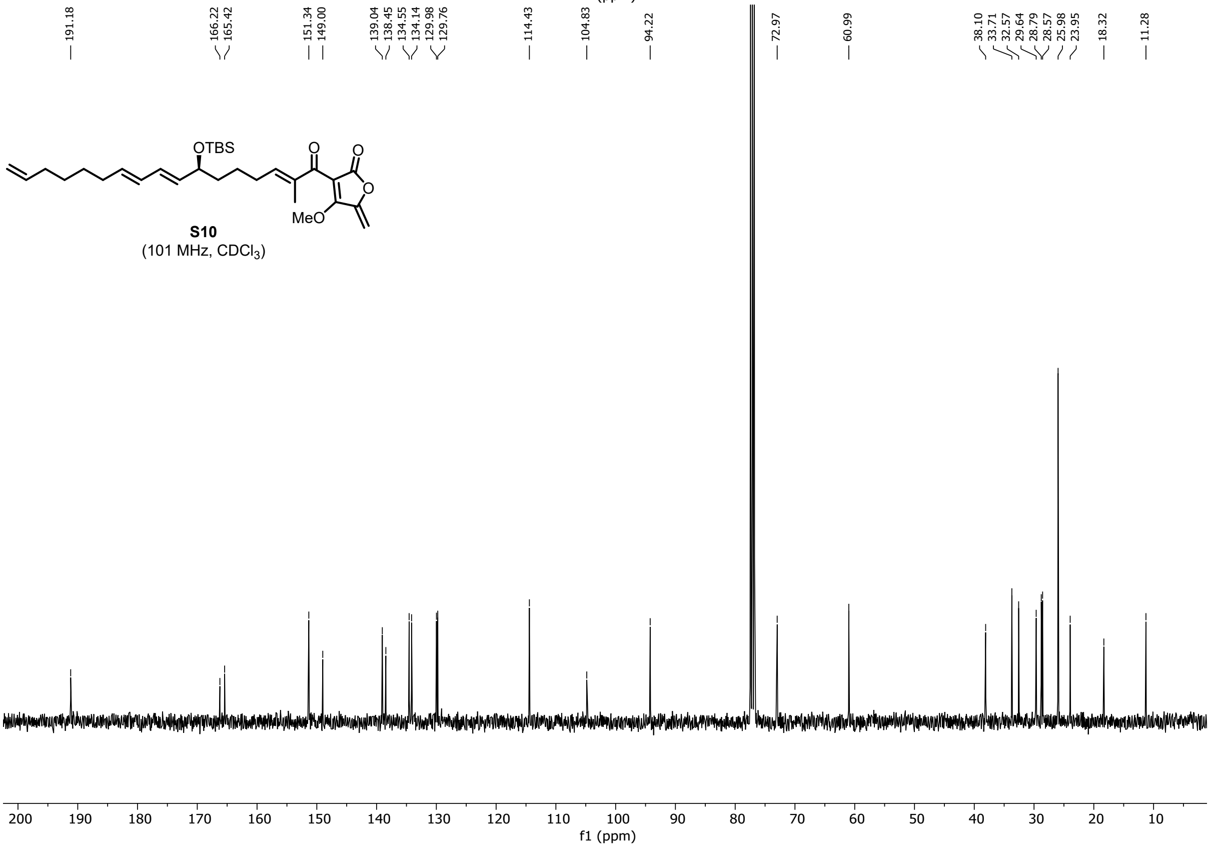
191.18
166.22
165.42
151.34
149.00
139.04
138.45
134.55
134.14
129.98
129.76
114.43
104.83
94.22
72.97
60.99
38.10
33.71
32.57
29.64
28.79
28.57
25.98
23.95
18.32
11.28
OTBS
MeO
O
S10
(101 MHz, CDCl3)
f1 (ppm)

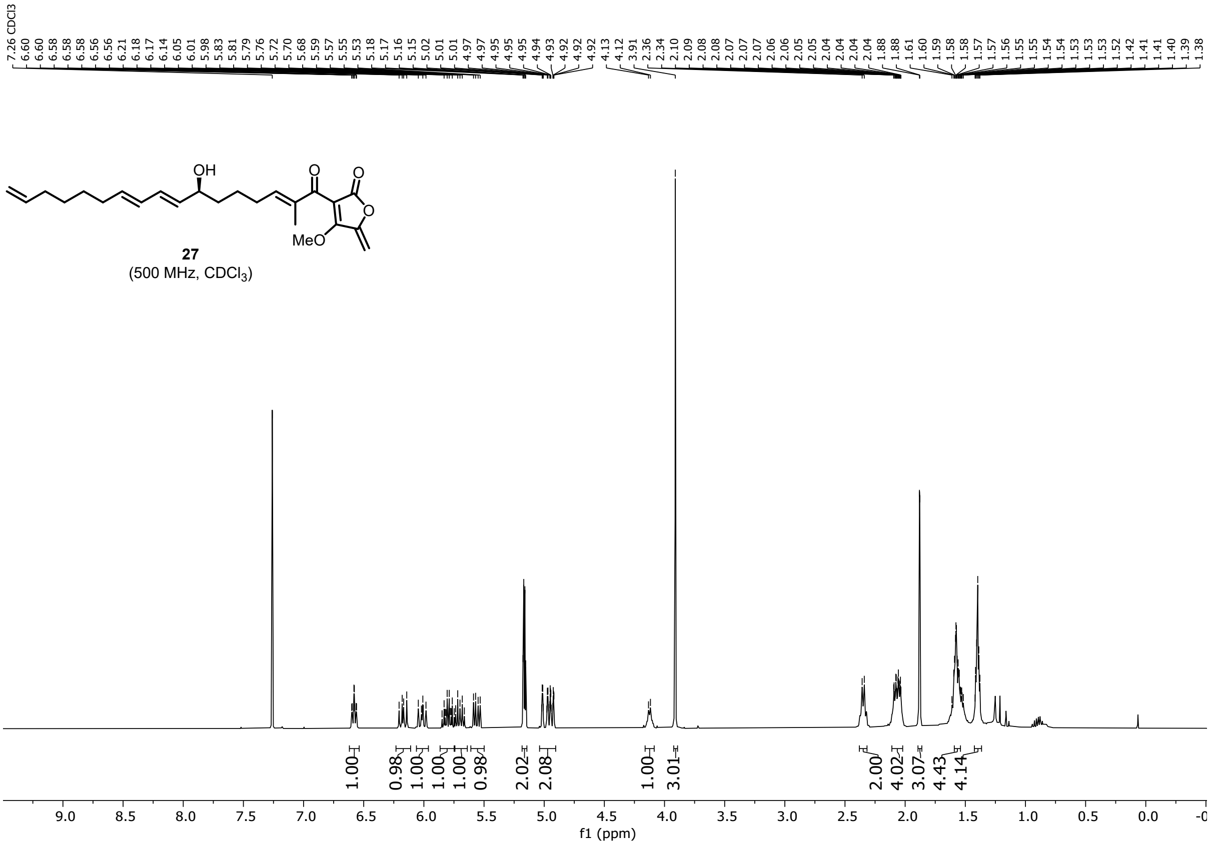
7.26 CDCl3
6.60 6.60 6.58 6.58 6.56 6.56 6.21 6.18 6.17 6.14 6.05 6.01 5.98 5.83 5.81 5.79 5.76 5.72 5.70 5.68 5.59 5.57 5.55 5.53 5.18 5.17 5.16 5.15 5.02 5.01 5.01 4.97 4.97 4.95 4.95 4.94 4.93 4.92 4.92 4.92 4.13 4.12 3.91 2.36 2.34 2.10 2.09 2.08 2.08 2.07 2.07 2.07 2.06 2.06 2.05 2.05 2.04 2.04 2.04 2.04 1.88 1.88 1.61 1.60 1.59 1.58 1.58 1.57 1.57 1.56 1.55 1.55 1.54 1.54 1.53 1.53 1.53 1.52 1.42 1.41 1.41 1.40 1.39 1.38
OH
O
O
O
MeO
27
(500 MHz, CDCl3)
1.00 0.98 1.00 1.00 1.00 0.98 2.02 2.08 1.00 3.01 2.00 4.02 3.07 4.43 4.14
9.0 8.5 8.0 7.5 7.0 6.5 6.0 5.5 5.0 4.5 4.0 3.5 3.0 2.5 2.0 1.5 1.0 0.5 0.0
f1 (ppm)

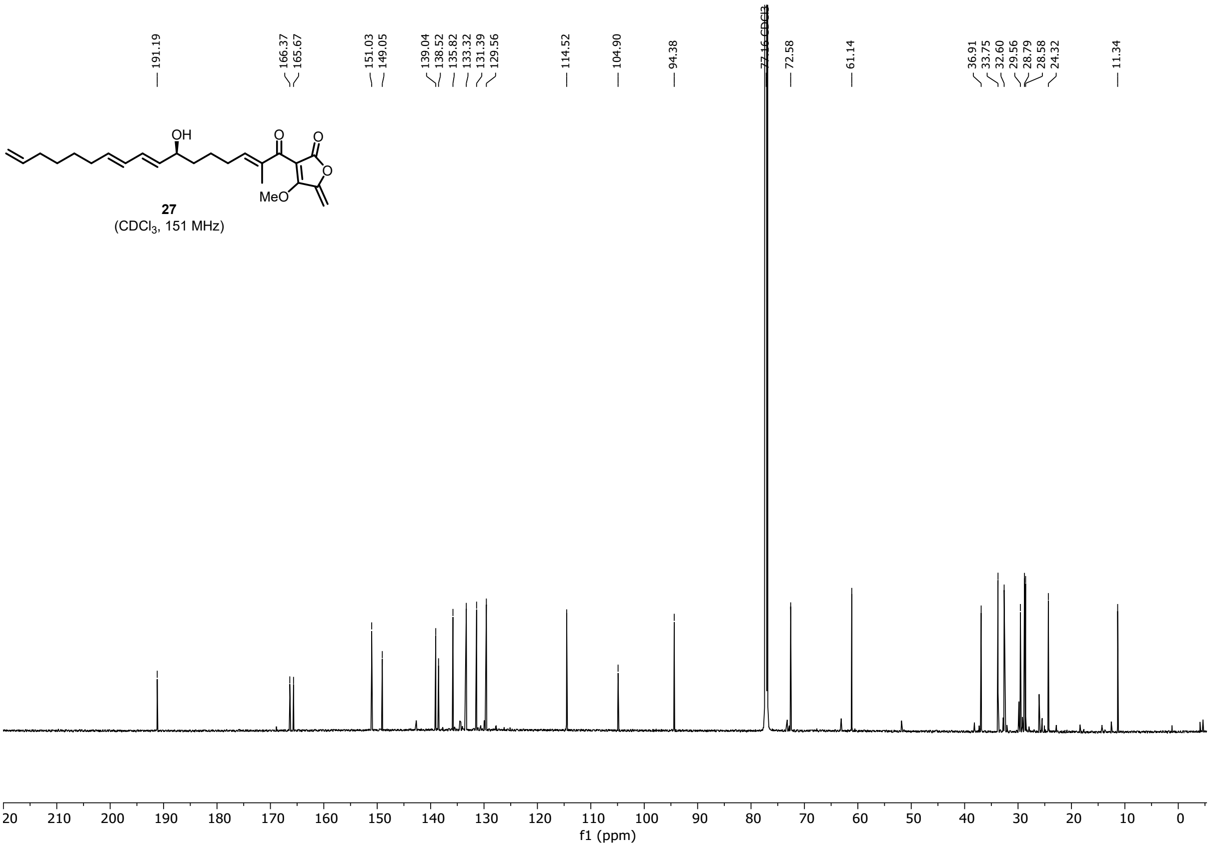
191.19
166.37 165.67
151.03 149.05
139.04 138.52 135.82 133.32 131.39 129.56
114.52
104.90
94.38
77.16 CDCl3
72.58
61.14
36.91 33.75 32.60 29.56 28.79 28.58 24.32
11.34
OH
O
O
O
MeO
27
(CDCl3, 151 MHz)
210 200 190 180 170 160 150 140 130 120 110 100 90 80 70 60 50 40 30 20 10 0
f1 (ppm)

6.2 Enzymatic Reaction Products

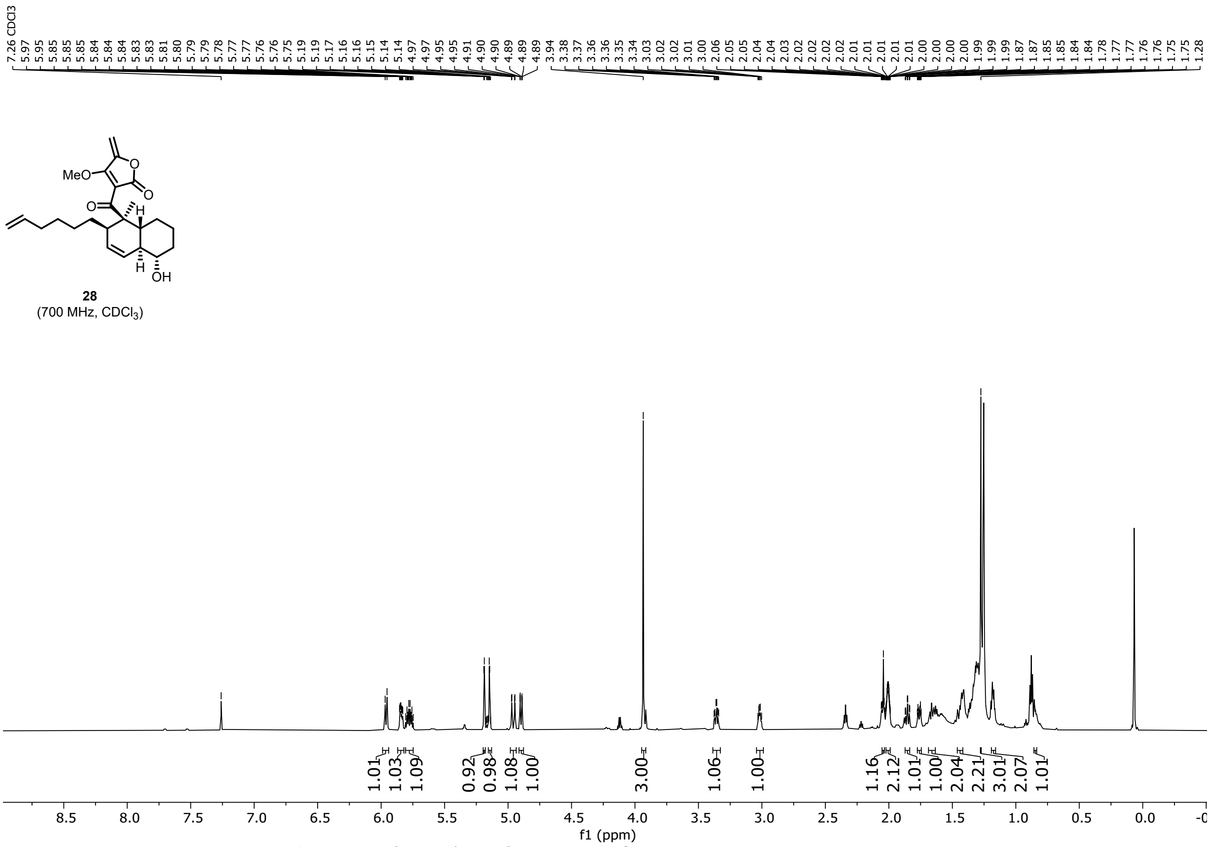

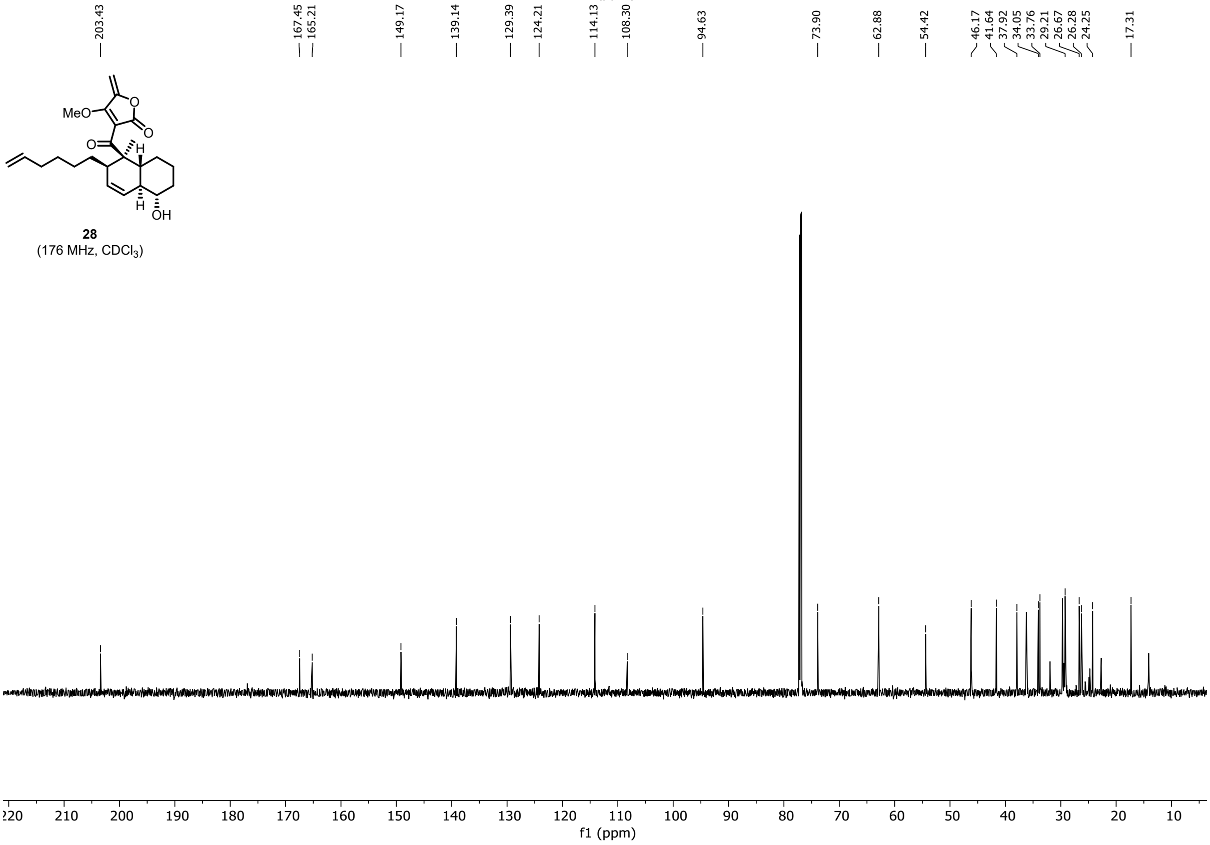

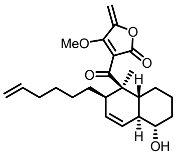

28
(^{1}H-^{1}H COSY)

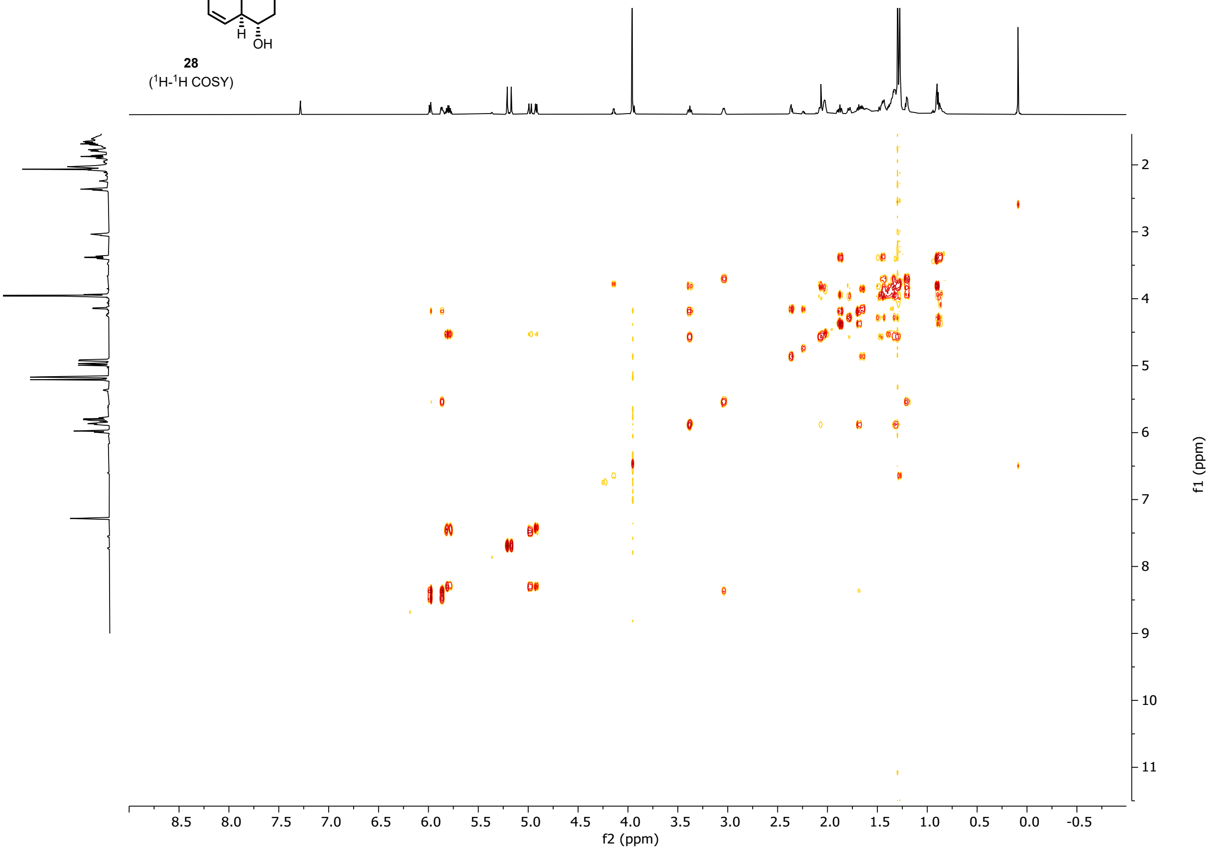

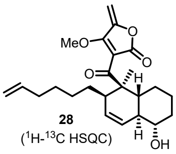

28
(^{1}H-^{13}C HSQC)

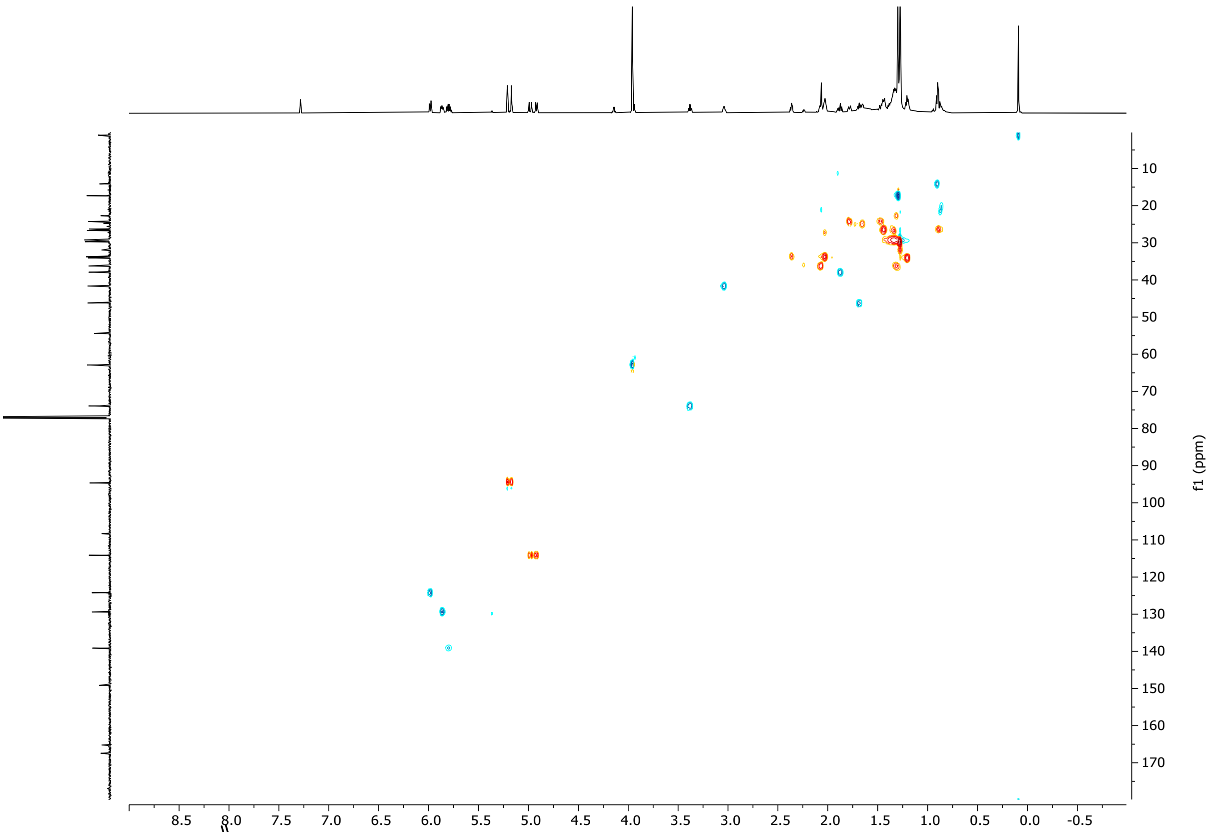

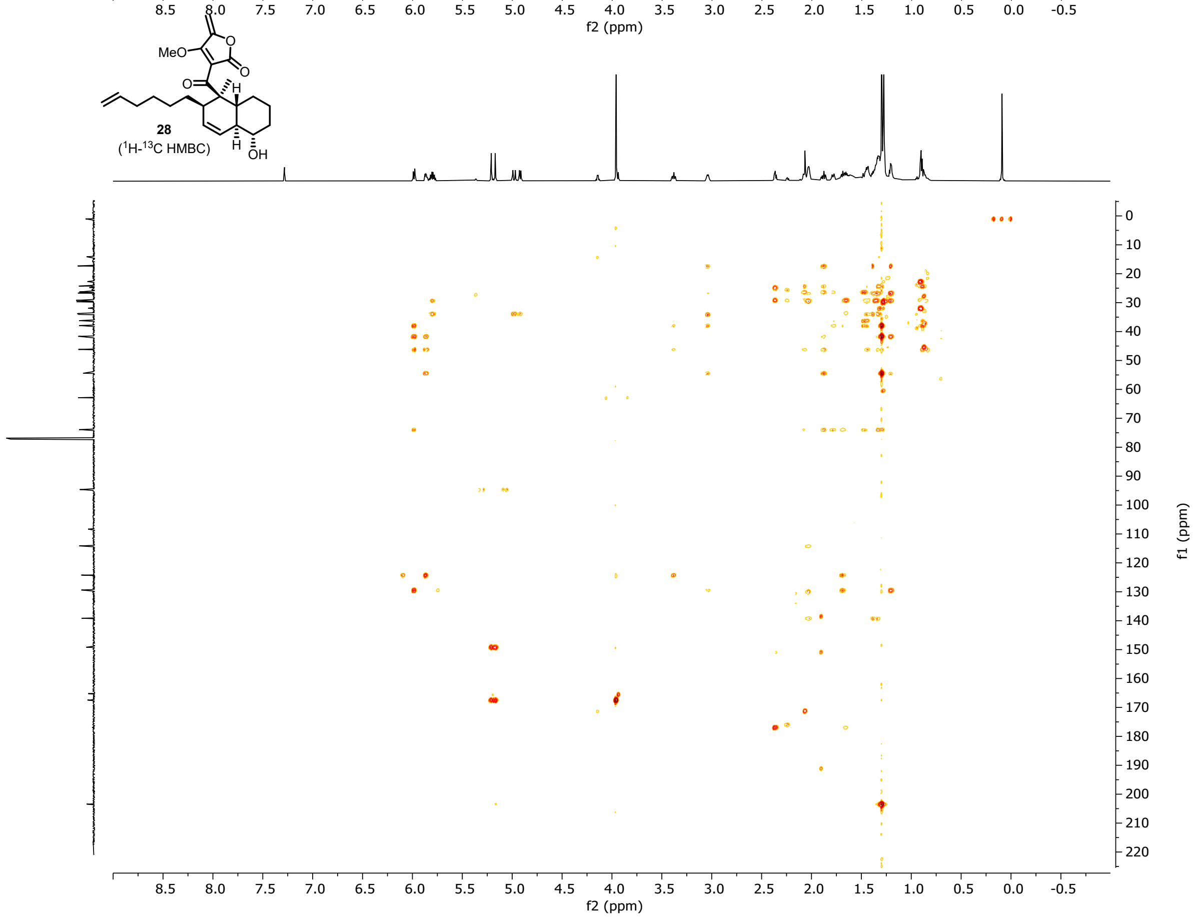

28
(^{1}H-^{13}C HMBC)

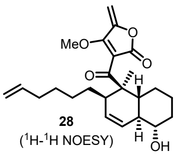

28
(^{1}H-^{1}H NOESY)

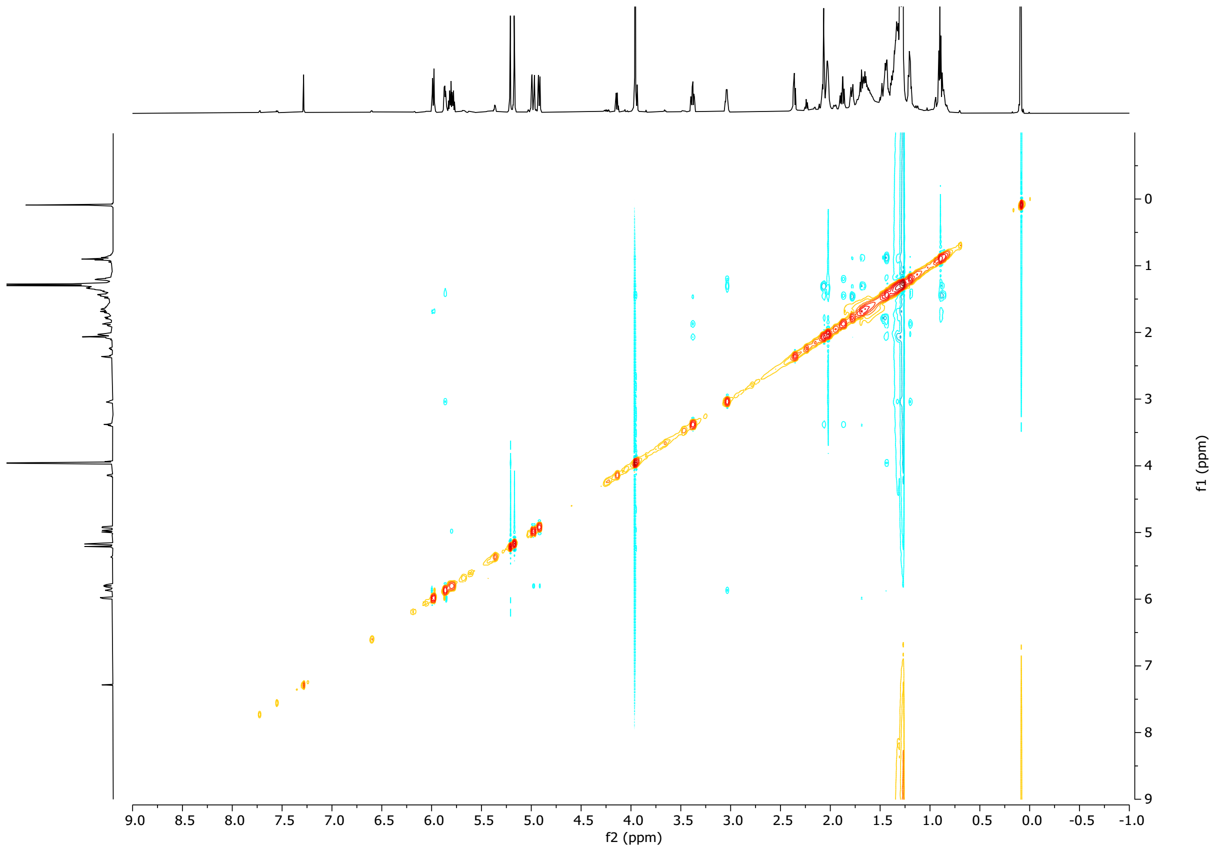
f1 (ppm)
f2 (ppm)
9.0 8.5 8.0 7.5 7.0 6.5 6.0 5.5 5.0 4.5 4.0 3.5 3.0 2.5 2.0 1.5 1.0 0.5 0.0 -0.5 -1.0
0 1 2 3 4 5 6 7 8 9

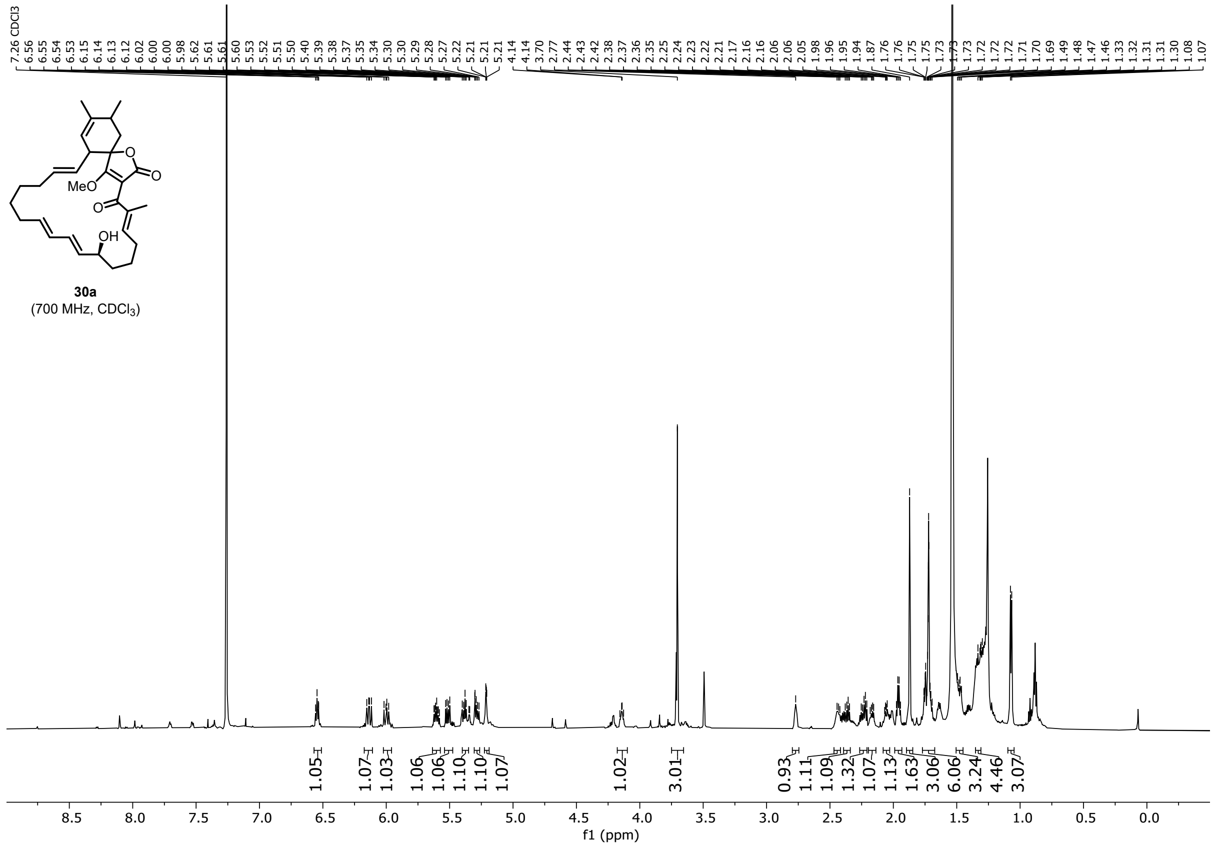
30a
(700 MHz, CDCl3)
f1 (ppm)

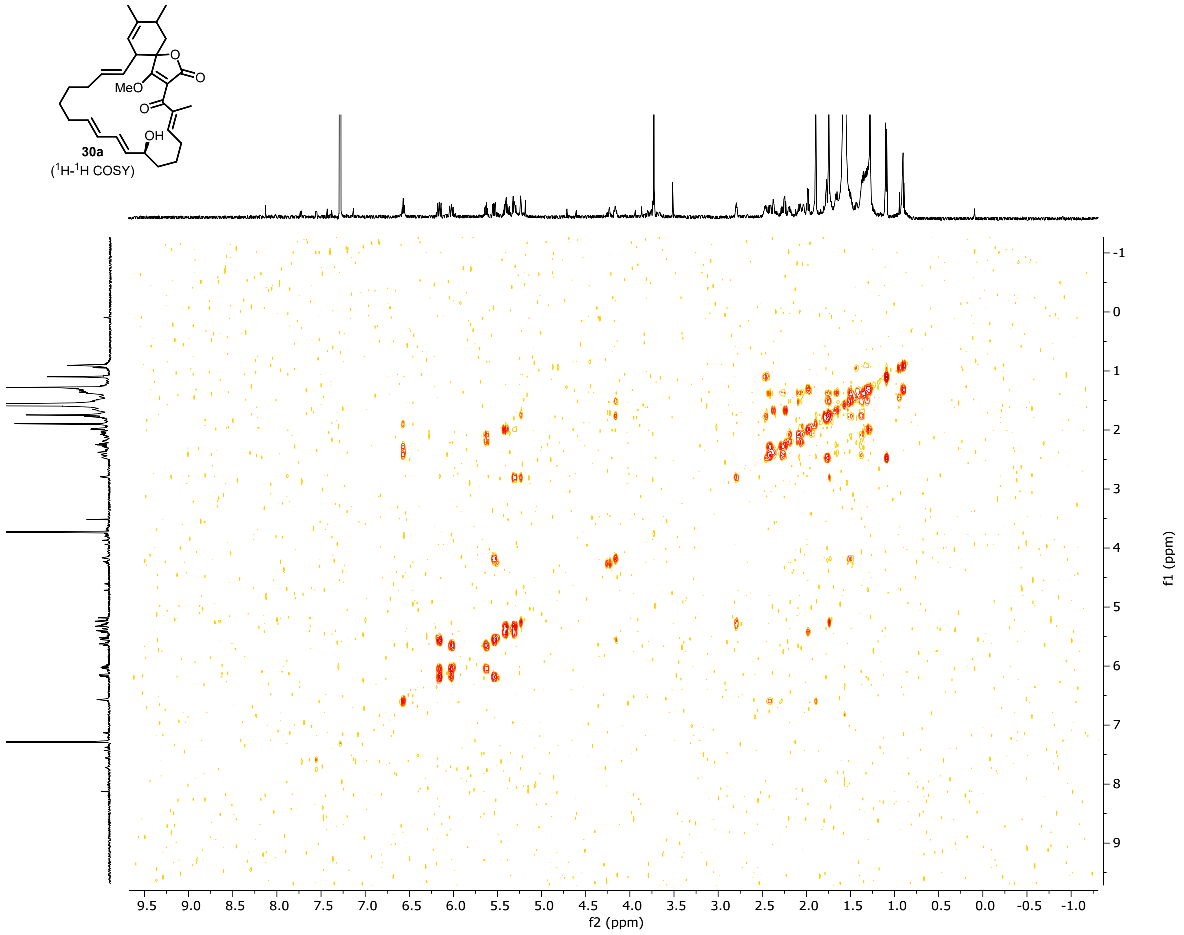
30a
(1H-1H COSY)
f2 (ppm)
f1 (ppm)

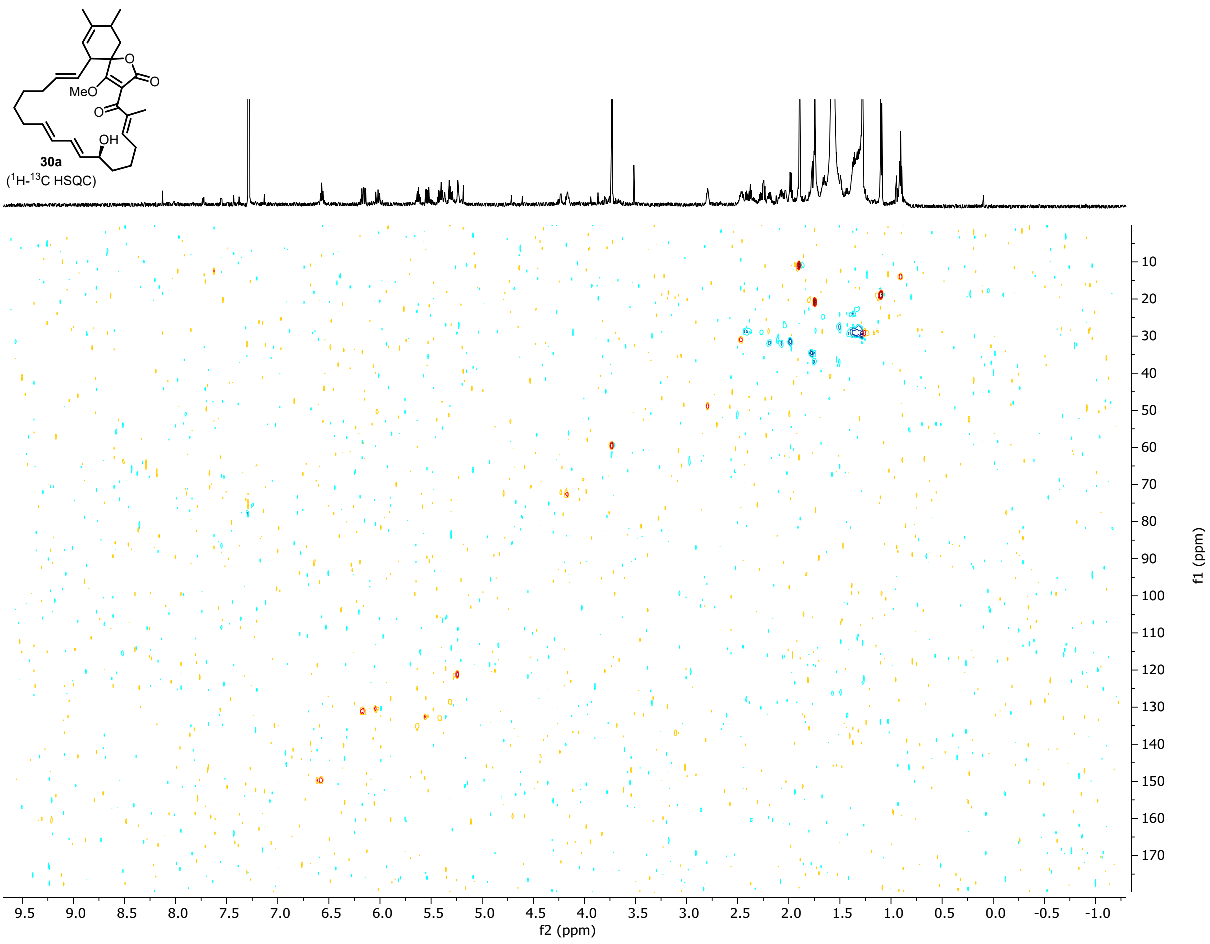
MeO
O
O
O
OH
30a
(1H-13C HSQC)
f2 (ppm)
f1 (ppm)

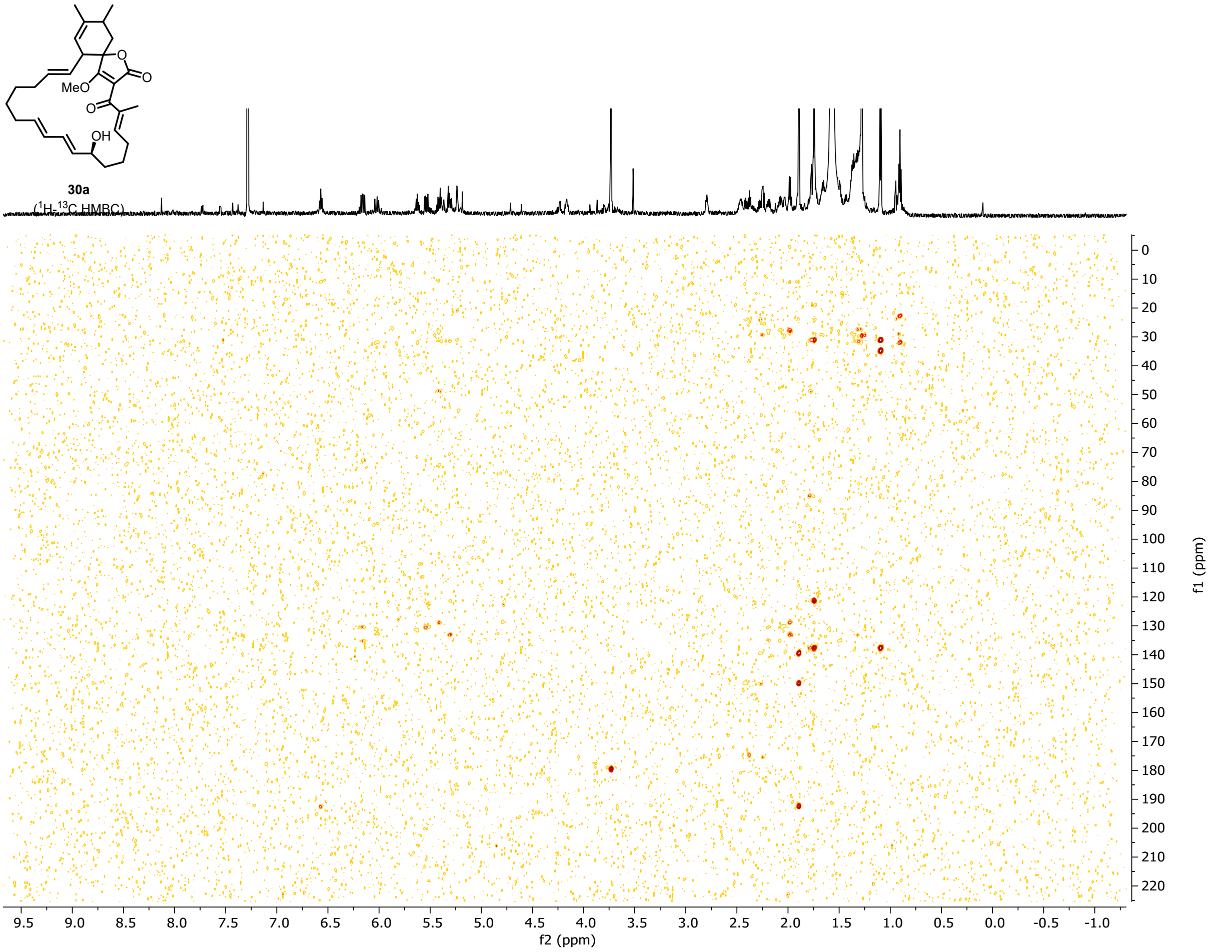
MeO
O
O
O
OH
30a
(1H-13C HMBC)
f2 (ppm)
f1 (ppm)

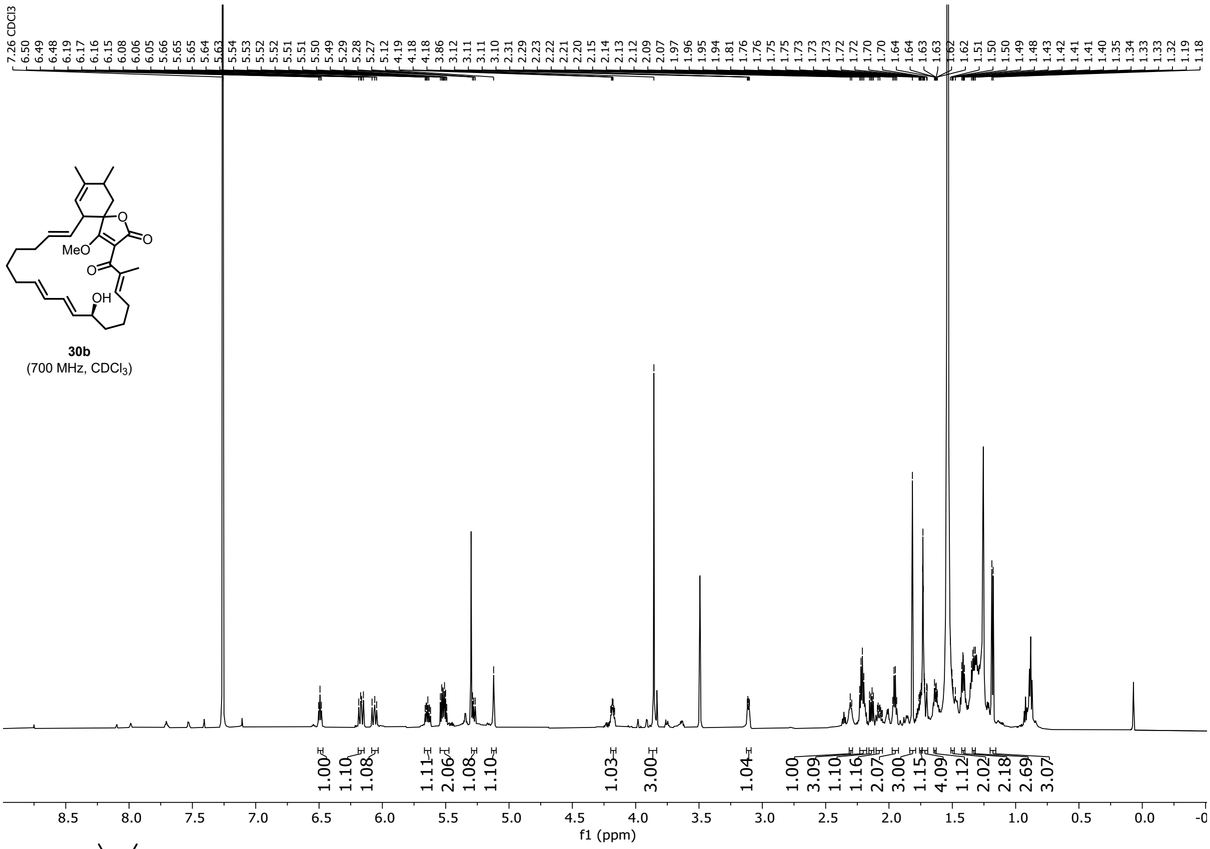

30b
(700 MHz, $CDCl_3$)

30b
(^1H-^1H COSY)

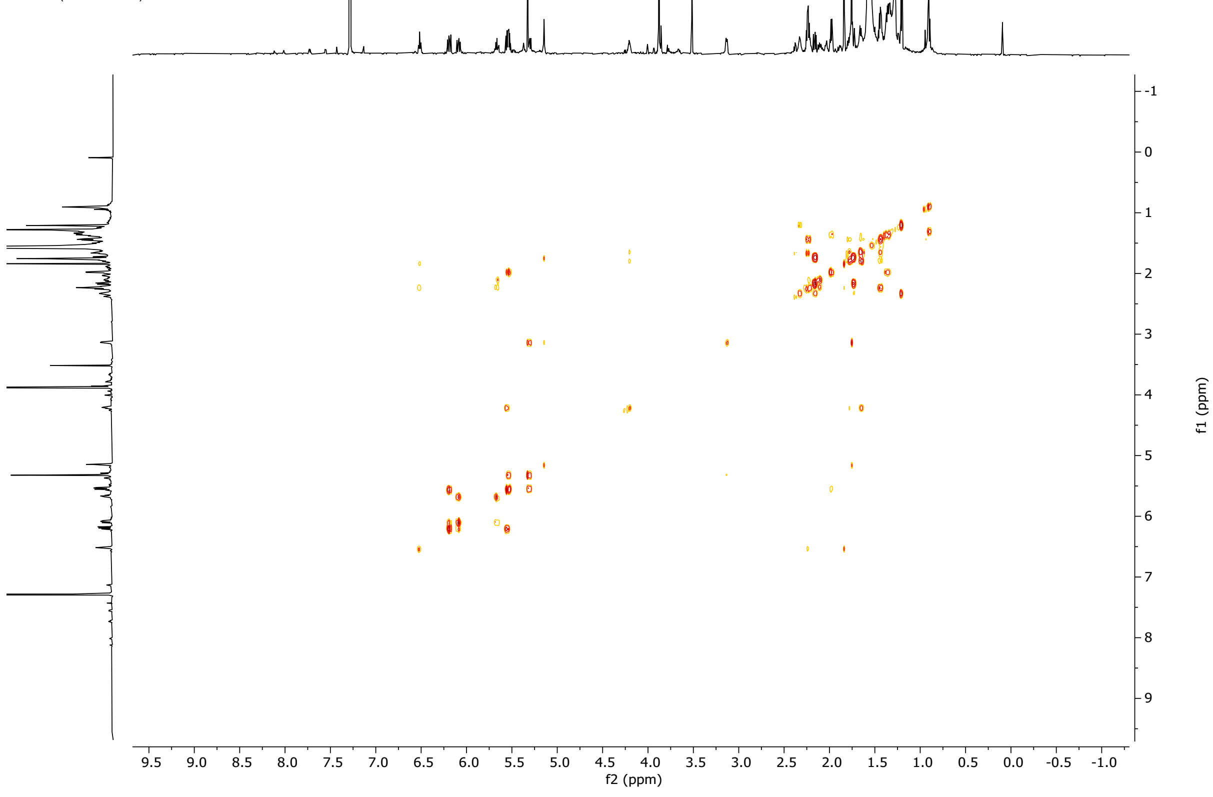

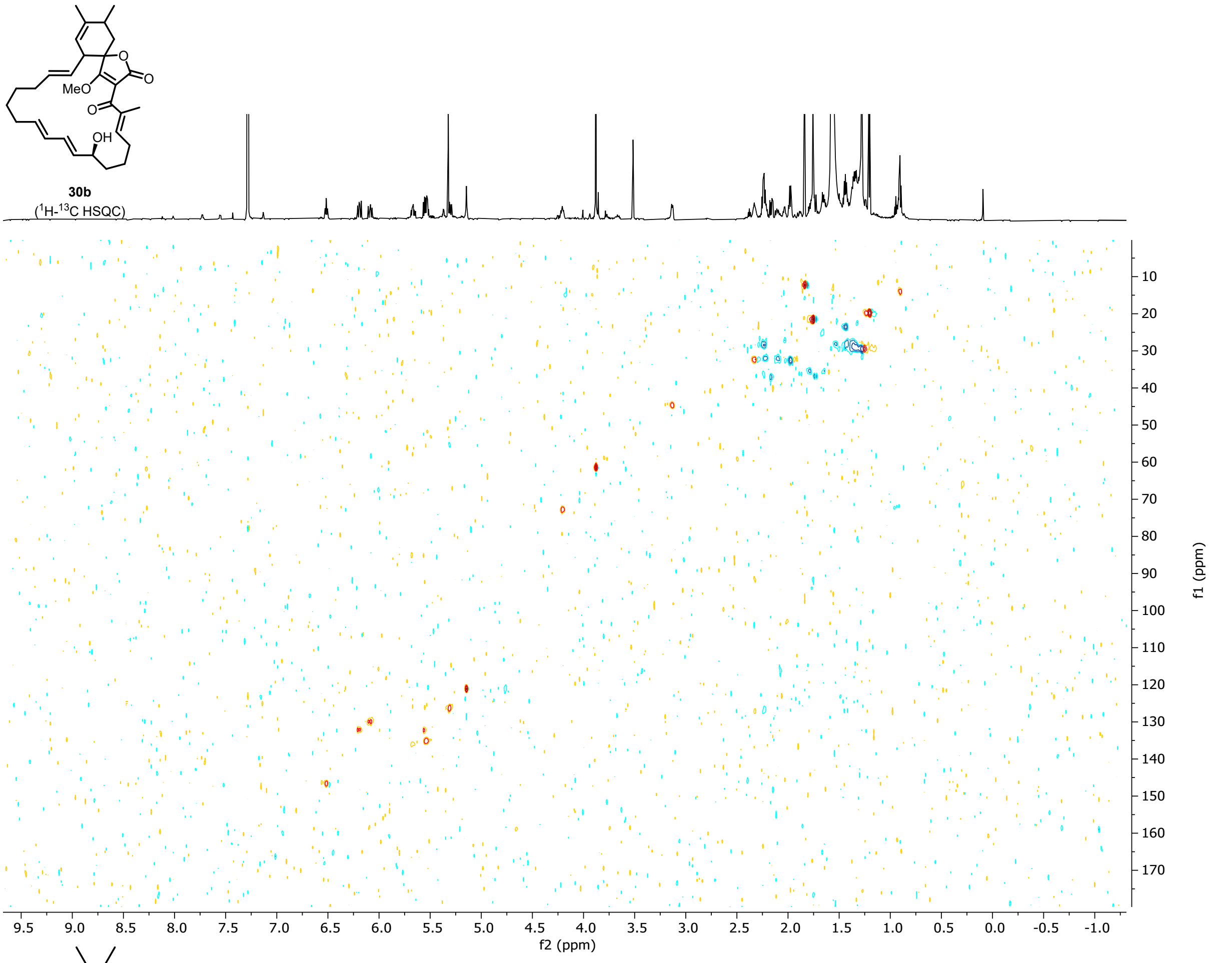

30b
(^{1}H-^{13}C HSQC)

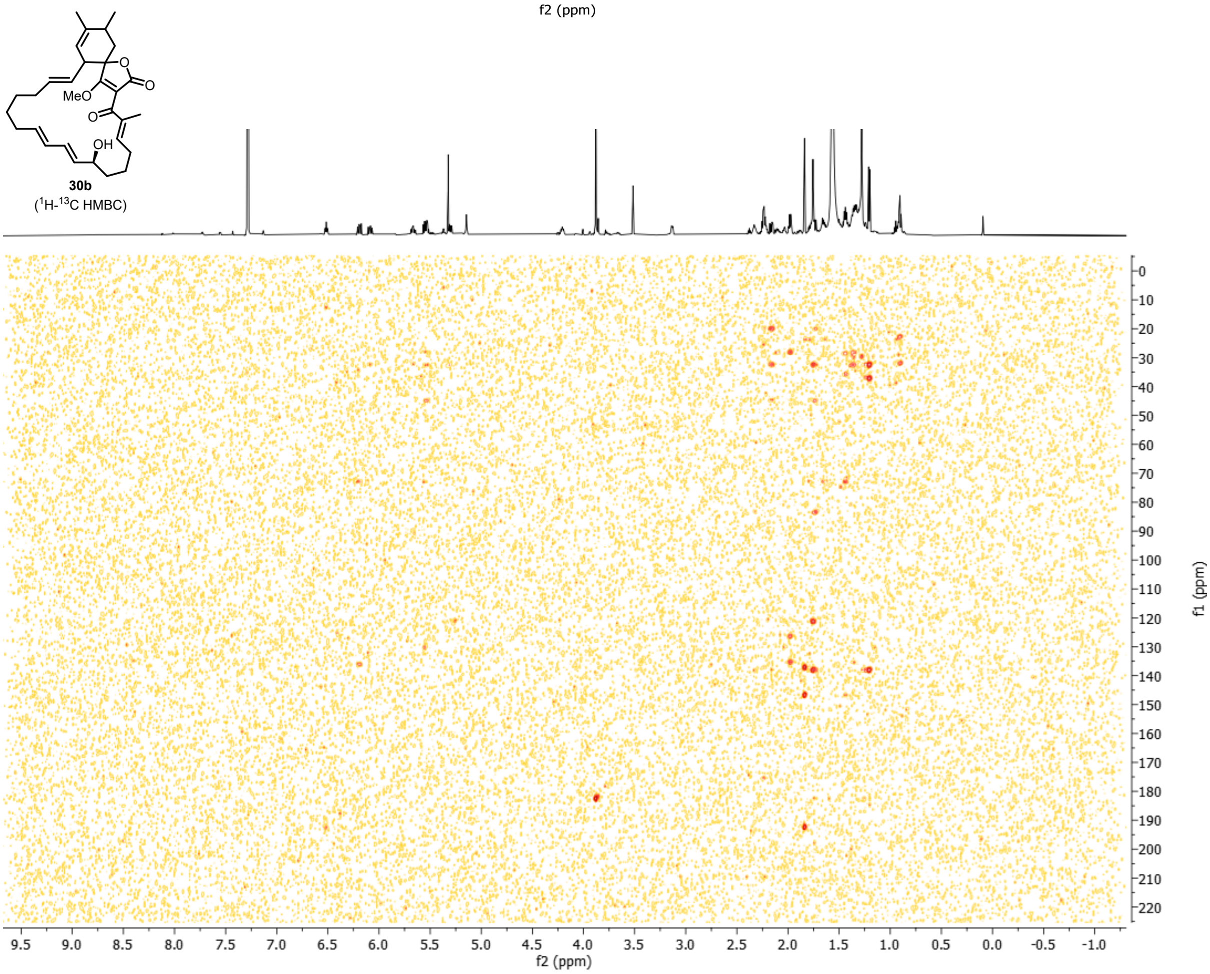

30b
(^{1}H-^{13}C HMBC)

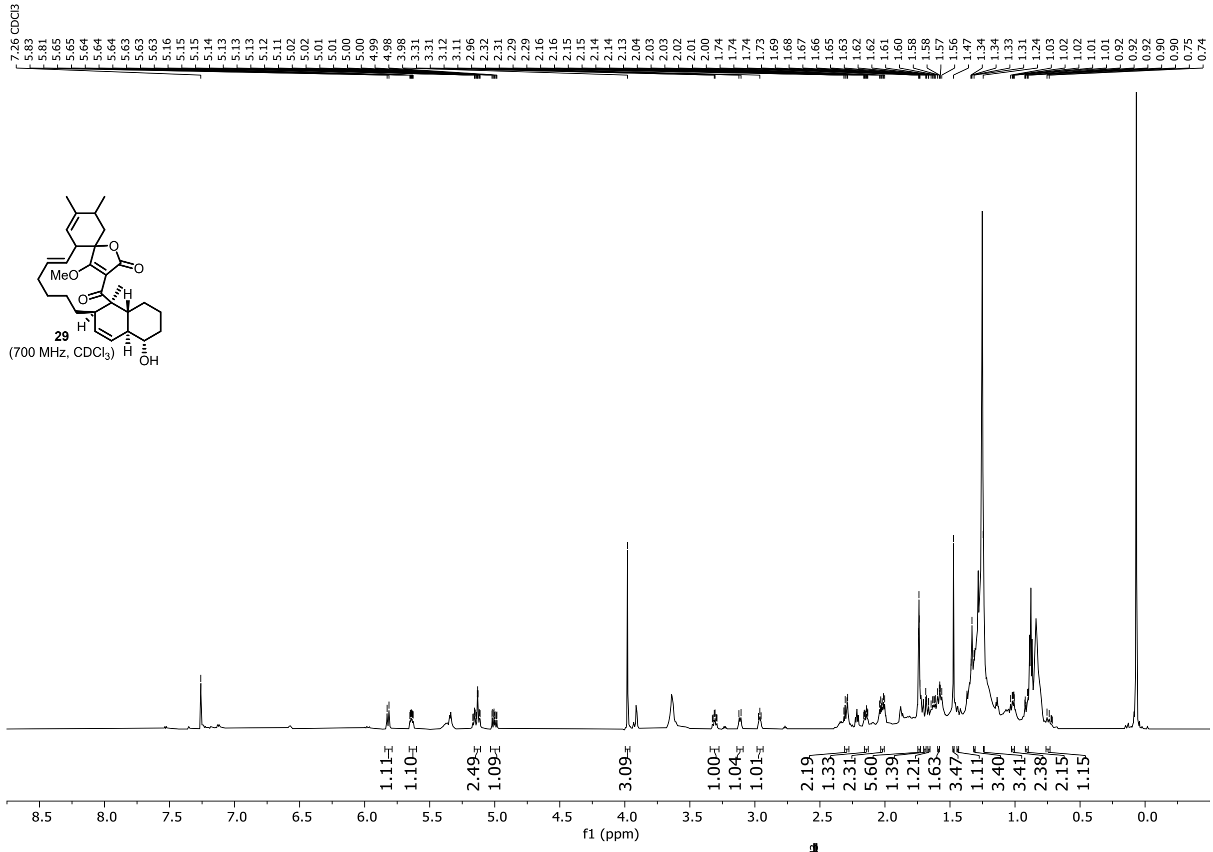
7.26 CDCl3
5.83 5.81 5.65 5.64 5.64 5.64 5.63 5.63 5.63 5.16 5.15 5.15 5.14 5.13 5.13 5.13 5.12 5.11 5.02 5.02 5.01 5.01 5.00 5.00 4.99 4.98 3.98 3.31 3.31 3.12 3.11 2.96 2.32 2.31 2.29 2.29 2.16 2.16 2.15 2.15 2.14 2.14 2.13 2.04 2.03 2.03 2.02 2.02 2.01 2.00 1.74 1.74 1.74 1.73 1.69 1.68 1.67 1.66 1.65 1.63 1.62 1.62 1.61 1.60 1.58 1.58 1.57 1.56 1.47 1.34 1.34 1.33 1.31 1.24 1.03 1.02 1.02 1.01 1.01 0.92 0.92 0.92 0.90 0.75 0.74
MeO
O
H
29
(700 MHz, CDCl3)
OH
1.11 1.10 2.49 1.09 3.09 1.00 1.04 1.01 2.19 1.33 2.31 5.60 1.39 1.21 1.63 3.47 1.11 3.40 3.41 2.38 2.15 1.15
f1 (ppm)

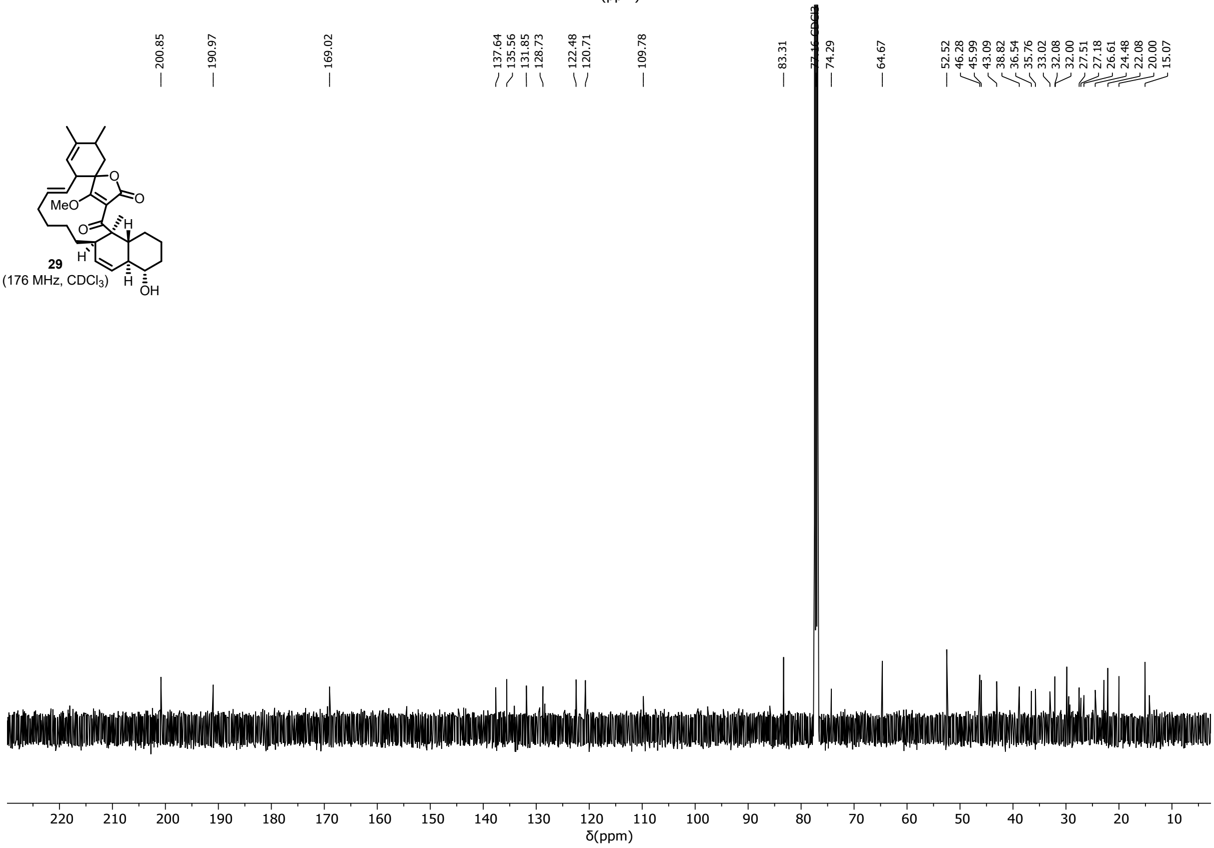
200.85
190.97
169.02
137.64 135.56 131.85 128.73
122.48 120.71
109.78
83.31
77.16 CDCl3
74.29
64.67
52.52 46.28 45.99 43.09 38.82 36.54 35.76 33.02 32.08 32.00 27.51 27.18 26.61 24.48 22.08 20.00 15.07
MeO
O
H
29
(176 MHz, CDCl3)
OH
δ(ppm)

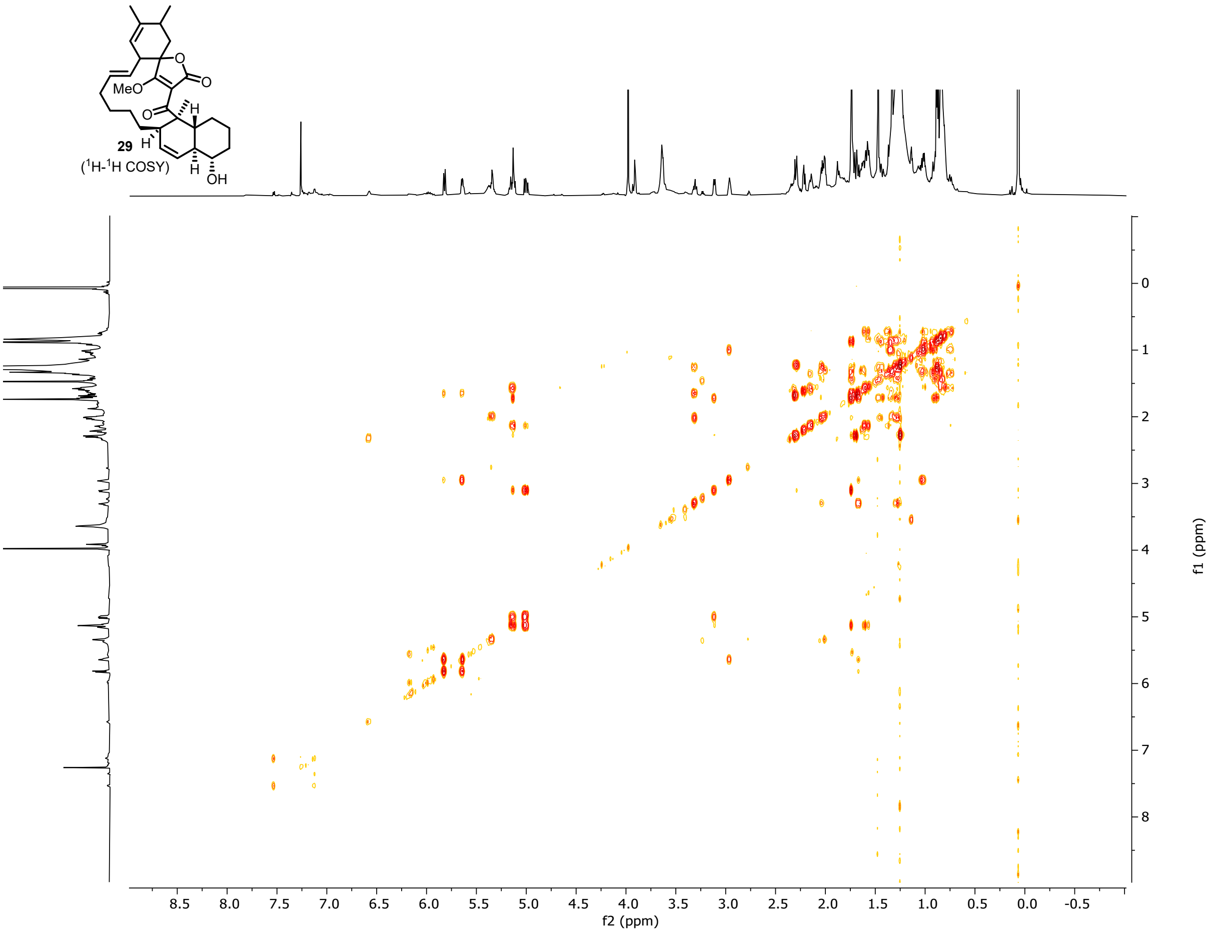

MeO
29
(1H-1H COSY)
OH
f2 (ppm)
f1 (ppm)

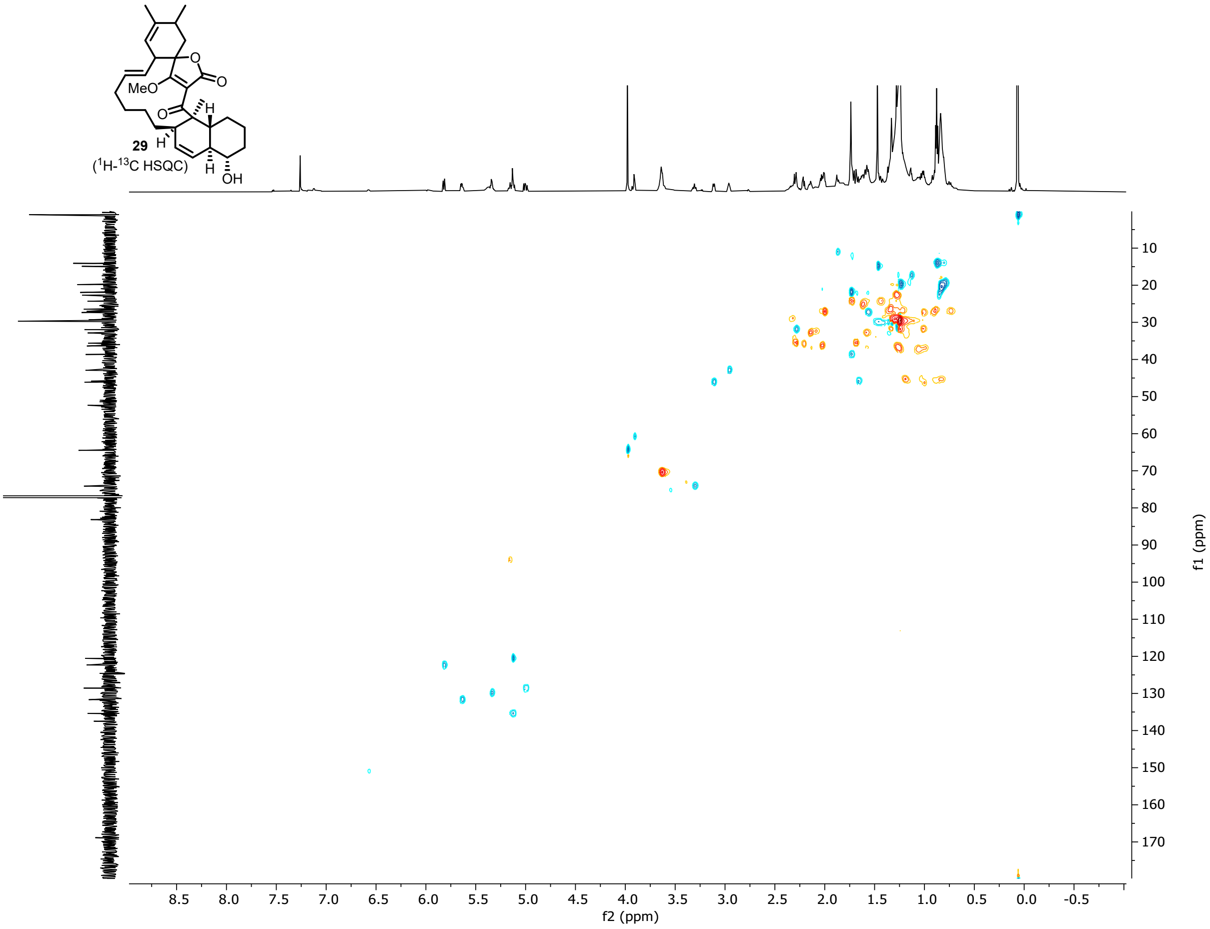

MeO
29
(1H-13C HSQC)
OH
f2 (ppm)
f1 (ppm)

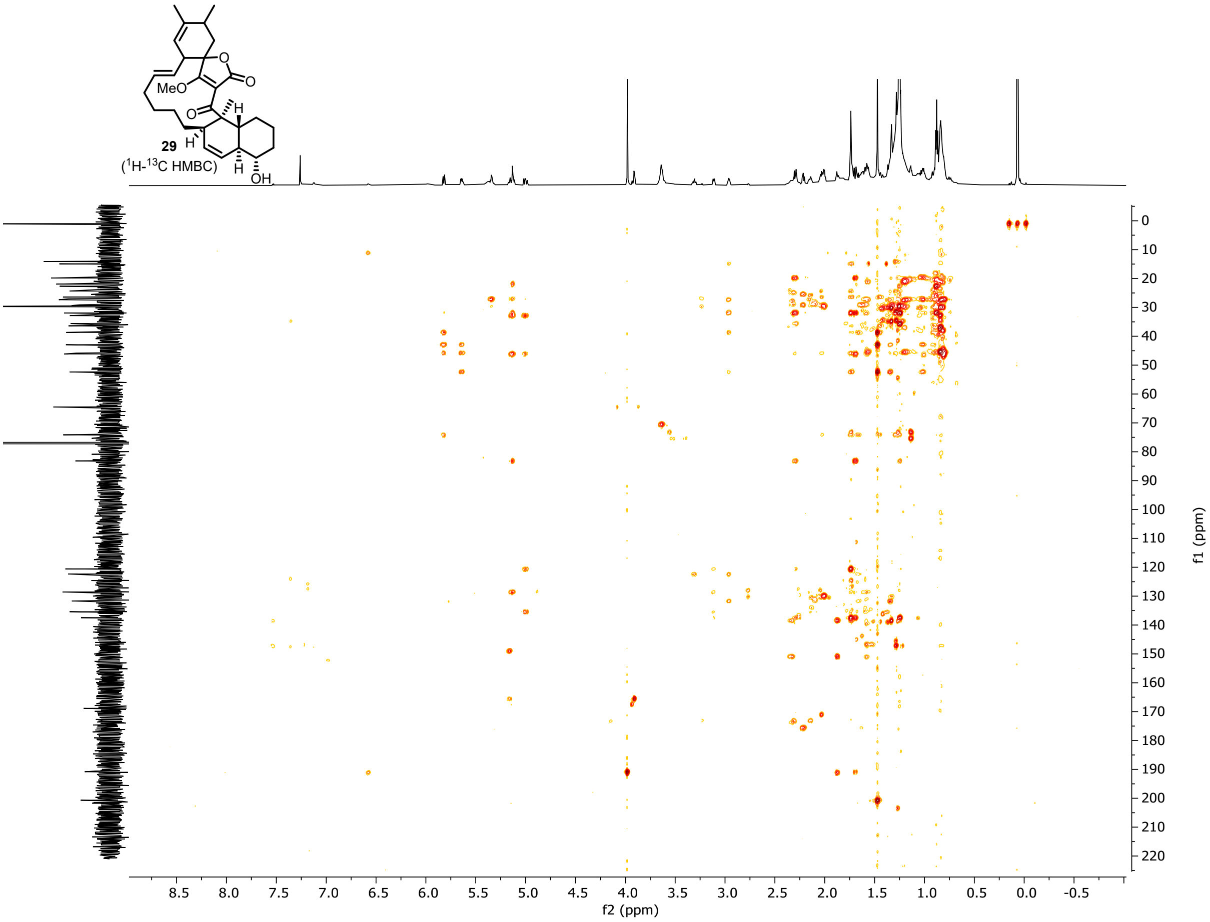
MeO
29
(1H-13C HMBC)
OH
f2 (ppm)
f1 (ppm)

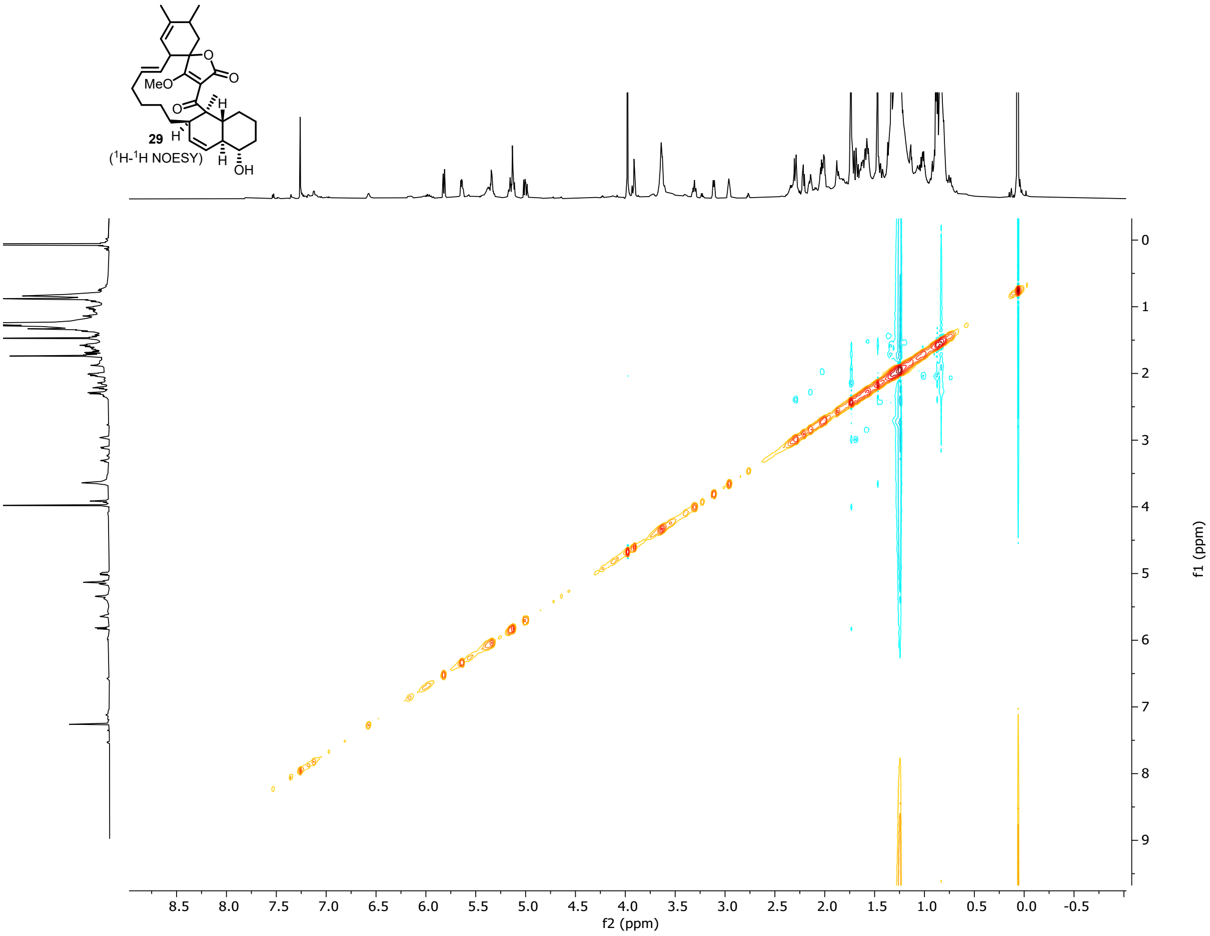
MeO
29
(1H-1H NOESY)
OH
f2 (ppm)
f1 (ppm)

Comparison of ^{1}H-NMR Spectra of Thermal (impure) and ChlE3 Catalysed Diels-Alder Reactions

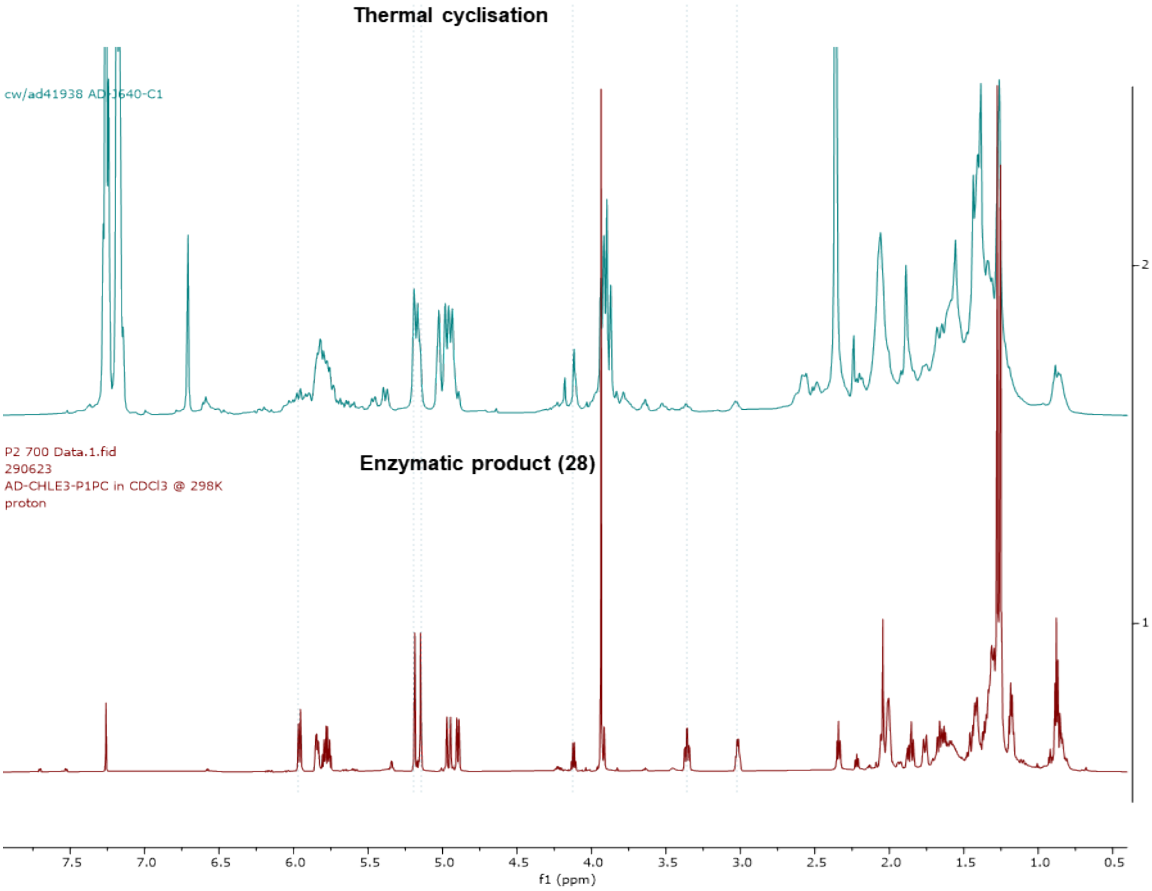

7. Amino Acid Sequences

Expressed ChlE3

MNHSVVVVGAGPVGLMLAAELARAGVPTLVLERRAETGERAPGLAINSAVVELFAQRGIMD
SLQGDGMEFPRAHFAHIWLDPAALAGEHPYTFLVPHHRVAQRLEDHATKAGAQVRRGAEV
IGLRQDASGAELDVRWDGGTEVIRAAYVVGCDGAGSAVRRLAGIGFPGVDEVFYGLVGDL
SVEAGDPLFDRLGVHQHDDGFFTVGPVSQNVLRVTTGEFDAAPGDPDAEVTGEELAAHVR
RLTGAELTTRGTPRWLSRWTAATRQAERYREGRVFLAGDAAHVHFPLGGQALSTGIEDAV
NLGWKLAASLADLAPAGLLDTYHEERHPVGARACSTTRAQMTLLRPGSGTGPLRELLTELV
GLGEVNDHLVSLVGGLDIRYASLAGEGAHTLAGRRLPVTEVATDGGPVSVSEVAHSGRGV
LLDLSPGGGLAATVAAAWRERIDMVSGAPVKGLPEGVLLRPDGRVAWAGTAADGTGLTEA
AARWFGPGTAGRDRRDLEHHHHHH

Expressed ChlL

MTVGAAAPEGLDRRALDSGQLAFQGQMPATSLVEPRDLALAAAAAAGIYDPAKDSAEEALK
KCVIVEGLTEVIEKMAIHDEGTHGAAALAEYQDGFFDADGNRVGTVIGSARVLSMAPHMWQ
YHQSRTEFEGGTFETHGVIDGTAILHGFTQIFQLTGKTGRFAGKAGFMTLTIDDPTQRPPRY
RTSFAMALEHHHHHH
